# Chimpanzees select comfortable nesting tree species

**DOI:** 10.1038/s41598-023-44192-6

**Published:** 2023-10-07

**Authors:** Camille Lacroux, Sabrina Krief, Stéphane Douady, Raphaël Cornette, Sophie Durand, Alfred Aleeje, Edward Asalu, Emmanuelle Pouydebat

**Affiliations:** 1 UMR 7206 CNRS/MNHN/P7, Eco-anthropologie, Museum National d’Histoire Naturelle, Musée de L’Homme, 17 Place du Trocadéro, 75116 Paris, France; 2 UMR 7179 CNRS/MNHN, Mécanismes Adaptatifs et Evolution, Museum National d’Histoire Naturelle, 57 Rue Cuvier, 75231 Paris, France; 3 Sebitoli Chimpanzee Project, Great Ape Conservation Project, Kibale National Park, Fort Portal, Uganda; 4 La Phocéenne de Cosmétique, ZA Les Roquassiers, 174 Rue de La Forge, 13300 Salon-de-Provence, France; 5 Matière et Systèmes Complexes, Université Paris-Diderot, 75025 Paris Cedex 13, France; 6 UMR 7205 CNRS/MNHN/SU/EPHE/UA, Institut de Systématique, Évolution, Biodiversité, Museum National d’Histoire Naturelle, CP50 Paris, France; 7 Uganda Wildlife Authority, Kibale National Park, Kibale, Uganda

**Keywords:** Animal behaviour, Biomechanics

## Abstract

Every evening, chimpanzees build sleeping “nests” in trees. In some studied communities, individuals appear to be selective about the tree species used, which has led researchers to hypothesize whether chimpanzees prefer trees that repel troublesome insects or/and that provide comfortable and stable structures. We investigate these hypotheses, or a trade-off between both, though study of tree species preference based on their biomechanical and/or biochemical properties in the Sebitoli chimpanzee community in Kibale National Park, Uganda. The ten tree species most frequently used for nesting were compared with ten abundant in their environment but not preferred for nesting. For these 20 tree species, we determined their biomechanical and morphological characteristics such as foliar density, foliar units form (shape and size) and branch rigidity. Their spatial repellent activity, previously tested against *Anopheles gambiae* was incorporated into the analysis. Chimpanzees chose tree species with medium-sized and elongated foliar units, high foliar density and branch with stiffer wood. In addition, most tree species with such mechanical and morphological properties also have mosquito repellent activity. These tree properties may provide a comfortable sleeping environment enhancing sleep quality. Finally, a comparison across chimpanzee communities would be relevant to understand whether these choices are not only ecological but also cultural.

## Introduction

Every evening, great apes build sleeping platforms commonly called ‘nests’ usually in trees in which they spend the night^1^. Weaned individuals bend, break and intertwine leafy branches and twigs to form a circular platform or bowl-shaped structure^2^. Interestingly, some chimpanzee and orangutan communities studied appear to choose particular tree species to build their nests^1,3^. This behavior has raised questions about the drivers of this tree species selectivity. Identifying the determinants of tree species choice could help us understand how chimpanzees improve sleep quality in their nest. Yet, increasing the time spent in active sleep could lead to better memory consolidation^4^, which could be one of the crucial steps in the evolution of hominids and their mental and manipulative abilities^5^. One explanation, inspired by “the pathogen avoidance hypothesis”, is that individuals may prefer trees that release aromatic substances to mask their odor or repel annoying insects that may be vectors of parasites^6^. In addition, a preliminary study in Kibale National Park in Uganda has shown that chimpanzees chose sites for nesting in altitude and at a height in trees where mosquitoes are less abundant^7^, unlike the results obtained in Nimba Mountain in Guinea where no correlation was found between height of nests and mosquito abundance^8^. These studies did not examine the possible influence of the tree species itself. On the other hand, in the Toro-Semliki Wildlife Reserve in Uganda, individuals are shown to choose one specific tree species that experimentally deters flying arthropods in the field^9^. A more recent study in the Sebitoli community^10^, shows that essential oils from leaves of seven out of ten tree species commonly used by chimpanzees for nesting possess spatial repellent activity against *Anopheles gambiae,* an African mosquito, vector of *Plasmodium falciparum*, a parasite responsible of malaria. In parallel, some orangutans have been observed to occasionally add to their nest, branches from a different species having mosquito-repellent activity^11^. Although the function and duration of nests are different, similar nesting plant selectivity is shown in birds. Indeed, some bird species use specific plant fragments with repellent or antimicrobial activities to build or reinforce their nest structure and help control pest and/or pathogen populations^12,13^.

Alternatively, another explanation inspired by “the sleep quality hypothesis”, postulates that individuals choose nesting materials offering the most physically comfortable structure. Indeed, birds and orangutans build a compliant central structure, with thicker, stronger and stiffer outer elements compared to the weaker and more flexible materials used for the inside cup^14,15^. In the chimpanzee case, a study in the Toro-Semliki community has shown that the most selected nesting tree species, which has potential repellent activity, is also considered as more comfortable by providing a firmer and more stable nest with rigid branches and thick foliage with small leaves^16^.

In this study, we jointly examined the two main frameworks known to explain tree species preference in nesting behavior: mechanical comfort and/or chemical repulsion of insects. We analyzed a large data set obtained in the habituated Sebitoli chimpanzee community. This data set included previously published data on the spatial repulsive activity of the ten tree species most used for nesting and ten tree species that are abundant in their environment but not among the most chosen species for nesting^10^. In addition, we characterized these 20 tree species based on their biomechanical and morphological characteristics (distance between nodes, foliar unit shape and size, branch flexural rigidity and flexural elastic modulus). We expect chimpanzees to select nesting trees with a potential trade-off between repellent and comfortable species but also taking into account their availability in their habitat. Repellent abundant tree species could be physically uncomfortable and conversely, so chimpanzee may need to choose or find a compromise between chemical and physical comfort.

## Results

During the two surveys conducted in 2019 and 2021, we measured 916 distances between foliar units and 96 foliar units from 86 trees and 502 branches from 117 trees of the 20 tree species.

### Foliar density

**Figure 1:**
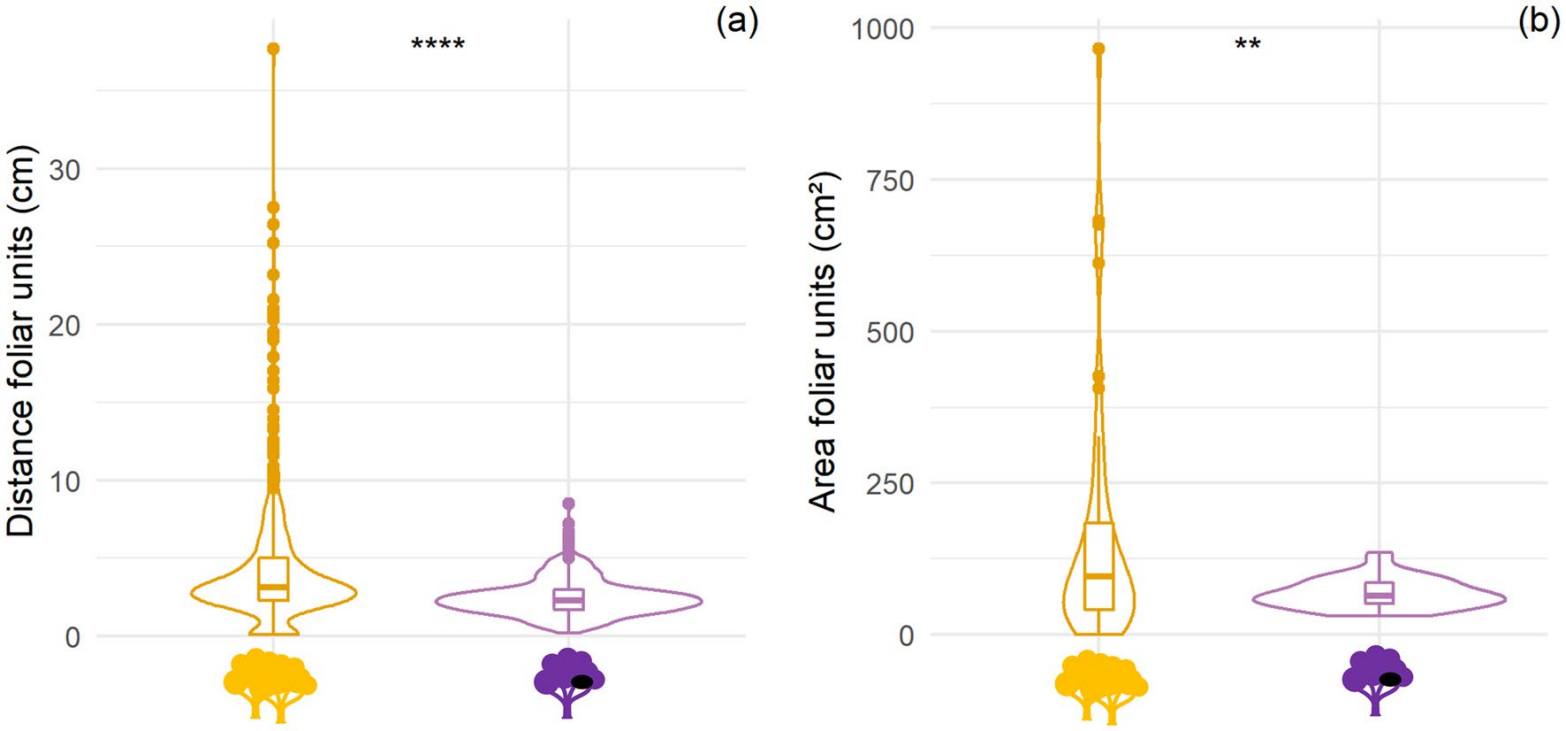
Violin boxplot of distance between foliar units (**a**) and area of foliar units (**b**). *Orange represents abundant tree species not selected by chimpanzees and purple represents nesting tree species.*

The ten nesting tree species tested have on average, smaller distances between foliar units (equivalent to foliar density) compared to 10 tree species available in the habitat yet not preferred by chimpanzees (2.48 ± 1.19 vs. 4.45 ± 4.44 cm; t test, t = 9.32, *df* = 556.11, *p* value < 0.001). In addition, chimpanzees appear to select trees which have a smaller range of distances between foliar units (0.20–8.50 cm) compared to what is available in the habitat (0.10–37.70 cm) (Fig. 1a). Nesting tree species, on average have smaller foliar units compared to abundant tree species (67.87 ± 24.32 vs. 158.81 ± 198.52 cm^2^; t test, t = 3.28, *df* = 52.73, *p* value = 0.002). In addition, chimpanzees appear to select a smaller range of foliar unit area (30.10–134.90 cm^2^) compared to what is available in the habitat (0.02–965.90 cm^2^) (Fig. 1b).

### Foliar unit shape and size

**Figure 2:**
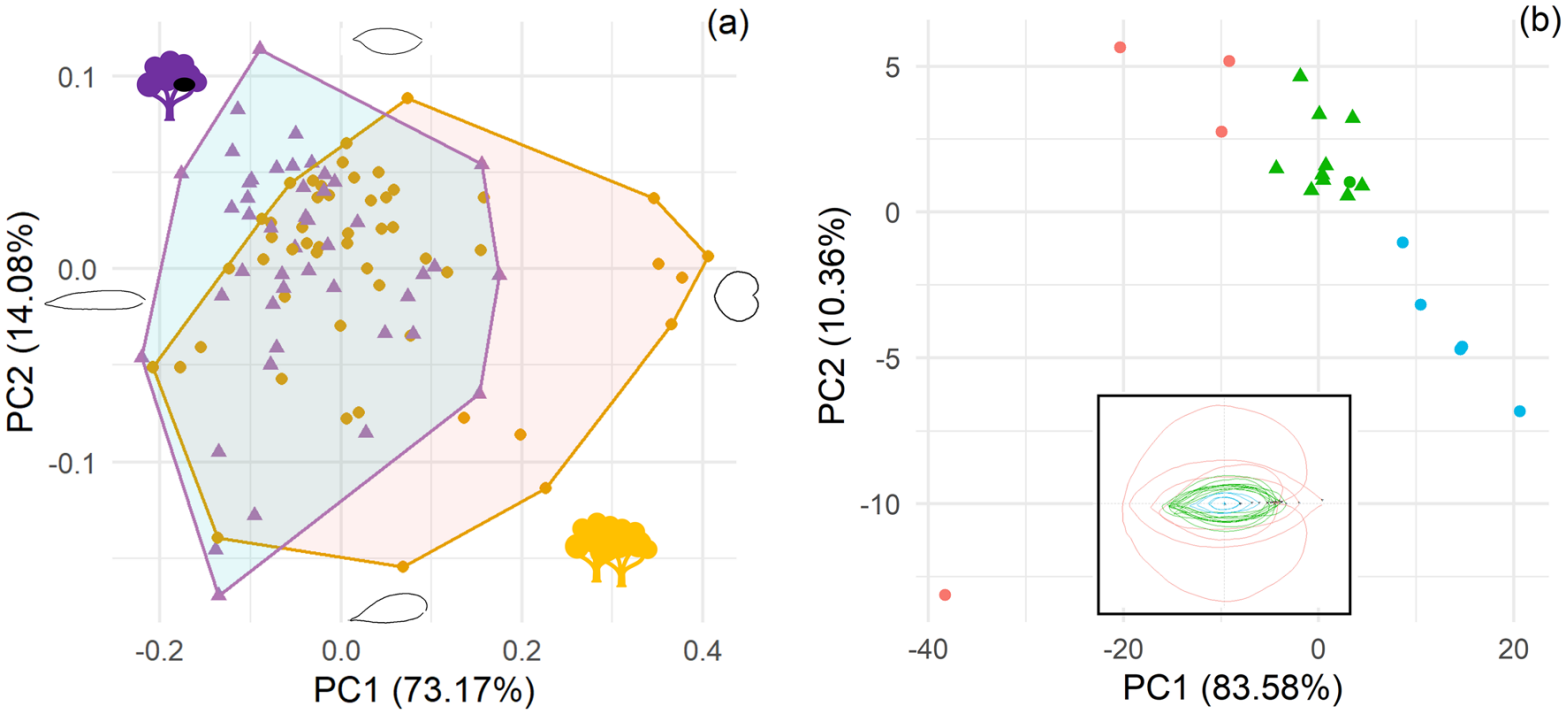
PCA on shape variation with visualization of extreme shape for the two first axis (**a**) and PCA on form variation with foliar form unit per species in the box (**b**). *Points: round for abundant tree species not selected by chimpanzees, triangle represent nesting tree species. Colors: orange represents abundant tree species not selected by chimpanzees, purple represents nesting tree species, red represent "big” foliar units, green represent “middle” foliar units and blue represent “small” foliar units.*

The two first PCA axes explain more than 87% of the total shape variance of 96 aligned foliar unit outlines. The shapes of the extreme values along the first PC axis indicates that much of this variation opposing heart-shaped and elongated foliar units (Fig. 2a). Chimpanzees tree selection is not significantly related to shape alone. When taking into account the size as well, we observe a moderate shape/size covariation in our data set (cor = 0.43, t = 4.67, *df* = 96, *p* value < 0.001). We observed three groups: ‘middle’ shape and elongated foliar units with 11 species that include all ten nesting tree species, ‘small’ elongated foliar units with five species, ‘large’ elongated and hearted foliar units with 4 species (Fig. 2b).

### Branch rigidity

**Figure 3:**
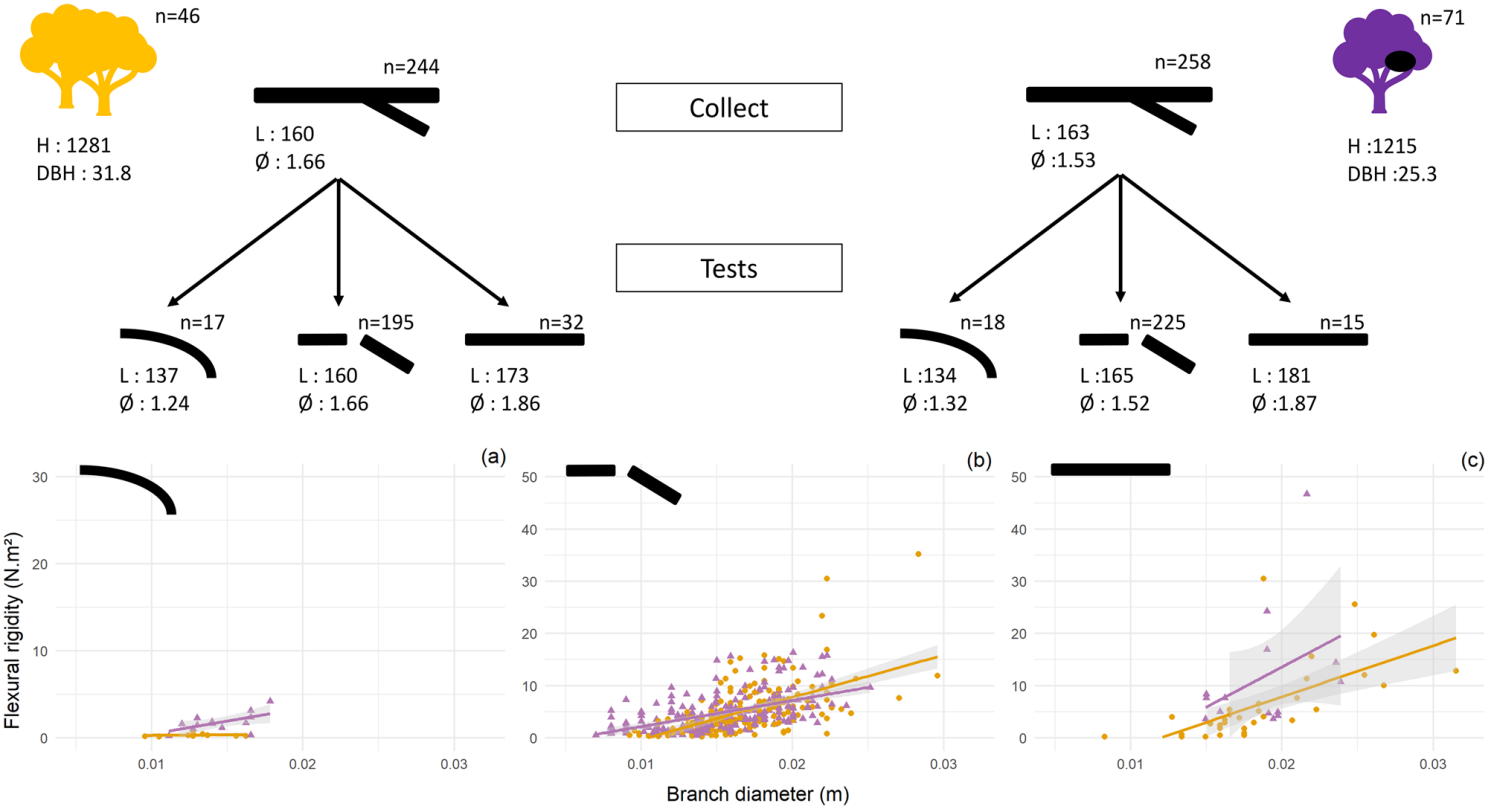
Linear regression of rigidity per branches diameter according to each branch response to the test: bending (**a**), breaking (**b**) and not breaking (**c**). *Orange represents abundant tree species not selected by chimpanzees and purple represents nesting tree species. H: height of the tree, DBH: diameter at breast height, L: branch length and Ø: branch diameter. All measurements are in cm.*

**Figure 4:**
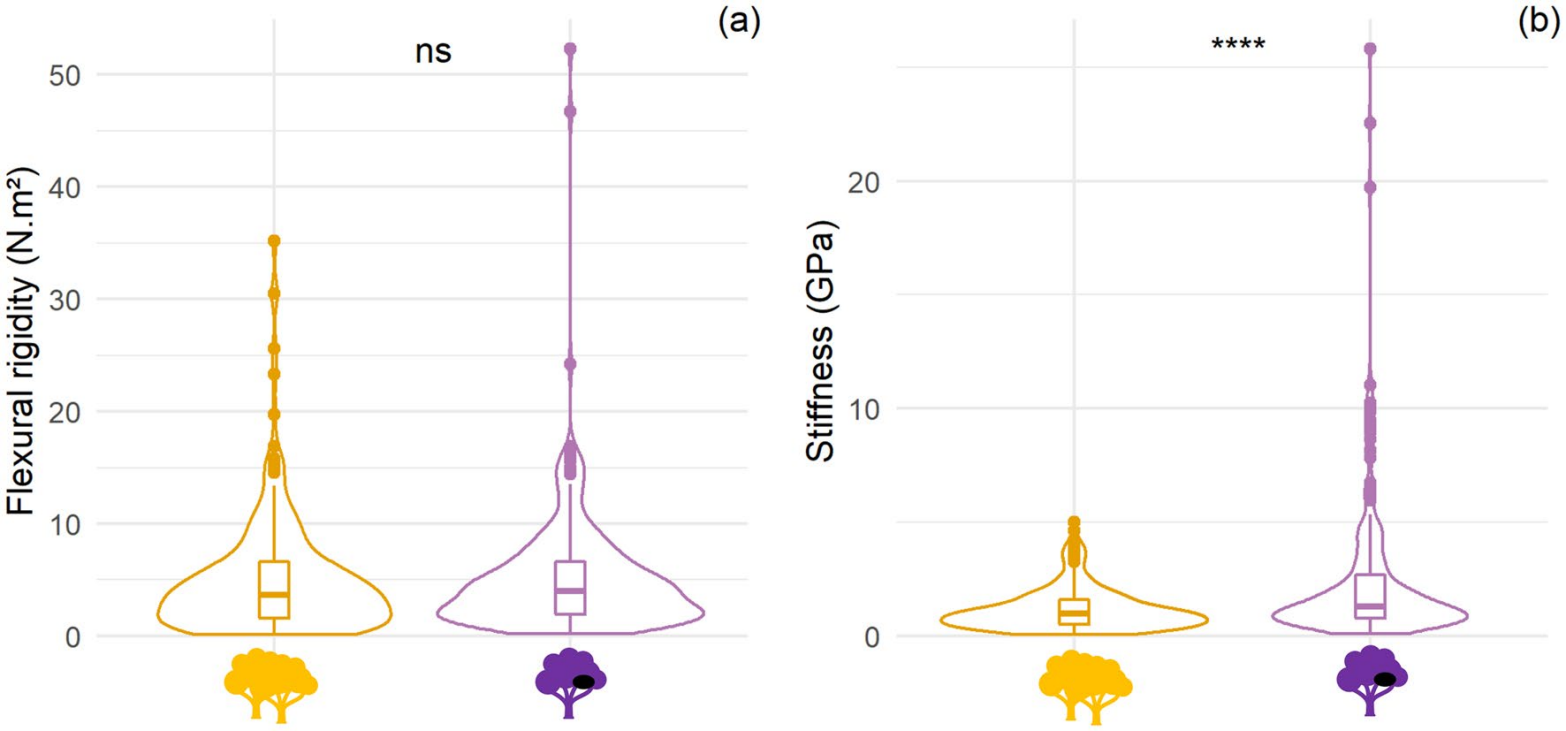
Violin boxplot of flexural rigidity values (**a**) and stiffness values (**b**). *Orange represents abundant tree species not selected by chimpanzees and purple represents nesting tree species.*

Branch reactions are significantly different between the two studied tree categories (χ^2^ = 7.94, *df* = 2, *p* value = 0.019) with branches ‘breaking’ more often in nesting tree species (87.21% vs. 79.92%) and more ‘not breaking’ for abundant tree species (13.11 vs. 5.81%). However, branch reactions to weight are associated with different branch diameter (anova, F_2,494_ = 25.2, *p* value < 0.001) and branch diameter is significantly smaller for nesting tree species (1.52 ± 0.39 vs. 1.66 ± 0.37 cm; t test, t = 3.91, *df* = 494.93, *p* value < 0.001). Rigidity of branch is not significantly different between nesting and abundant tree species (4.54 ± 5.57 vs. 4.36 ± 5.21 N m^2^; t test, t = − 0.38, *df* = 478.56, *p* value = 0.7059). There is an important intra and inter-species variability in flexural rigidity within the tree categories resulting in no significant difference between the two main groups (Fig. 3). However, we can observe a trend whereby small diameters are associated with stiffer branches in the case of nesting tree species compared with abundant tree species. The modulus of elasticity of the wood is significantly higher for branches from nesting tree species compared to abundant tree species (1.59 ± 3.21 vs. 0.88 ± 0.94 GPa; t test, t = -5.90, *df* = 337.44, *p* value < 0.0001) (Fig. 4).

### Chimpanzee nesting tree selectivity

**Figure 5:**
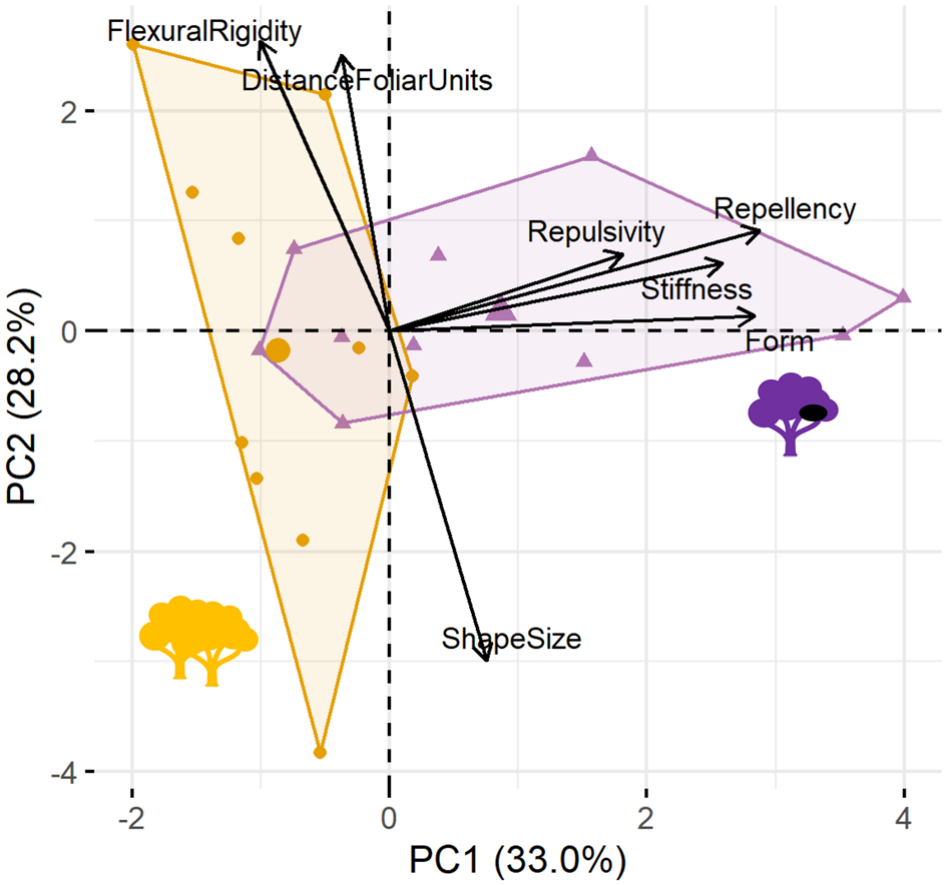
PCA of quantitative variables to quantify and visualize chimpanzee tree selectivity. *Orange represents abundant tree species not selected by chimpanzees and purple represents nesting tree species. DistanceFoliarUnits: mean distance between foliar units; NestingOccurence: number of nests recorded; PresenceHabitat: number of trees recorded in the habitat; Repellency: spatial repellent activity recorded in essential oil of the leaves, FlexuralRigidity: mean value of flexural rigidity; Stiffness: mean value of Young’s elastic modulus; Form: mean value characterizing the form of foliar unit.*

PCA analysis on all of the quantitative variables collected, reveals that the four PC explained 82.9% of the variance and the first PC is key in separating the two tree types (manova, F_1,18_ = 8.20, *p* value = 0.0103). The elasticity, the repellency, the number of trees in the habitat and the number of nesting occurrence per tree species mostly contribute to the PC1 and are strongly correlated to each other (Fig. 5).

However, the results from the conditional inference tree show that the only significant variable in separating the two tree types is the qualitative variable describing the shape-size foliar units (Asymptotic General Independence Test, *p* value = 0.002). Overall, all ten nesting tree species and one abundant tree species (*Celtis africana*) have foliar units with middle size and elongated shape. All nesting tree species also have a high foliar density (i.e. small distance between foliar units) and most of them (70%) have spatial repellent activity against mosquitoes (Table 1).

**Table 1:** Description of the mechanical and chemical properties of 20 tree species occurring in Sebitoli chimpanzee habitat.

Scientific name	Chemical	Biomechanical and morphological				Rank presence in habitat
	Spatial repellent	Foliar form	Foliar density (cm)	Branch flexural rigidity: EI (N m^2^)	Young’s elastic modulus: E (GPa)	
NESTING TREE SPECIES (> 1% nests)						
*Diospyros abyssinica* (Hiern) F.White (27.8%)	**Yes**	**Middle**	**2.17 ± 0.54**	3.20 ± 2.83	**3.00 ± 3.25**	**2nd**
*Strombosia schefflerii* Engl. (18.7%)	No	**Middle**	**3.79 ± 0.64**	**7.20 ± 9.33**	**4.52 ± 4.51**	**8th**
*Vepris nobilis* (Delile) Mziray (8.6%)	**Yes**	**Middle**	**2.97 ± 0.54**	2.95 ± 1.88	**5.80 ± 6.00**	29th
*Lepisanthes senegalensis* (Poir.) Leenh. (7.0%)	**Yes**	**Middle**	**3.28 ± 0.95**	**5.11 ± 4.66**	**2.39 ± 2.14**	23rd
*Turraeanthus africanus* (Welw. Ex C.DC.) Pellegr. (5.8%)	**Yes**	**Middle**	**2.36 ± 0.30**	**7.37 ± 4.38**	0.85 ± 0.33	60th
*Croton megalocarpus* Hutch. (4.4%)	**Yes**	**Middle**	**1.09 ± 0.25**	**5.88 ± 8.91**	1.30 ± 1.14	**7th**
*Celtis gomphophylla* Baker (3.2%)	No	**Middle**	**2.17 ± 0.32**	3.49 ± 3.50	1.01 ± 0.58	**5th**
*Olea welwitschii* (Knobl.) Gilg & G.Schellenb. (2.3%)	No	**Middle**	**2.71 ± 0.92**	**5.80 ± 3.62**	1.75 ± 0.90	50th
*Eucalyptus grandis* W.Hill ex Maiden (2.0%)	**Yes**	**Middle**	**1.61 ± 0.47**	5.09 ± 3.38	1.63 ± 0.95	**1st**
*Noronhia africana* (Knobl.) Hong-Wa & Besnard (1.8%)	**Yes**	**Middle**	**3.05 ± 0.71**	4.68 ± 3.25	1.30 ± 2.03	25th
ABUNDANT TREE SPECIES (< 1% nests and > 1% of trees in the habitat^31^)						
*Trilepisium madagascariense* DC. (0.6%)	No	Small	**2.91 ± 0.73**	2.02 ± 2.70	0.83 ± 0.97	**10th**
*Chrysophyllum albidum* G.Don (0.4%)	No	Small	**2.75 ± 0.05**	4.02 ± 2.93	0.93 ± 0.59	**20th**
*Uvariopsis congensis* Robyns & Ghesq. (0.3%)	**Yes**	Small	**2.26 ± 0.43**	**5.15 ± 3.90**	1.71 ± 0.89	**8th**
*Neoboutonia macrocalyx* Pax (0.3%)	No	Large	**3.08 ± 0.81**	**8.36 ± 7.23**	0.91 ± 0.52	**12th**
*Euadenia eminens* Hook.f. (0.3%)	No	Small	**3.22 ± 0.16**	4.24 ± 2.97	0.75 ± 0.46	**15th**
*Tabernaemontana pachysiphon* Stapf (0.1%)	No	Large	13.49 ± 4.56	4.11 ± 4.79	0.78 ± 0.66	**13th**
*Newtonia buchananii* (Baker) G.C.C.Gilbert & Boutique (0.1%)	No	Small	0.10 ± 0.05	2.93 ± 3.90	0.95 ± 0.62	**14th**
*Celtis africana* Burm.f. (0.1%)	**Yes**	**Middle**	**3.29 ± 0.73**	4.52 ± 4.77	1.59 ± 1.33	**16th**
*Carapa grandiflora* Sprague (0%)	**Yes**	Large	6.45 ± 1.01	**8.84 ± 7.90**	1.57 ± 1.09	**6th**
*Alangium chinense* (Lour.) Harms (0%)	No	Large	4.88 ± 1.03	**5.80 ± 4.55**	1.89 ± 1.18	**21st**

In bold, variables of potential value for chimpanzees. Spatial repellent: activity recorded in leaf essential oil; Foliar form: qualitative factor from the consensus shape and size of foliar unit; Branch rigidity: mean ± sd value of branch rigidity; Branch elasticity: mean ± sd value of branch elasticity; Foliar density: mean ± sd distance in cm between foliar units; Rank presence in habitat: rank of trees recorded in the Sebitoli chimpanzee habitat^31^.

## Discussion

Our results suggest that chimpanzees could choose tree species in which they nest according to some specific biomechanical and morphological conformations: relative smaller foliar units with ‘middle’ size and elongated shape, relative higher leaf density, smaller branch diameter that break more and branches with wood of a higher elastic modulus.

Consistent with a previous study^16^, nesting tree species have a smaller internodal distance (here a short distance between foliar units). This may introduce a greater number of structural points (i.e. intersection between the branch and the peduncle of the foliar unit), reducing exposure to branches protruding from the nest structure and increasing friction between branches interweaving, thus resulting in a greater comfort by more integrity and resilience in the woven structure^16^. Further research should be done to strengthen this theory of greater number of structural points. It is also possible that chimpanzees place additional leaves or twigs over hard branches to increase comfort^17^. Interestingly, chimpanzees have already proven that they can consider multiple physical properties when choosing materials in other behaviors^18^. In addition, previous experiments have indicated that chimpanzee choice considers size and quantity simultaneously^19^. It has been suggested that it is short internode distance combined with relatively small leaves that is selected for nesting, creating better insulation and thus provide thermoregulatory benefits^16^. These data also highlight the importance of another morphological criterion in the selectivity of Sebitoli chimpanzees: the unit size of leaves. According to some studies, chimpanzees and bonobos choose small leaves for their nest (^16^ and^5^ respectively). Considering the mean value for foliar unit size in our data set, it is also true that Sebitoli chimpanzees choose in general smaller leaves than those available in the habitat but not the smallest ones, i.e. small leaflets of *Newtonia buchananii*. First, it should be noted that these notions of small and medium foliar size are relative to the habitat in which the study is conducted. On the other hand, in the case of Sebitoli, there seems to be a limit reached where smaller leaves no longer mean more comfortable nests. Indeed, some tree species present in Sebitoli have very small leaves and foliar density similar to that of medium leaves, which would increase exposure to branches and air, leading to a less comfortable structure. Medium leaves associated with a high density of foliar units could offer better thermal and physical comfort^20^. All nesting tree species have a ‘middle’ size foliar unit from what is available in their habitat (‘small’, ‘middle’ and ‘large’ types). Moreover, these selected leaves have also an elongated shape. The combined morphological criterion of foliar unit form furthermore emerges as the most discriminating feature to explain nest tree selectivity. This study highlights the capacity of chimpanzees to take size and shape into account in their decision making, an ability already demonstrated in their grasping behavior^21^ similar to shape considered by orangutans^22^. The tendency of chimpanzees to choose elongated foliar units could be the by-product of choosing medium size foliar units as our data set does not offer other conformations, e.g. the “medium-sized, heart-shaped foliar unit” is not present. It will be interesting to study different chimpanzee communities with others combinations of foliar unit forms to fully understand nesting selectivity. Chimpanzee could choose tree species that look alike and disfavored foliar units with specific shapes. Further morphometrics analysis would be needed to understand the implications behind these preliminary results on the foliar unit form. Alternatively, studying communities living in the same environment with similar conformations of leaf units could be interesting if they favor different shapes, as this could indicate a potential cultural trait.

We found on average, more broken branches with smaller diameter for the nesting tree species. Tree branches generally exhibit greenstick fractures (i.e. they break halfway across before splitting^23,24^) or buckling. These failure modes keep the branches attached, so within the nest structure they could promote the strength and compliance of the structure compared to completely snapped branches. The density of the wood species seems to play a role where lighter wood with low transverse compressive strength buckle more and dense wood with high transverse compressive strength fracture more^23^. As for the branches not breaking, the weight applied to the branches by our design was not sufficient to trigger a fracture or buckling. Sebitoli chimpanzees might prefer denser wood with high transverse compressive strength for nesting, but further studies on wood density and anatomy are needed with measurements such as tensile strength, torque, compliance and brittleness. Contrary to our expectations, we do not find a clear impact of the rigidity value in the nest tree selection process as reported in the study of Samson and Hunt^16^. This may be related to the specific environment of the Toro-Semliki chimpanzees, which show a strong preference for one particular rigid tree, *Cynometra alexandri,* which accounts for over 70% of nesting events*.* In comparison, in the Sebitoli community, 10 species account for 80% of nesting events. The branches studied show too much variability in their flexural rigidity values, mainly because of the wide range of branch diameters involved (branch diameter contributes to the calculation by a factor of 4), which produces non-significant results by species and tree type. Abundant tree species have the largest branch diameters, which appears to have a positive influence on the overall flexural rigidity for this category. Interestingly, for smaller diameters, branches of nesting tree species tend to have higher flexural rigidity than those of abundant tree species. On the other hand, we found significant differences in flexural modulus of elasticity of the branches between the two types of trees, where nesting tree species have branches with higher elastic modulus. In this regard, chimpanzees appeared to choose branches with stiffer wood that do not deform easily. Interestingly, chimpanzees probably need both compliant and rigid branches for their nests and that they could utilize the branches’ diameter to obtain these two characteristics more easily and with lower diameters in tree species with stiffer wood. Indeed, orangutans^15^ and birds^14^ choose more compliant branches for the nest center and stiffer branches for the nest edges. Consideration of the location of bent branches in the nest could provide information for further investigation. The position of the different branches will be taken into account during future research by manually dissecting the nest and/or by reconstructing the structure of the nest with a 3D scanner.

Taking into account the two major frameworks explaining tree species selectivity^9,16^, we also consider the spatial repellency properties of leaves. Even if most nesting trees are indeed repellent in this study -unlike not selected tree species-, it does not fully explain Sebitoli chimpanzees’ choice as well as the foliar unit form. Chimpanzees would primarily choose their trees according to comfort, and fortunately enough most tree species morphologically and mechanically comfortable are also chemically comfortable in Sebitoli. In addition, other biomechanical and morphological properties not measured in this study may also play a role in selectivity. For example, softness of leaf units could play a role, and/or certain tree architectures could be favored, such as the inverted tripod branching patterns of trees with "lollipop" morphology in the Toro-Semliki community^16^.

Finally, in our study design, we have not considered less abundant tree species except if they are chosen by chimpanzees. Regardless, most trees selected for nesting are quite abundant (1st, 2nd, 5th 7th and 8th of the tree present in Sebitoli habitat). So, the natural occurrence of tree species probably plays a part in the selectivity of tree species. It is possible that some tree species met all criteria measured in this study and are not chosen because of their rarity. One such notable exception is *Celtis africana* which is abundant, characterized by all the characteristics shown to be selected in this study, and yet is not chosen for nesting. Chimpanzees and elephants eat the leaves^25,26^, so some trees are quite damaged and/or leafless. However, their good number make it theoretically possible to find trees good enough to make nest. Chimpanzees have been observed to avoid fruiting tree species^27^ because of possible disturbance by sympatric species (e.g., bats^28^ and gorillas^29^). This could extend to leafing tree species, especially those that some animals (e.g., elephants) feed on at night and that could disturb them. Interestingly, when collecting their leaves, we have learned that local people find the odor quite strong and foul smell. It is possible that chimpanzees might also dislike the odor, but this remains a difficult theory to prove.

In conclusion, from a limited number of variables tested, we demonstrated that the particular community of chimpanzees from Sebitoli mainly consider nest tree species according to the morphological and mechanical comfort they offer. In this habitat, all tree species mechanically and morphologically comfortable were also chemically comfortable. We were unable to determine if the tree species were selected for their repellent properties or if they were coincidentally repellent. Studying more tree species may help us understand the proportion of mechanically and/or chemically comfortable tree species actually available. We believe that such a study will confirm that chimpanzees do prefer tree species that are comfortable in both aspects. Beyond the fact that the chimpanzees improved their sleep quality and cognition by sleeping in a nest^5^, these results show that these effects could be reinforced by choosing tree species that provide the most comfort. In addition, finding repellent tree species may represent bio-inspired solutions for humans and highlight the importance of protecting these endemic trees as useful resources for humans and non-human health. Further research on additional criteria and these two properties might be relevant in different social groups of a same population to understand tree selectivity and whether there are differences linked to ecological factors (type of tree species present in their habitat) or that could be described as cultural (not explain by environment or genetics).

## Methods

### Study site—nesting behavior

The study took place in the Sebitoli area located at the extreme North part of the Kibale National Park in Western Uganda (795 km^2^, 0°13′–0°41′ N and 30°19′–30°32′ E1^30^). Between June 2017 and June 2019, the Sebitoli Chimpanzee Project team followed wild chimpanzees (*Pan troglodytes schweinfurthii*) daily and collected nesting data, including the nesting occurrence per tree species presented in a previous study^10^. A tree census in the same area, already showed the abundance of each tree species in 79 plots representative of the habitat^31^. To understand the tree species selectivity that chimpanzees exhibit for their nesting behavior, we compared ten tree species most used for “nesting” (regardless of their abundance in the habitat) to ten tree species “abundant” in their environment (> 1% of trees recorded out of 95 tree species identified) yet rarely used by chimpanzees (< 1% of nests), that excludes the 3rd and 4th tree species which are *Funtumia africana* (Benth.) Stapf and *Markhamia lutea* (Benth.) K.Schum. All tree species has already been collected in 2006 and determined at the herbarium of the Laboratoire de Phanérogamie at the Muséum National d’Histoire Naturelle (Paris, France) where voucher specimens have been deposited^25^. The spatial repellent property of each of those 20 species against the African mosquito *Anopheles gambiae* (detailed description of the materials and methods available in^10^) was considered as positive when there were significantly more repellent than the control for at least one concentration. Spatial repellent activity was reported in this study as the bioassay results for the concentration with the maximum effect.

### Leaves and branches collection and characterization

During the two surveys conducted in dry and rainy seasons of 2019 and 2021, we collected 5 branches with length and diameter similar to bent but not broken branches that were initially found in nests, from trees belonging to the 20 tree species studied so 25 branches per tree species. For each tree, we measured its diameter at breast height (DBH) using a tape measure and its height (H) using a rangefinder. We measured the area of simple leaves and the mean area of leaflets for complex leaves and obtain the area of foliar units. We chose to use foliar units, e.g. single leaves or folioles of complex leaves, assuming that chimpanzees do not distinguish this botanical difference. As much as possible, we selected the most complete foliar unit with the most representative size of the tree under study, not necessarily from the branches measured for rigidity. We measured ten linear distances between foliar units in around five trees per species, equivalent to the foliar density^16^, and when foliar units were no longer present, we used the scar they leave on the branch as a marker (Table S1).

By using the geometric morphometric approach, we aimed to make shape comparisons in a quantitative way^32^. In order to characterize the two-dimensional shape of foliar units, anatomical landmarks were set, one on each side of the base with the petiole (Figure S2). Then, we placed 98 evenly spaced sliding semi-landmarks around the margins for each foliar unit^33^ using tpsDig, version 2.05^34^ (Table S3).

To compare the tree species biomechanical properties, we placed branches from the 20 species according to an experimental design at low cost and field-buildable (Figure S4) during three surveys conducted in 2018, 2019 and 2021. We placed a 50 cm-length branch on a horizontal support. A bucket (weighing 0.750 kg) was attached to the branch 20 cm from the edge of the support. We successively added one liter of water at a time up to 10 L then two liters up to 24 L. The distance and angle between the branch and a horizontal reference element placed on the support were measured for each weight. We obtained relativized measurements by taking into account the distance and angle at 0 L. The branch could either buckle by having a deflection angle greater than 90° with no visible cracks, break (snap or fracture) by having visible cracks, or not break^23^. The material properties were obtained through classical cantilevered beam theory: the stiffness of a material with the standard Young’s elastic modulus (E) refers to a mechanical property that is the ability of being stretch without being irreversibly deformed, it is computed as the ratio of stress to strain when bending. The flexural rigidity (EI) refers to a structural property that is the material resistance to deformation. One is derived from the other by the moment of inertia (I) which take into account the shape/size of the material that is the branch diameter (BD) [m] as:

$$I = \frac{\pi \times {(BD)}^{4}}{64}$$

To obtain EI:

$$EI=\frac{\Delta F}{\Delta DD}\times \frac{{L}^{3}}{3}$$

where we used the slope of the initial linear region of the plot between the force (F) applied to the branch (weight [L] * gravitational acceleration of earth [m s^2^]) and the deflection distance (DD) [m] (Table S5). We also integrated the length between the fixed end to the point of load (L) where the bucket hangs, L = 0.20 m.

This approach was done for branches for which the breaking point was more than 8 L to have enough points to obtain the initial linear slope. For the others branches, we used the maximum deflection point corresponding to the maximum deflection distance with the maximum weight. The flexural rigidity and stiffness obtained per branches are available in Table S6.

The cantilever beam theory is a standard method in the literature for tree branches, but it is based on different assumptions that are not always met by some branches; however, this should not significantly affect the results at the study level. Indeed, a plant material is not an ideal solid nor and ideal fluid and an exact value for a parameter may be difficult to obtain^35^. For example, the flexural rigidity and stiffness are measured for the whole beam and therefore assuming that it is homogeneous and isotropic, even though the tree branches are not^36^. Also, the beam is assumed to be initially straight and without imperfections, which was not the case for all the branches in this study. The second moment of area (I) using diameter assumes a circular cross section that remains the same along the length of a branch. However, the diameter was not strictly the same along the entire length of the branches used. Furthermore, biological materials acclimatize and can change their material properties over time and growth^37^. Our method of collecting and characterizing leaves and branches is therefore as optimal as possible according to field conditions and available data.

### Data analysis

All statistical analyses were performed with R software, version 3.6.0^38^ and aimed to compare nesting and abundant tree species. For the distance between foliar units and their area, we used an independent Student’s t-test to compare means. To analyze the foliar unit shape, we performed a geometric morphometric analysis with the ‘Morpho’ package version 2.9^39^, the ‘geomorph’ package 4.0.3^40^ and the ‘Momocs’ package version 1.4.0^41^. Sliding process as well as a Procrustes alignment were performed to enable Principal Component Analysis (PCA) on the aligned coordinates of multiple specimens to quantify and visualize shape variation (residuals after translation, scaling and rotation) in low-dimensional space^33,42^. If chimpanzees could be sensitive to shape, we expected that they also could be sensitive to size of foliar units, therefore we explored allometry in our dataset. Quantification and visualization of allometry could help us to identify if the different shapes observed are associated to particular size. We obtained normalized shape scores from a ‘Common Allometric Component’ (CAC) that we visualized against log-transformed size^43^. Finally, the average species form was obtained with a consensus of all the foliar units. The score of the first principal component (PC) provided a quantitative value to characterize the “form” (i.e. shape and size combined)^44^ of the average foliar units per species.

When investigating branch rigidity, we divided our analysis according to each type of test reaction (buckling, breaking or not breaking). Then, to understand whether there was a difference between abundant and nesting trees, we performed a linear regression of branch flexural rigidity with branch diameter in interaction with tree type.

To gain a clear picture of the chimpanzee selectivity, we combine all our quantitative data (number of nesting occurrences, number of trees in the habitat, mean distance between foliar units, mean foliar unit form, mean rigidity, mean elasticity and repellency) per tree species in a PCA with the package ‘FactoMineR’ version 2.4^45^ and ‘factoextra’ version 1.0.7^46^. Finally, we implemented a conditional inference tree to extract the most discriminant variables in our data set that could give insight into chimpanzee selectivity in terms of nesting trees. We described each species by the quantitative data (number of trees in the habitat, mean distance between foliar units, mean flexural rigidity and mean stiffness) and the qualitative data (repellency ‘yes/no’ and mean foliar unit form divided in 3 categories ‘small’ elongated foliar unit, ‘middle’ size and elongated foliar unit and ‘large’ size with elongated or hearted foliar unit) to divide our dataset according to trees type with the package ‘party’ version 1.3–10^47^.

### Ethical guidelines

The permission to conduct this research and the field experiments on plants were obtained and conducted in the context of the Memorandum of Understanding MNHN/UWA/Makerere University SJ 445–12 following the guidelines of the Uganda Wildlife Authority (UWA).

### Additional information

The Great Ape Conservation Project, the Fondation Ensemble, the Fondation pour la Nature et l’Homme, the Fondation Prince Albert II and the Fonds Français pour l’Environnement Mondial provided financial support for the research conducted at Sebitoli. We deeply thank La Phocéenne de Cosmétique and the Association Nationale de la Recherche et de la Technologie for providing funds for the PhD scholarship of Camille Lacroux.

### Supplementary Information

Supplementary Information 1.

Supplementary Information 2.

Supplementary Information 3.

Supplementary Information 4.

Supplementary Information 5.

Supplementary Information 6.

## Data Availability

All data generated or analyzed during this study are included in this published article (and its Supporting Information files).
