# Supplementary figures and images for: Chimpanzees select comfortable nesting tree species
Source: Sci Rep. 2023 Oct 7;13:16943. doi: 10.1038/s41598-023-44192-6 (PMC10560204; doi:10.1038/s41598-023-44192-6)

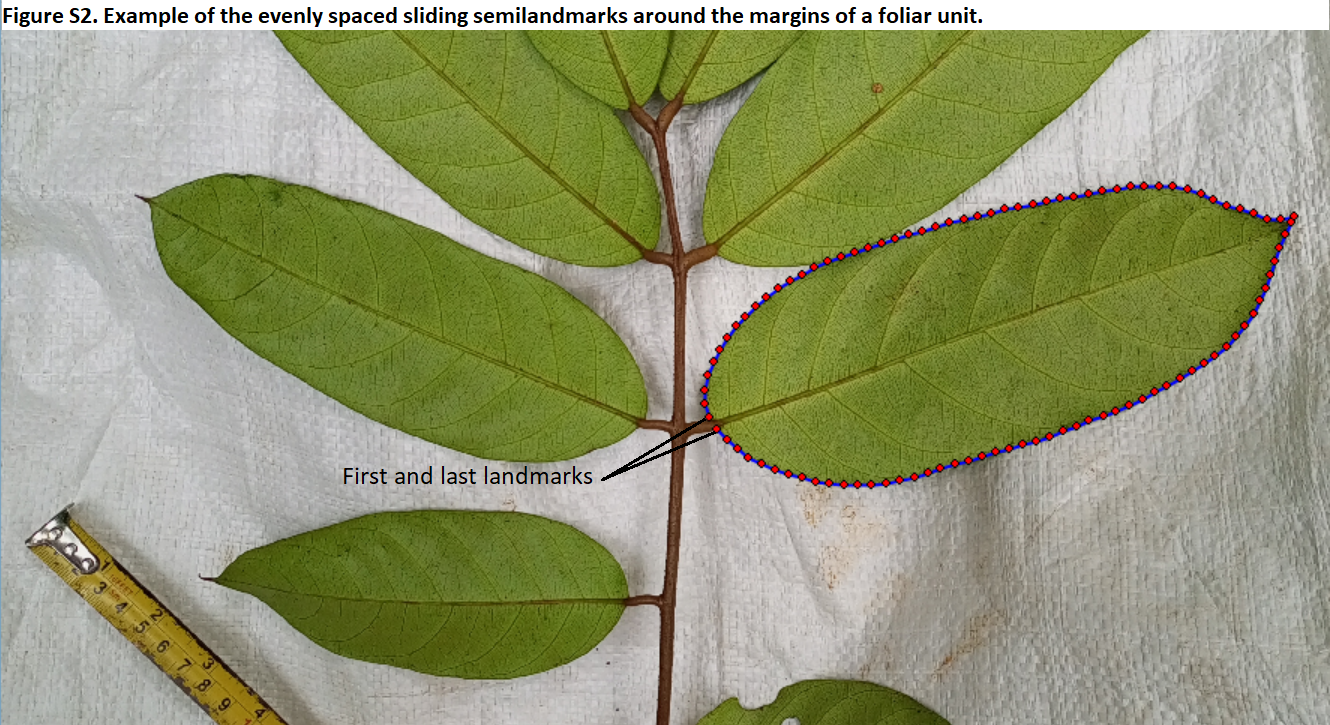

Supplement: Supplementary file 2 — Supplementary Information 2. [file 41598_2023_44192_MOESM2_ESM.tiff]

**Figure S4. Schema on how branch rigidity was measured.**

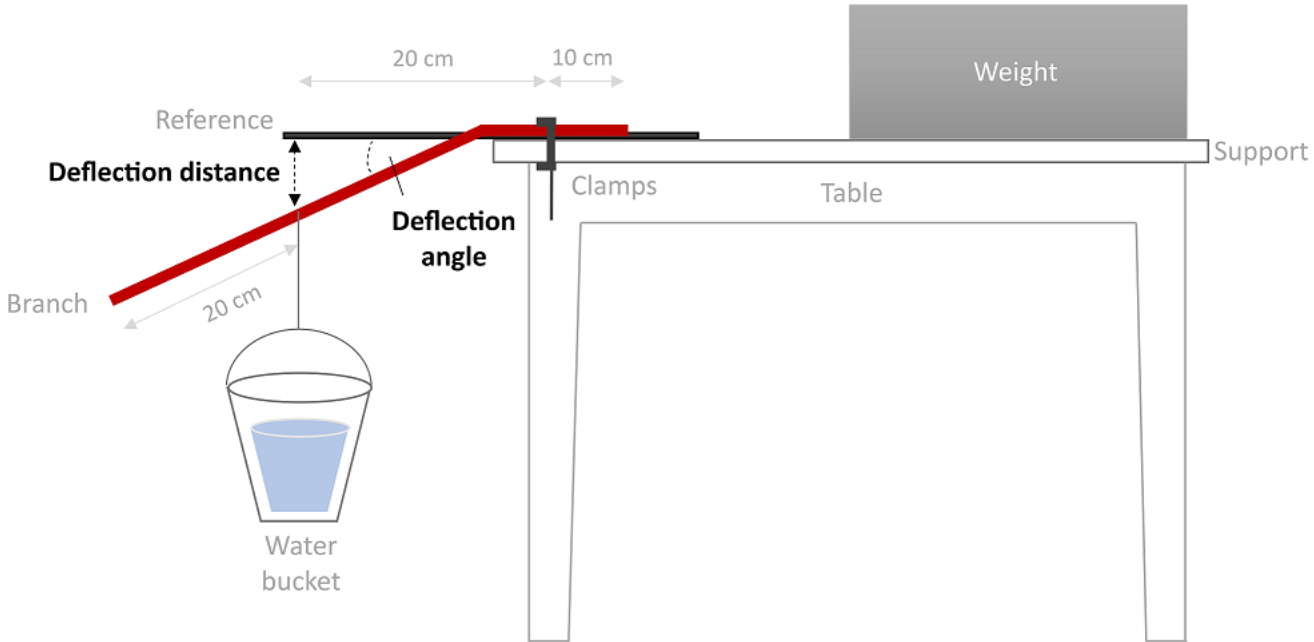

Supplement: Supplementary file 4 — Supplementary Information 4. [file 41598_2023_44192_MOESM4_ESM.pdf]
