# Supplementary material for: Chimpanzees select comfortable nesting tree species
Source: Sci Rep. 2023 Oct 7;13:16943. doi: 10.1038/s41598-023-44192-6 (PMC10560204; doi:10.1038/s41598-023-44192-6)
Supplement: Supplementary file 3 — Supplementary Information 3. [file 41598_2023_44192_MOESM3_ESM.pdf]

Table S3. Data on the morphometry of the foliar units.

LM=0

CURVES=1

POINTS=100

333.00000 709.00000

343.00000 705.00000

353.00000 701.00000

362.00000 696.00000

371.00000 691.00000

380.00000 686.00000

389.00000 681.00000

398.00000 675.00000

407.00000 670.00000

416.00000 665.00000

426.00000 660.00000

435.00000 654.00000

444.00000 649.00000

453.00000 643.00000

461.00000 638.00000

471.00000 634.00000

481.00000 631.00000

491.00000 627.00000

501.00000 623.00000

511.00000 620.00000

521.00000 619.00000

532.00000 617.00000

542.00000 615.00000

552.00000 615.00000

563.00000 614.00000

573.00000 614.00000

584.00000 613.00000

594.00000 614.00000

605.00000 615.00000

615.00000 616.00000

626.00000 618.00000

636.00000 620.00000

646.00000 623.00000

656.00000 626.00000

666.00000 629.00000

676.00000 632.00000

686.00000 635.00000

696.00000 638.00000

706.00000 642.00000

715.00000 647.00000

724.00000 652.00000

733.00000 658.00000

742.00000 663.00000

751.00000 668.00000

760.00000 674.00000

769.00000 679.00000

778.00000 685.00000

787.00000 690.00000

796.00000 695.00000

805.00000 701.00000

811.00000 710.00000

805.00000 716.00000

795.00000 720.00000

785.00000 724.00000

775.00000 727.00000

765.00000 730.00000

755.00000 734.00000

746.00000 737.00000

736.00000 741.00000

726.00000 744.00000

716.00000 748.00000

706.00000 751.00000

696.00000 755.00000

686.00000 758.00000

676.00000 762.00000

666.00000 765.00000

656.00000 768.00000

646.00000 772.00000

637.00000 775.00000

627.00000 779.00000

616.00000 782.00000

607.00000 785.00000

597.00000 789.00000

587.00000 791.00000

576.00000 792.00000

566.00000 793.00000

555.00000 794.00000

545.00000 794.00000

534.00000 793.00000

524.00000 793.00000

513.00000 792.00000

503.00000 791.00000

492.00000 789.00000

482.00000 788.00000

472.00000 786.00000

461.00000 784.00000

451.00000 783.00000

441.00000 780.00000

431.00000 777.00000

421.00000 774.00000

411.00000 770.00000

401.00000 766.00000

393.00000 760.00000

384.00000 754.00000

375.00000 749.00000

366.00000 743.00000

358.00000 736.00000

351.00000 729.00000

343.00000 721.00000

336.00000 714.00000

IMAGE=20190909LEUA01.jpg

ID=0

SCALE=0.020252

LM=0

CURVES=1

POINTS=100

424.00000 253.00000

424.00000 265.00000

425.00000 277.00000

426.00000 289.00000

426.00000 301.00000

425.00000 313.00000

423.00000 325.00000

421.00000 337.00000

424.00000 348.00000

423.00000 360.00000

421.00000 372.00000

424.00000 384.00000

424.00000 396.00000

422.00000 407.00000

420.00000 419.00000

418.00000 431.00000

416.00000 443.00000

414.00000 455.00000

412.00000 467.00000

410.00000 479.00000

408.00000 490.00000

406.00000 502.00000

404.00000 514.00000

402.00000 526.00000

400.00000 538.00000

394.00000 548.00000

387.00000 558.00000

381.00000 568.00000

375.00000 579.00000

370.00000 590.00000

365.00000 601.00000

359.00000 611.00000

353.00000 621.00000

347.00000 632.00000

342.00000 642.00000

334.00000 651.00000

324.00000 658.00000

315.00000 666.00000

309.00000 676.00000

299.00000 683.00000

290.00000 691.00000

282.00000 700.00000

274.00000 708.00000

264.00000 716.00000

255.00000 724.00000

245.00000 730.00000

235.00000 736.00000

224.00000 741.00000

213.00000 747.00000

205.00000 756.00000

197.00000 764.00000

192.00000 762.00000

193.00000 750.00000

194.00000 738.00000

195.00000 726.00000

195.00000 714.00000

196.00000 702.00000

196.00000 690.00000

197.00000 678.00000

197.00000 666.00000

194.00000 655.00000

193.00000 643.00000

193.00000 631.00000

196.00000 619.00000

199.00000 607.00000

200.00000 595.00000

200.00000 583.00000

202.00000 572.00000

203.00000 560.00000

207.00000 548.00000

211.00000 537.00000

215.00000 526.00000

219.00000 514.00000

226.00000 504.00000

232.00000 494.00000

239.00000 484.00000

244.00000 474.00000

252.00000 465.00000

261.00000 457.00000

269.00000 448.00000

276.00000 438.00000

282.00000 427.00000

289.00000 417.00000

296.00000 408.00000

302.00000 398.00000

307.00000 387.00000

311.00000 375.00000

318.00000 365.00000

326.00000 357.00000

334.00000 348.00000

342.00000 339.00000

349.00000 329.00000

357.00000 320.00000

366.00000 312.00000

374.00000 304.00000

383.00000 296.00000

394.00000 290.00000

401.00000 281.00000

409.00000 272.00000

418.00000 264.00000

IMAGE=20190909LEUA02.jpg

ID=1

SCALE=0.020252

LM=0

CURVES=1

POINTS=100

98.00000 28.00000

116.00000 37.00000

137.00000 39.00000

156.00000 46.00000

175.00000 54.00000

192.00000 66.00000

211.00000 73.00000

229.00000 83.00000

248.00000 90.00000

268.00000 96.00000

288.00000 99.00000

307.00000 107.00000

322.00000 120.00000

336.00000 136.00000

353.00000 148.00000

369.00000 160.00000

385.00000 173.00000

402.00000 185.00000

418.00000 197.00000

431.00000 213.00000

443.00000 230.00000

457.00000 245.00000

469.00000 261.00000

478.00000 280.00000

488.00000 298.00000

498.00000 316.00000

505.00000 335.00000

512.00000 354.00000

519.00000 374.00000

527.00000 393.00000

532.00000 412.00000

535.00000 433.00000

538.00000 453.00000

539.00000 473.00000

539.00000 494.00000

540.00000 514.00000

541.00000 535.00000

539.00000 555.00000

534.00000 575.00000

528.00000 595.00000

522.00000 614.00000

516.00000 634.00000

510.00000 654.00000

504.00000 673.00000

499.00000 693.00000

494.00000 713.00000

487.00000 732.00000

477.00000 750.00000

465.00000 767.00000

456.00000 785.00000

436.00000 788.00000

416.00000 784.00000

396.00000 780.00000

376.00000 776.00000

356.00000 774.00000

336.00000 767.00000

318.00000 758.00000

301.00000 747.00000

283.00000 737.00000

266.00000 726.00000

248.00000 715.00000

230.00000 705.00000

212.00000 696.00000

194.00000 686.00000

178.00000 674.00000

162.00000 660.00000

149.00000 645.00000

134.00000 630.00000

120.00000 616.00000

106.00000 601.00000

95.00000 583.00000

86.00000 565.00000

70.00000 551.00000

61.00000 533.00000

53.00000 514.00000

44.00000 496.00000

36.00000 477.00000

31.00000 457.00000

26.00000 437.00000

21.00000 417.00000

17.00000 397.00000

14.00000 377.00000

11.00000 356.00000

9.00000 336.00000

6.00000 316.00000

5.00000 295.00000

5.00000 275.00000

6.00000 254.00000

7.00000 234.00000

12.00000 214.00000

17.00000 194.00000

20.00000 174.00000

23.00000 153.00000

33.00000 135.00000

51.00000 125.00000

65.00000 110.00000

75.00000 93.00000

77.00000 72.00000

74.00000 52.00000

69.00000 32.00000

IMAGE=20190911CNP01.jpg

ID=2

SCALE=0.038093

LM=0

CURVES=1

POINTS=100

576.00000 675.00000

558.00000 671.00000

540.00000 671.00000

523.00000 666.00000

505.00000 662.00000

488.00000 656.00000

470.00000 652.00000

453.00000 645.00000

436.00000 639.00000

420.00000 631.00000

404.00000 623.00000

387.00000 615.00000

372.00000 606.00000

356.00000 597.00000

341.00000 586.00000

328.00000 574.00000

313.00000 563.00000

300.00000 551.00000

286.00000 539.00000

274.00000 525.00000

262.00000 512.00000

250.00000 498.00000

240.00000 482.00000

232.00000 467.00000

219.00000 454.00000

207.00000 440.00000

195.00000 426.00000

187.00000 410.00000

180.00000 393.00000

171.00000 378.00000

161.00000 362.00000

152.00000 347.00000

143.00000 331.00000

134.00000 315.00000

126.00000 299.00000

122.00000 281.00000

114.00000 265.00000

106.00000 248.00000

99.00000 232.00000

95.00000 214.00000

91.00000 196.00000

87.00000 178.00000

83.00000 161.00000

80.00000 143.00000

78.00000 125.00000

79.00000 107.00000

81.00000 89.00000

82.00000 70.00000

88.00000 53.00000

100.00000 40.00000

114.00000 28.00000

131.00000 21.00000

149.00000 17.00000

167.00000 19.00000

185.00000 22.00000

202.00000 27.00000

220.00000 32.00000

236.00000 39.00000

252.00000 47.00000

269.00000 55.00000

284.00000 65.00000

298.00000 77.00000

314.00000 86.00000

329.00000 96.00000

343.00000 107.00000

358.00000 117.00000

373.00000 128.00000

387.00000 139.00000

403.00000 149.00000

414.00000 163.00000

425.00000 177.00000

436.00000 191.00000

448.00000 205.00000

459.00000 220.00000

471.00000 234.00000

481.00000 249.00000

492.00000 263.00000

502.00000 278.00000

513.00000 293.00000

522.00000 308.00000

530.00000 325.00000

538.00000 341.00000

547.00000 357.00000

556.00000 373.00000

562.00000 390.00000

568.00000 407.00000

574.00000 424.00000

582.00000 441.00000

586.00000 458.00000

591.00000 476.00000

596.00000 493.00000

597.00000 511.00000

599.00000 529.00000

600.00000 547.00000

597.00000 565.00000

600.00000 583.00000

598.00000 601.00000

595.00000 619.00000

593.00000 637.00000

591.00000 655.00000

IMAGE=20190911CNP02.jpg

ID=3

SCALE=0.031055

LM=0

CURVES=1

POINTS=100

571.00000 831.00000

548.00000 827.00000

525.00000 829.00000

503.00000 825.00000

480.00000 821.00000

457.00000 818.00000

434.00000 814.00000

412.00000 807.00000

390.00000 802.00000

367.00000 797.00000

345.00000 793.00000

323.00000 786.00000

302.00000 775.00000

282.00000 764.00000

263.00000 752.00000

243.00000 739.00000

226.00000 724.00000

211.00000 707.00000

195.00000 689.00000

179.00000 673.00000

162.00000 657.00000

149.00000 638.00000

136.00000 620.00000

122.00000 601.00000

109.00000 582.00000

97.00000 562.00000

86.00000 542.00000

76.00000 521.00000

65.00000 501.00000

55.00000 480.00000

45.00000 459.00000

37.00000 438.00000

29.00000 416.00000

18.00000 396.00000

17.00000 373.00000

13.00000 350.00000

6.00000 328.00000

7.00000 305.00000

9.00000 282.00000

7.00000 259.00000

10.00000 236.00000

4.00000 214.00000

3.00000 191.00000

12.00000 170.00000

19.00000 148.00000

23.00000 125.00000

27.00000 102.00000

38.00000 82.00000

47.00000 61.00000

53.00000 39.00000

51.00000 16.00000

62.00000 15.00000

81.00000 26.00000

104.00000 27.00000

127.00000 27.00000

150.00000 28.00000

173.00000 32.00000

196.00000 34.00000

218.00000 41.00000

238.00000 52.00000

257.00000 66.00000

277.00000 76.00000

299.00000 85.00000

320.00000 94.00000

340.00000 105.00000

359.00000 119.00000

377.00000 133.00000

395.00000 147.00000

413.00000 162.00000

430.00000 178.00000

445.00000 195.00000

461.00000 212.00000

475.00000 230.00000

488.00000 249.00000

501.00000 268.00000

513.00000 288.00000

525.00000 307.00000

537.00000 327.00000

548.00000 347.00000

556.00000 369.00000

566.00000 390.00000

576.00000 410.00000

581.00000 433.00000

591.00000 454.00000

600.00000 475.00000

607.00000 497.00000

613.00000 519.00000

616.00000 542.00000

618.00000 565.00000

618.00000 588.00000

620.00000 611.00000

622.00000 634.00000

619.00000 657.00000

616.00000 680.00000

612.00000 702.00000

608.00000 725.00000

600.00000 747.00000

595.00000 769.00000

587.00000 791.00000

587.00000 814.00000

IMAGE=20190912LCNP01.jpg

ID=4

SCALE=0.031066

LM=0

CURVES=1

POINTS=100

90.00000 20.00000

105.00000 34.00000

124.00000 42.00000

143.00000 49.00000

161.00000 58.00000

179.00000 68.00000

195.00000 81.00000

211.00000 94.00000

227.00000 106.00000

245.00000 117.00000

260.00000 130.00000

276.00000 144.00000

290.00000 158.00000

304.00000 174.00000

321.00000 184.00000

339.00000 195.00000

353.00000 210.00000

363.00000 228.00000

374.00000 245.00000

387.00000 261.00000

400.00000 277.00000

411.00000 294.00000

423.00000 311.00000

435.00000 327.00000

447.00000 344.00000

459.00000 360.00000

471.00000 377.00000

483.00000 393.00000

497.00000 408.00000

507.00000 426.00000

515.00000 445.00000

525.00000 463.00000

538.00000 478.00000

550.00000 495.00000

561.00000 512.00000

570.00000 531.00000

580.00000 549.00000

589.00000 567.00000

597.00000 586.00000

603.00000 605.00000

608.00000 625.00000

611.00000 645.00000

613.00000 666.00000

615.00000 686.00000

616.00000 707.00000

617.00000 727.00000

614.00000 747.00000

607.00000 766.00000

603.00000 786.00000

601.00000 814.00000

594.00000 808.00000

578.00000 797.00000

558.00000 796.00000

538.00000 794.00000

518.00000 787.00000

499.00000 780.00000

481.00000 771.00000

462.00000 763.00000

443.00000 755.00000

425.00000 745.00000

409.00000 732.00000

393.00000 720.00000

377.00000 707.00000

362.00000 692.00000

347.00000 679.00000

332.00000 665.00000

320.00000 649.00000

307.00000 633.00000

294.00000 617.00000

280.00000 602.00000

270.00000 584.00000

260.00000 566.00000

248.00000 549.00000

236.00000 533.00000

224.00000 516.00000

215.00000 498.00000

205.00000 480.00000

194.00000 463.00000

182.00000 446.00000

169.00000 430.00000

157.00000 414.00000

147.00000 396.00000

139.00000 377.00000

132.00000 358.00000

126.00000 338.00000

119.00000 319.00000

113.00000 300.00000

106.00000 280.00000

100.00000 261.00000

97.00000 240.00000

95.00000 220.00000

92.00000 200.00000

91.00000 179.00000

89.00000 159.00000

88.00000 139.00000

87.00000 118.00000

88.00000 98.00000

85.00000 77.00000

88.00000 57.00000

77.00000 40.00000

IMAGE=20190912LCNP02.jpg

ID=5

SCALE=0.031062

LM=0

CURVES=1

POINTS=100

379.00000 65.00000

387.00000 65.00000

396.00000 65.00000

404.00000 65.00000

413.00000 65.00000

421.00000 66.00000

429.00000 67.00000

438.00000 68.00000

446.00000 70.00000

454.00000 71.00000

463.00000 71.00000

471.00000 71.00000

479.00000 73.00000

487.00000 75.00000

495.00000 77.00000

503.00000 80.00000

511.00000 82.00000

519.00000 85.00000

527.00000 88.00000

535.00000 91.00000

542.00000 95.00000

550.00000 99.00000

557.00000 103.00000

564.00000 108.00000

571.00000 112.00000

578.00000 117.00000

584.00000 122.00000

591.00000 128.00000

597.00000 134.00000

603.00000 139.00000

609.00000 145.00000

614.00000 152.00000

620.00000 158.00000

625.00000 165.00000

629.00000 172.00000

632.00000 180.00000

636.00000 187.00000

641.00000 193.00000

647.00000 199.00000

651.00000 207.00000

654.00000 215.00000

658.00000 222.00000

660.00000 230.00000

662.00000 239.00000

663.00000 247.00000

664.00000 255.00000

664.00000 264.00000

665.00000 272.00000

666.00000 280.00000

669.00000 288.00000

670.00000 296.00000

661.00000 296.00000

653.00000 295.00000

645.00000 296.00000

636.00000 297.00000

628.00000 298.00000

620.00000 299.00000

611.00000 299.00000

603.00000 298.00000

595.00000 297.00000

586.00000 296.00000

578.00000 296.00000

569.00000 296.00000

561.00000 294.00000

553.00000 291.00000

545.00000 290.00000

538.00000 286.00000

531.00000 282.00000

523.00000 278.00000

517.00000 273.00000

510.00000 267.00000

504.00000 262.00000

497.00000 257.00000

490.00000 251.00000

484.00000 246.00000

477.00000 242.00000

470.00000 237.00000

463.00000 232.00000

457.00000 226.00000

451.00000 221.00000

444.00000 216.00000

439.00000 210.00000

432.00000 204.00000

427.00000 197.00000

421.00000 191.00000

416.00000 185.00000

413.00000 177.00000

409.00000 169.00000

404.00000 163.00000

400.00000 155.00000

397.00000 148.00000

395.00000 140.00000

394.00000 131.00000

392.00000 123.00000

389.00000 115.00000

386.00000 107.00000

383.00000 99.00000

380.00000 92.00000

377.00000 84.00000

372.00000 77.00000

IMAGE=20190916EUA01.jpg

ID=6

SCALE=0.031066

LM=0

CURVES=1

POINTS=100

454.00000 15.00000

457.00000 33.00000

464.00000 49.00000

470.00000 66.00000

473.00000 84.00000

475.00000 101.00000

477.00000 119.00000

482.00000 136.00000

484.00000 154.00000

487.00000 172.00000

485.00000 189.00000

481.00000 207.00000

477.00000 224.00000

473.00000 242.00000

466.00000 258.00000

459.00000 275.00000

451.00000 290.00000

441.00000 306.00000

435.00000 322.00000

428.00000 339.00000

420.00000 355.00000

412.00000 371.00000

401.00000 385.00000

391.00000 399.00000

381.00000 415.00000

371.00000 429.00000

361.00000 444.00000

351.00000 459.00000

342.00000 474.00000

332.00000 489.00000

322.00000 504.00000

311.00000 518.00000

300.00000 532.00000

286.00000 543.00000

274.00000 557.00000

261.00000 568.00000

245.00000 577.00000

232.00000 588.00000

219.00000 601.00000

205.00000 612.00000

190.00000 622.00000

174.00000 630.00000

160.00000 641.00000

146.00000 652.00000

132.00000 663.00000

117.00000 673.00000

101.00000 690.00000

85.00000 700.00000

70.00000 720.00000

62.00000 722.00000

63.00000 710.00000

72.00000 694.00000

83.00000 677.00000

86.00000 662.00000

92.00000 645.00000

92.00000 627.00000

89.00000 609.00000

86.00000 592.00000

82.00000 574.00000

80.00000 557.00000

80.00000 539.00000

80.00000 521.00000

80.00000 503.00000

82.00000 485.00000

84.00000 467.00000

85.00000 450.00000

90.00000 432.00000

93.00000 415.00000

93.00000 397.00000

97.00000 379.00000

104.00000 363.00000

111.00000 347.00000

118.00000 330.00000

123.00000 313.00000

128.00000 296.00000

135.00000 279.00000

142.00000 263.00000

150.00000 247.00000

157.00000 231.00000

163.00000 214.00000

171.00000 198.00000

182.00000 184.00000

193.00000 170.00000

204.00000 156.00000

215.00000 142.00000

226.00000 128.00000

239.00000 114.00000

249.00000 100.00000

261.00000 87.00000

276.00000 77.00000

289.00000 65.00000

302.00000 53.00000

317.00000 43.00000

332.00000 33.00000

349.00000 26.00000

365.00000 19.00000

383.00000 17.00000

400.00000 14.00000

418.00000 12.00000

436.00000 11.00000

IMAGE=20190916LCAF01.jpg

ID=7

SCALE=0.025806

LM=0

CURVES=1

POINTS=100

4059.00000 2099.00000

4183.00000 2114.00000

4305.00000 2086.00000

4417.00000 2030.00000

4499.00000 1935.00000

4563.00000 1828.00000

4620.00000 1717.00000

4668.00000 1601.00000

4698.00000 1479.00000

4714.00000 1355.00000

4704.00000 1230.00000

4685.00000 1107.00000

4639.00000 990.00000

4587.00000 877.00000

4530.00000 765.00000

4456.00000 664.00000

4371.00000 572.00000

4280.00000 486.00000

4178.00000 414.00000

4076.00000 341.00000

3966.00000 283.00000

3852.00000 231.00000

3735.00000 187.00000

3613.00000 157.00000

3488.00000 147.00000

3373.00000 100.00000

3250.00000 80.00000

3125.00000 69.00000

3000.00000 74.00000

2876.00000 92.00000

2752.00000 111.00000

2631.00000 141.00000

2517.00000 194.00000

2403.00000 246.00000

2294.00000 306.00000

2193.00000 381.00000

2104.00000 468.00000

2032.00000 570.00000

1964.00000 675.00000

1898.00000 782.00000

1828.00000 885.00000

1745.00000 979.00000

1655.00000 1066.00000

1585.00000 1170.00000

1513.00000 1272.00000

1421.00000 1357.00000

1350.00000 1460.00000

1300.00000 1575.00000

1248.00000 1689.00000

1191.00000 1800.00000

1130.00000 1909.00000

1088.00000 2027.00000

1051.00000 2147.00000

1018.00000 2268.00000

966.00000 2381.00000

1066.00000 2457.00000

1146.00000 2553.00000

1212.00000 2659.00000

1274.00000 2768.00000

1343.00000 2872.00000

1430.00000 2963.00000

1518.00000 3051.00000

1607.00000 3140.00000

1682.00000 3239.00000

1748.00000 3346.00000

1850.00000 3416.00000

1960.00000 3477.00000

2071.00000 3534.00000

2186.00000 3584.00000

2300.00000 3634.00000

2418.00000 3678.00000

2536.00000 3718.00000

2655.00000 3757.00000

2778.00000 3782.00000

2901.00000 3803.00000

3026.00000 3803.00000

3150.00000 3785.00000

3268.00000 3744.00000

3386.00000 3703.00000

3504.00000 3659.00000

3617.00000 3607.00000

3731.00000 3554.00000

3850.00000 3515.00000

3969.00000 3476.00000

4079.00000 3418.00000

4189.00000 3357.00000

4278.00000 3269.00000

4374.00000 3189.00000

4449.00000 3089.00000

4512.00000 2981.00000

4580.00000 2876.00000

4634.00000 2763.00000

4652.00000 2639.00000

4652.00000 2514.00000

4610.00000 2398.00000

4517.00000 2314.00000

4414.00000 2243.00000

4303.00000 2185.00000

4182.00000 2151.00000

4066.00000 2105.00000

IMAGE=20190918LNEO01.JPG

ID=8

SCALE=0.009679

LM=0

CURVES=1

POINTS=100

3749.00000 1993.00000

3856.00000 1988.00000

3963.00000 1982.00000

4071.00000 1985.00000

4176.00000 2005.00000

4281.00000 2026.00000

4385.00000 2055.00000

4457.00000 2132.00000

4476.00000 2238.00000

4497.00000 2343.00000

4510.00000 2449.00000

4510.00000 2557.00000

4508.00000 2664.00000

4477.00000 2766.00000

4439.00000 2867.00000

4374.00000 2952.00000

4301.00000 3031.00000

4224.00000 3105.00000

4147.00000 3180.00000

4062.00000 3246.00000

3968.00000 3298.00000

3873.00000 3347.00000

3771.00000 3382.00000

3684.00000 3443.00000

3589.00000 3495.00000

3490.00000 3534.00000

3385.00000 3557.00000

3278.00000 3568.00000

3171.00000 3575.00000

3066.00000 3554.00000

2964.00000 3521.00000

2864.00000 3482.00000

2762.00000 3447.00000

2660.00000 3414.00000

2560.00000 3374.00000

2464.00000 3326.00000

2378.00000 3263.00000

2298.00000 3191.00000

2228.00000 3110.00000

2158.00000 3028.00000

2077.00000 2958.00000

1997.00000 2886.00000

1932.00000 2801.00000

1876.00000 2709.00000

1819.00000 2618.00000

1761.00000 2528.00000

1703.00000 2438.00000

1663.00000 2338.00000

1638.00000 2234.00000

1620.00000 2128.00000

1595.00000 2024.00000

1538.00000 1933.00000

1462.00000 1858.00000

1521.00000 1777.00000

1587.00000 1693.00000

1657.00000 1611.00000

1680.00000 1509.00000

1699.00000 1403.00000

1742.00000 1305.00000

1800.00000 1215.00000

1890.00000 1156.00000

1979.00000 1096.00000

2064.00000 1032.00000

2148.00000 965.00000

2235.00000 902.00000

2324.00000 842.00000

2412.00000 779.00000

2494.00000 711.00000

2584.00000 652.00000

2676.00000 597.00000

2774.00000 552.00000

2873.00000 511.00000

2978.00000 491.00000

3084.00000 472.00000

3191.00000 462.00000

3298.00000 466.00000

3402.00000 490.00000

3505.00000 521.00000

3604.00000 563.00000

3700.00000 611.00000

3796.00000 659.00000

3893.00000 704.00000

3990.00000 749.00000

4073.00000 818.00000

4152.00000 891.00000

4213.00000 978.00000

4273.00000 1068.00000

4315.00000 1167.00000

4350.00000 1268.00000

4370.00000 1373.00000

4378.00000 1480.00000

4374.00000 1588.00000

4356.00000 1693.00000

4294.00000 1781.00000

4232.00000 1869.00000

4155.00000 1943.00000

4073.00000 2006.00000

3966.00000 2012.00000

3859.00000 2001.00000

3752.00000 1993.00000

IMAGE=20190918LNEO02.JPG

ID=9

SCALE=0.009980

LM=0

CURVES=1

POINTS=100

3240.00000 3703.00000

3191.00000 3684.00000

3146.00000 3656.00000

3103.00000 3626.00000

3067.00000 3587.00000

3033.00000 3546.00000

3008.00000 3499.00000

2984.00000 3452.00000

2967.00000 3402.00000

2949.00000 3352.00000

2941.00000 3299.00000

2937.00000 3246.00000

2929.00000 3194.00000

2921.00000 3141.00000

2911.00000 3089.00000

2902.00000 3037.00000

2893.00000 2985.00000

2887.00000 2932.00000

2881.00000 2879.00000

2876.00000 2826.00000

2870.00000 2774.00000

2865.00000 2721.00000

2860.00000 2668.00000

2856.00000 2615.00000

2854.00000 2562.00000

2852.00000 2509.00000

2850.00000 2456.00000

2848.00000 2403.00000

2848.00000 2350.00000

2856.00000 2297.00000

2859.00000 2244.00000

2860.00000 2191.00000

2861.00000 2138.00000

2862.00000 2085.00000

2865.00000 2032.00000

2870.00000 1980.00000

2878.00000 1927.00000

2892.00000 1876.00000

2905.00000 1824.00000

2915.00000 1773.00000

2921.00000 1720.00000

2928.00000 1667.00000

2939.00000 1615.00000

2959.00000 1567.00000

2988.00000 1522.00000

3027.00000 1479.00000

3067.00000 1448.00000

3104.00000 1410.00000

3144.00000 1339.00000

3138.00000 1379.00000

3153.00000 1410.00000

3193.00000 1434.00000

3243.00000 1452.00000

3291.00000 1473.00000

3337.00000 1500.00000

3372.00000 1538.00000

3403.00000 1581.00000

3434.00000 1624.00000

3460.00000 1670.00000

3485.00000 1717.00000

3510.00000 1764.00000

3534.00000 1812.00000

3554.00000 1860.00000

3571.00000 1911.00000

3586.00000 1962.00000

3597.00000 2014.00000

3607.00000 2066.00000

3615.00000 2118.00000

3624.00000 2171.00000

3630.00000 2223.00000

3635.00000 2276.00000

3640.00000 2329.00000

3645.00000 2382.00000

3650.00000 2435.00000

3655.00000 2487.00000

3659.00000 2540.00000

3662.00000 2593.00000

3664.00000 2646.00000

3662.00000 2699.00000

3660.00000 2752.00000

3655.00000 2805.00000

3656.00000 2858.00000

3653.00000 2911.00000

3652.00000 2964.00000

3649.00000 3017.00000

3643.00000 3070.00000

3632.00000 3122.00000

3620.00000 3173.00000

3604.00000 3224.00000

3588.00000 3275.00000

3569.00000 3324.00000

3545.00000 3372.00000

3520.00000 3419.00000

3493.00000 3464.00000

3460.00000 3506.00000

3427.00000 3547.00000

3387.00000 3582.00000

3345.00000 3614.00000

3306.00000 3650.00000

3283.00000 3698.00000

IMAGE=20190919LCRP01.JPG

ID=10

SCALE=0.010222

LM=0

CURVES=1

POINTS=100

3407.00000 2769.00000

3415.00000 2706.00000

3421.00000 2643.00000

3424.00000 2580.00000

3423.00000 2517.00000

3421.00000 2454.00000

3417.00000 2391.00000

3409.00000 2328.00000

3394.00000 2267.00000

3378.00000 2206.00000

3363.00000 2145.00000

3344.00000 2084.00000

3320.00000 2026.00000

3295.00000 1968.00000

3271.00000 1910.00000

3246.00000 1852.00000

3219.00000 1794.00000

3193.00000 1737.00000

3161.00000 1683.00000

3131.00000 1627.00000

3097.00000 1573.00000

3065.00000 1519.00000

3034.00000 1464.00000

3000.00000 1411.00000

2966.00000 1358.00000

2931.00000 1305.00000

2897.00000 1252.00000

2860.00000 1201.00000

2820.00000 1152.00000

2780.00000 1103.00000

2740.00000 1054.00000

2701.00000 1004.00000

2660.00000 956.00000

2622.00000 906.00000

2583.00000 856.00000

2540.00000 810.00000

2493.00000 768.00000

2447.00000 725.00000

2398.00000 684.00000

2349.00000 646.00000

2298.00000 608.00000

2243.00000 577.00000

2186.00000 550.00000

2128.00000 525.00000

2068.00000 505.00000

2006.00000 494.00000

1943.00000 489.00000

1880.00000 489.00000

1817.00000 499.00000

1754.00000 503.00000

1665.00000 477.00000

1682.00000 514.00000

1677.00000 577.00000

1667.00000 639.00000

1653.00000 701.00000

1644.00000 763.00000

1649.00000 826.00000

1656.00000 889.00000

1671.00000 950.00000

1697.00000 1008.00000

1721.00000 1066.00000

1745.00000 1125.00000

1770.00000 1183.00000

1795.00000 1241.00000

1820.00000 1299.00000

1847.00000 1356.00000

1876.00000 1411.00000

1907.00000 1467.00000

1941.00000 1520.00000

1977.00000 1572.00000

2013.00000 1623.00000

2050.00000 1674.00000

2089.00000 1725.00000

2127.00000 1775.00000

2167.00000 1824.00000

2209.00000 1871.00000

2252.00000 1917.00000

2295.00000 1964.00000

2338.00000 2010.00000

2380.00000 2057.00000

2423.00000 2103.00000

2465.00000 2150.00000

2508.00000 2197.00000

2551.00000 2242.00000

2595.00000 2288.00000

2643.00000 2330.00000

2688.00000 2374.00000

2734.00000 2417.00000

2780.00000 2460.00000

2827.00000 2502.00000

2875.00000 2544.00000

2925.00000 2581.00000

2976.00000 2619.00000

3027.00000 2657.00000

3083.00000 2685.00000

3140.00000 2713.00000

3198.00000 2738.00000

3258.00000 2757.00000

3319.00000 2775.00000

3381.00000 2785.00000

IMAGE=20190919LCRP02.JPG

ID=11

SCALE=0.009934

LM=0

CURVES=1

POINTS=100

1907.00000 1800.00000

1861.00000 1726.00000

1838.00000 1642.00000

1852.00000 1556.00000

1894.00000 1480.00000

1947.00000 1410.00000

2003.00000 1344.00000

2072.00000 1291.00000

2154.00000 1261.00000

2237.00000 1235.00000

2321.00000 1212.00000

2405.00000 1191.00000

2492.00000 1193.00000

2579.00000 1198.00000

2665.00000 1213.00000

2747.00000 1240.00000

2826.00000 1277.00000

2905.00000 1314.00000

2982.00000 1355.00000

3055.00000 1403.00000

3123.00000 1457.00000

3188.00000 1514.00000

3247.00000 1579.00000

3306.00000 1643.00000

3364.00000 1708.00000

3417.00000 1777.00000

3473.00000 1844.00000

3521.00000 1916.00000

3554.00000 1997.00000

3582.00000 2079.00000

3598.00000 2165.00000

3618.00000 2250.00000

3646.00000 2332.00000

3674.00000 2414.00000

3700.00000 2498.00000

3699.00000 2585.00000

3692.00000 2671.00000

3674.00000 2757.00000

3657.00000 2842.00000

3638.00000 2927.00000

3611.00000 3010.00000

3576.00000 3089.00000

3537.00000 3167.00000

3496.00000 3244.00000

3450.00000 3318.00000

3399.00000 3388.00000

3348.00000 3459.00000

3296.00000 3529.00000

3231.00000 3642.00000

3203.00000 3713.00000

3151.00000 3717.00000

3063.00000 3720.00000

2976.00000 3722.00000

2891.00000 3706.00000

2804.00000 3703.00000

2717.00000 3706.00000

2631.00000 3718.00000

2544.00000 3711.00000

2458.00000 3696.00000

2376.00000 3668.00000

2293.00000 3639.00000

2209.00000 3619.00000

2124.00000 3599.00000

2039.00000 3579.00000

1954.00000 3563.00000

1867.00000 3559.00000

1781.00000 3546.00000

1701.00000 3511.00000

1633.00000 3456.00000

1571.00000 3396.00000

1503.00000 3341.00000

1433.00000 3289.00000

1360.00000 3242.00000

1296.00000 3182.00000

1248.00000 3110.00000

1207.00000 3033.00000

1168.00000 2956.00000

1131.00000 2877.00000

1099.00000 2796.00000

1082.00000 2710.00000

1071.00000 2624.00000

1060.00000 2538.00000

1049.00000 2451.00000

1045.00000 2364.00000

1046.00000 2277.00000

1050.00000 2190.00000

1065.00000 2105.00000

1084.00000 2020.00000

1124.00000 1942.00000

1169.00000 1868.00000

1231.00000 1806.00000

1294.00000 1747.00000

1370.00000 1704.00000

1449.00000 1667.00000

1533.00000 1645.00000

1619.00000 1633.00000

1706.00000 1637.00000

1785.00000 1673.00000

1842.00000 1739.00000

1906.00000 1798.00000

IMAGE=20190925LNEO01.JPG

ID=12

SCALE=0.008830

LM=0

CURVES=1

POINTS=100

3732.00000 2396.00000

3830.00000 2383.00000

3928.00000 2384.00000

4024.00000 2405.00000

4115.00000 2442.00000

4187.00000 2509.00000

4251.00000 2584.00000

4293.00000 2673.00000

4285.00000 2771.00000

4269.00000 2868.00000

4244.00000 2963.00000

4211.00000 3056.00000

4168.00000 3145.00000

4124.00000 3233.00000

4055.00000 3302.00000

3985.00000 3372.00000

3910.00000 3436.00000

3828.00000 3491.00000

3741.00000 3537.00000

3651.00000 3576.00000

3558.00000 3607.00000

3464.00000 3637.00000

3370.00000 3666.00000

3276.00000 3695.00000

3182.00000 3724.00000

3088.00000 3754.00000

2993.00000 3780.00000

2897.00000 3801.00000

2800.00000 3816.00000

2702.00000 3806.00000

2606.00000 3785.00000

2513.00000 3753.00000

2420.00000 3719.00000

2328.00000 3685.00000

2239.00000 3644.00000

2154.00000 3594.00000

2068.00000 3546.00000

1986.00000 3492.00000

1906.00000 3435.00000

1825.00000 3379.00000

1748.00000 3318.00000

1675.00000 3251.00000

1611.00000 3176.00000

1553.00000 3097.00000

1503.00000 3012.00000

1456.00000 2926.00000

1419.00000 2835.00000

1401.00000 2738.00000

1384.00000 2641.00000

1356.00000 2547.00000

1337.00000 2450.00000

1381.00000 2362.00000

1403.00000 2266.00000

1408.00000 2168.00000

1422.00000 2071.00000

1448.00000 1976.00000

1476.00000 1881.00000

1510.00000 1789.00000

1545.00000 1697.00000

1592.00000 1611.00000

1648.00000 1530.00000

1706.00000 1450.00000

1774.00000 1379.00000

1844.00000 1310.00000

1922.00000 1250.00000

2003.00000 1195.00000

2087.00000 1143.00000

2180.00000 1113.00000

2276.00000 1089.00000

2365.00000 1048.00000

2459.00000 1017.00000

2553.00000 990.00000

2650.00000 972.00000

2748.00000 963.00000

2846.00000 962.00000

2945.00000 959.00000

3043.00000 971.00000

3139.00000 992.00000

3230.00000 1027.00000

3322.00000 1064.00000

3412.00000 1104.00000

3499.00000 1150.00000

3585.00000 1197.00000

3673.00000 1241.00000

3764.00000 1278.00000

3855.00000 1315.00000

3936.00000 1371.00000

3999.00000 1447.00000

4036.00000 1538.00000

4080.00000 1626.00000

4110.00000 1719.00000

4129.00000 1816.00000

4121.00000 1914.00000

4108.00000 2012.00000

4084.00000 2107.00000

4043.00000 2196.00000

3988.00000 2278.00000

3918.00000 2347.00000

3821.00000 2362.00000

3726.00000 2387.00000

IMAGE=20190925LNEO02.JPG

ID=13

SCALE=0.010243

LM=0

CURVES=1

POINTS=100

373.00000 88.00000

369.00000 95.00000

365.00000 102.00000

357.00000 106.00000

351.00000 112.00000

345.00000 117.00000

339.00000 123.00000

332.00000 128.00000

326.00000 133.00000

319.00000 138.00000

312.00000 143.00000

306.00000 148.00000

298.00000 152.00000

292.00000 157.00000

285.00000 162.00000

278.00000 166.00000

270.00000 170.00000

263.00000 173.00000

255.00000 176.00000

247.00000 179.00000

239.00000 181.00000

231.00000 184.00000

224.00000 188.00000

216.00000 190.00000

208.00000 193.00000

200.00000 195.00000

192.00000 197.00000

184.00000 198.00000

176.00000 199.00000

168.00000 201.00000

159.00000 201.00000

151.00000 201.00000

143.00000 201.00000

134.00000 201.00000

126.00000 202.00000

118.00000 202.00000

110.00000 200.00000

102.00000 197.00000

94.00000 195.00000

85.00000 194.00000

78.00000 191.00000

70.00000 187.00000

63.00000 184.00000

55.00000 181.00000

47.00000 177.00000

40.00000 174.00000

32.00000 171.00000

24.00000 169.00000

18.00000 166.00000

24.00000 158.00000

30.00000 152.00000

35.00000 147.00000

41.00000 140.00000

46.00000 134.00000

52.00000 128.00000

56.00000 121.00000

62.00000 114.00000

67.00000 108.00000

72.00000 101.00000

77.00000 94.00000

82.00000 89.00000

88.00000 82.00000

95.00000 77.00000

100.00000 72.00000

107.00000 66.00000

113.00000 60.00000

120.00000 56.00000

127.00000 51.00000

134.00000 47.00000

141.00000 42.00000

148.00000 38.00000

156.00000 36.00000

164.00000 33.00000

172.00000 31.00000

180.00000 29.00000

188.00000 27.00000

196.00000 24.00000

204.00000 21.00000

212.00000 20.00000

220.00000 20.00000

228.00000 20.00000

237.00000 21.00000

245.00000 22.00000

253.00000 22.00000

261.00000 24.00000

269.00000 27.00000

277.00000 29.00000

284.00000 33.00000

292.00000 37.00000

299.00000 40.00000

308.00000 42.00000

315.00000 45.00000

323.00000 47.00000

331.00000 50.00000

338.00000 54.00000

346.00000 57.00000

354.00000 61.00000

361.00000 65.00000

367.00000 70.00000

374.00000 75.00000

IMAGE=20190927LEUA01.jpg

ID=14

SCALE=0.009708

LM=0

CURVES=1

POINTS=100

574.00000 689.00000

595.00000 686.00000

616.00000 680.00000

636.00000 670.00000

654.00000 658.00000

666.00000 641.00000

677.00000 622.00000

684.00000 601.00000

691.00000 581.00000

693.00000 559.00000

693.00000 538.00000

694.00000 516.00000

694.00000 494.00000

691.00000 473.00000

685.00000 452.00000

678.00000 431.00000

672.00000 411.00000

660.00000 393.00000

646.00000 377.00000

631.00000 360.00000

617.00000 344.00000

601.00000 329.00000

585.00000 315.00000

571.00000 298.00000

556.00000 282.00000

546.00000 263.00000

535.00000 245.00000

519.00000 230.00000

503.00000 216.00000

486.00000 202.00000

471.00000 187.00000

453.00000 174.00000

434.00000 164.00000

416.00000 152.00000

397.00000 141.00000

378.00000 131.00000

358.00000 123.00000

337.00000 117.00000

316.00000 111.00000

295.00000 106.00000

275.00000 98.00000

255.00000 90.00000

234.00000 85.00000

213.00000 79.00000

192.00000 73.00000

173.00000 64.00000

152.00000 56.00000

131.00000 53.00000

110.00000 48.00000

90.00000 39.00000

74.00000 25.00000

60.00000 9.00000

53.00000 16.00000

64.00000 35.00000

76.00000 53.00000

77.00000 74.00000

76.00000 96.00000

76.00000 118.00000

71.00000 139.00000

66.00000 160.00000

62.00000 181.00000

64.00000 203.00000

68.00000 224.00000

71.00000 245.00000

71.00000 267.00000

70.00000 289.00000

71.00000 310.00000

74.00000 332.00000

73.00000 353.00000

79.00000 374.00000

86.00000 395.00000

94.00000 415.00000

102.00000 435.00000

110.00000 455.00000

117.00000 476.00000

129.00000 493.00000

142.00000 511.00000

153.00000 530.00000

165.00000 548.00000

178.00000 565.00000

190.00000 583.00000

205.00000 599.00000

219.00000 615.00000

234.00000 631.00000

249.00000 647.00000

265.00000 661.00000

284.00000 671.00000

304.00000 680.00000

324.00000 687.00000

343.00000 697.00000

363.00000 705.00000

383.00000 714.00000

403.00000 723.00000

425.00000 725.00000

446.00000 721.00000

467.00000 719.00000

489.00000 716.00000

508.00000 706.00000

529.00000 700.00000

550.00000 695.00000

IMAGE=20191011LALA01.jpg

ID=15

SCALE=0.026015

LM=0

CURVES=1

POINTS=100

232.00000 69.00000

208.00000 68.00000

185.00000 70.00000

161.00000 68.00000

139.00000 61.00000

117.00000 69.00000

96.00000 80.00000

78.00000 96.00000

63.00000 114.00000

52.00000 135.00000

43.00000 157.00000

34.00000 179.00000

25.00000 201.00000

23.00000 225.00000

22.00000 248.00000

23.00000 272.00000

24.00000 296.00000

28.00000 319.00000

33.00000 342.00000

42.00000 364.00000

52.00000 386.00000

63.00000 406.00000

75.00000 427.00000

90.00000 446.00000

104.00000 464.00000

120.00000 482.00000

133.00000 502.00000

148.00000 520.00000

165.00000 537.00000

183.00000 552.00000

201.00000 567.00000

221.00000 580.00000

241.00000 593.00000

260.00000 608.00000

280.00000 620.00000

299.00000 634.00000

320.00000 646.00000

339.00000 659.00000

360.00000 670.00000

381.00000 682.00000

401.00000 695.00000

421.00000 707.00000

441.00000 720.00000

460.00000 734.00000

478.00000 749.00000

499.00000 760.00000

520.00000 771.00000

542.00000 781.00000

563.00000 791.00000

585.00000 800.00000

608.00000 807.00000

629.00000 817.00000

647.00000 817.00000

650.00000 794.00000

655.00000 771.00000

661.00000 748.00000

669.00000 725.00000

676.00000 703.00000

683.00000 680.00000

689.00000 657.00000

690.00000 634.00000

697.00000 611.00000

700.00000 588.00000

703.00000 564.00000

704.00000 541.00000

704.00000 517.00000

700.00000 494.00000

697.00000 470.00000

695.00000 447.00000

692.00000 423.00000

687.00000 400.00000

681.00000 377.00000

676.00000 354.00000

673.00000 331.00000

662.00000 310.00000

647.00000 292.00000

635.00000 272.00000

627.00000 250.00000

614.00000 230.00000

605.00000 208.00000

595.00000 186.00000

585.00000 165.00000

572.00000 145.00000

559.00000 125.00000

547.00000 105.00000

533.00000 86.00000

519.00000 66.00000

503.00000 49.00000

485.00000 34.00000

465.00000 22.00000

442.00000 16.00000

418.00000 13.00000

395.00000 8.00000

372.00000 6.00000

348.00000 12.00000

326.00000 20.00000

308.00000 35.00000

292.00000 52.00000

271.00000 63.00000

249.00000 70.00000

IMAGE=20191011LALA02.jpg

ID=16

SCALE=0.026013

LM=0

CURVES=1

POINTS=100

103.00000 18.00000

123.00000 23.00000

141.00000 34.00000

158.00000 45.00000

176.00000 55.00000

194.00000 65.00000

212.00000 75.00000

230.00000 85.00000

246.00000 98.00000

261.00000 111.00000

277.00000 124.00000

293.00000 138.00000

309.00000 151.00000

325.00000 164.00000

342.00000 175.00000

359.00000 186.00000

377.00000 197.00000

392.00000 210.00000

409.00000 223.00000

424.00000 237.00000

440.00000 250.00000

455.00000 263.00000

470.00000 278.00000

482.00000 294.00000

493.00000 312.00000

505.00000 329.00000

513.00000 347.00000

518.00000 367.00000

529.00000 385.00000

540.00000 401.00000

549.00000 420.00000

560.00000 437.00000

569.00000 456.00000

576.00000 475.00000

582.00000 495.00000

589.00000 514.00000

594.00000 534.00000

598.00000 554.00000

602.00000 574.00000

606.00000 595.00000

609.00000 615.00000

610.00000 635.00000

610.00000 656.00000

610.00000 677.00000

610.00000 697.00000

610.00000 718.00000

610.00000 738.00000

608.00000 759.00000

599.00000 776.00000

581.00000 786.00000

561.00000 791.00000

541.00000 792.00000

520.00000 791.00000

500.00000 788.00000

480.00000 783.00000

460.00000 777.00000

441.00000 771.00000

422.00000 763.00000

403.00000 756.00000

384.00000 748.00000

365.00000 739.00000

349.00000 727.00000

332.00000 714.00000

316.00000 702.00000

300.00000 689.00000

284.00000 676.00000

270.00000 661.00000

256.00000 646.00000

243.00000 630.00000

229.00000 615.00000

216.00000 598.00000

205.00000 581.00000

194.00000 564.00000

182.00000 547.00000

170.00000 531.00000

160.00000 513.00000

150.00000 495.00000

142.00000 476.00000

134.00000 457.00000

127.00000 437.00000

123.00000 417.00000

115.00000 398.00000

107.00000 379.00000

102.00000 360.00000

99.00000 339.00000

98.00000 319.00000

94.00000 298.00000

89.00000 279.00000

83.00000 259.00000

77.00000 239.00000

74.00000 219.00000

73.00000 198.00000

73.00000 178.00000

74.00000 157.00000

74.00000 137.00000

73.00000 116.00000

74.00000 96.00000

75.00000 75.00000

78.00000 55.00000

84.00000 35.00000

IMAGE=20191014LCNP01.jpg

ID=17

SCALE=0.034774

LM=0

CURVES=1

POINTS=100

2661.00000 3390.00000

2647.00000 3338.00000

2633.00000 3286.00000

2620.00000 3233.00000

2605.00000 3181.00000

2593.00000 3129.00000

2582.00000 3076.00000

2579.00000 3022.00000

2579.00000 2968.00000

2578.00000 2914.00000

2578.00000 2860.00000

2588.00000 2807.00000

2601.00000 2754.00000

2612.00000 2702.00000

2630.00000 2651.00000

2647.00000 2599.00000

2664.00000 2548.00000

2677.00000 2496.00000

2694.00000 2444.00000

2714.00000 2395.00000

2738.00000 2346.00000

2760.00000 2297.00000

2783.00000 2248.00000

2810.00000 2201.00000

2842.00000 2158.00000

2871.00000 2113.00000

2898.00000 2066.00000

2926.00000 2019.00000

2952.00000 1972.00000

2982.00000 1928.00000

3015.00000 1885.00000

3046.00000 1840.00000

3076.00000 1796.00000

3104.00000 1750.00000

3139.00000 1708.00000

3175.00000 1669.00000

3212.00000 1629.00000

3248.00000 1588.00000

3286.00000 1551.00000

3328.00000 1517.00000

3370.00000 1483.00000

3413.00000 1450.00000

3456.00000 1418.00000

3504.00000 1392.00000

3555.00000 1375.00000

3608.00000 1366.00000

3662.00000 1360.00000

3712.00000 1346.00000

3755.00000 1313.00000

3801.00000 1286.00000

3852.00000 1267.00000

3828.00000 1290.00000

3801.00000 1334.00000

3796.00000 1387.00000

3807.00000 1439.00000

3822.00000 1491.00000

3839.00000 1542.00000

3852.00000 1595.00000

3856.00000 1648.00000

3848.00000 1701.00000

3849.00000 1755.00000

3846.00000 1808.00000

3837.00000 1861.00000

3820.00000 1913.00000

3800.00000 1963.00000

3781.00000 2013.00000

3761.00000 2063.00000

3742.00000 2114.00000

3722.00000 2164.00000

3706.00000 2216.00000

3684.00000 2265.00000

3657.00000 2311.00000

3631.00000 2359.00000

3601.00000 2403.00000

3577.00000 2451.00000

3560.00000 2503.00000

3544.00000 2554.00000

3522.00000 2603.00000

3494.00000 2650.00000

3465.00000 2695.00000

3437.00000 2741.00000

3408.00000 2787.00000

3379.00000 2832.00000

3348.00000 2877.00000

3316.00000 2920.00000

3282.00000 2962.00000

3248.00000 3004.00000

3215.00000 3047.00000

3182.00000 3089.00000

3144.00000 3128.00000

3107.00000 3168.00000

3070.00000 3206.00000

3031.00000 3245.00000

2987.00000 3276.00000

2943.00000 3306.00000

2898.00000 3336.00000

2853.00000 3366.00000

2804.00000 3389.00000

2753.00000 3408.00000

2703.00000 3427.00000

IMAGE=20191014LCRP01.JPG

ID=18

SCALE=0.002022

LM=0

CURVES=1

POINTS=100

2322.00000 3011.00000

2308.00000 2953.00000

2297.00000 2894.00000

2294.00000 2834.00000

2306.00000 2775.00000

2313.00000 2716.00000

2319.00000 2656.00000

2342.00000 2601.00000

2360.00000 2544.00000

2378.00000 2487.00000

2409.00000 2435.00000

2443.00000 2386.00000

2471.00000 2333.00000

2503.00000 2282.00000

2539.00000 2235.00000

2578.00000 2189.00000

2621.00000 2146.00000

2661.00000 2102.00000

2701.00000 2057.00000

2739.00000 2011.00000

2781.00000 1968.00000

2821.00000 1924.00000

2869.00000 1888.00000

2917.00000 1852.00000

2959.00000 1809.00000

2993.00000 1760.00000

3036.00000 1718.00000

3081.00000 1678.00000

3123.00000 1635.00000

3169.00000 1597.00000

3214.00000 1558.00000

3259.00000 1517.00000

3307.00000 1482.00000

3357.00000 1448.00000

3406.00000 1414.00000

3455.00000 1380.00000

3507.00000 1350.00000

3558.00000 1318.00000

3610.00000 1288.00000

3665.00000 1264.00000

3721.00000 1243.00000

3778.00000 1223.00000

3837.00000 1212.00000

3896.00000 1202.00000

3955.00000 1196.00000

4015.00000 1196.00000

4075.00000 1205.00000

4133.00000 1219.00000

4187.00000 1244.00000

4245.00000 1260.00000

4305.00000 1259.00000

4386.00000 1253.00000

4360.00000 1264.00000

4305.00000 1288.00000

4272.00000 1337.00000

4262.00000 1396.00000

4252.00000 1455.00000

4238.00000 1514.00000

4224.00000 1572.00000

4202.00000 1628.00000

4169.00000 1678.00000

4136.00000 1728.00000

4102.00000 1778.00000

4068.00000 1827.00000

4031.00000 1874.00000

3979.00000 1903.00000

3941.00000 1949.00000

3905.00000 1997.00000

3867.00000 2043.00000

3826.00000 2087.00000

3785.00000 2131.00000

3743.00000 2173.00000

3698.00000 2214.00000

3654.00000 2254.00000

3610.00000 2295.00000

3565.00000 2335.00000

3521.00000 2375.00000

3475.00000 2414.00000

3430.00000 2453.00000

3384.00000 2492.00000

3339.00000 2531.00000

3293.00000 2571.00000

3247.00000 2609.00000

3201.00000 2647.00000

3154.00000 2685.00000

3108.00000 2723.00000

3062.00000 2761.00000

3011.00000 2793.00000

2960.00000 2824.00000

2909.00000 2856.00000

2858.00000 2888.00000

2806.00000 2917.00000

2753.00000 2945.00000

2700.00000 2973.00000

2645.00000 2996.00000

2588.00000 3016.00000

2532.00000 3037.00000

2473.00000 3048.00000

2413.00000 3050.00000

2354.00000 3039.00000

IMAGE=20191014LCRP02.JPG

ID=19

SCALE=0.008573

LM=0

CURVES=1

POINTS=100

175.00000 151.00000

185.00000 160.00000

191.00000 172.00000

198.00000 183.00000

206.00000 194.00000

211.00000 206.00000

217.00000 218.00000

222.00000 231.00000

227.00000 243.00000

231.00000 256.00000

235.00000 269.00000

238.00000 282.00000

240.00000 295.00000

242.00000 308.00000

245.00000 321.00000

247.00000 334.00000

248.00000 348.00000

248.00000 361.00000

251.00000 374.00000

252.00000 387.00000

252.00000 401.00000

254.00000 414.00000

251.00000 427.00000

248.00000 440.00000

244.00000 453.00000

241.00000 466.00000

238.00000 479.00000

236.00000 492.00000

232.00000 505.00000

227.00000 517.00000

222.00000 530.00000

214.00000 540.00000

209.00000 553.00000

202.00000 564.00000

195.00000 576.00000

188.00000 587.00000

179.00000 597.00000

168.00000 605.00000

158.00000 614.00000

148.00000 622.00000

139.00000 631.00000

130.00000 642.00000

122.00000 653.00000

113.00000 663.00000

104.00000 672.00000

94.00000 681.00000

84.00000 690.00000

75.00000 700.00000

66.00000 710.00000

55.00000 727.00000

48.00000 733.00000

42.00000 727.00000

43.00000 713.00000

43.00000 699.00000

42.00000 686.00000

41.00000 673.00000

40.00000 659.00000

39.00000 646.00000

35.00000 633.00000

32.00000 620.00000

29.00000 607.00000

26.00000 594.00000

21.00000 582.00000

16.00000 569.00000

13.00000 556.00000

10.00000 543.00000

8.00000 530.00000

7.00000 517.00000

6.00000 503.00000

7.00000 490.00000

9.00000 477.00000

8.00000 464.00000

8.00000 450.00000

10.00000 437.00000

10.00000 424.00000

13.00000 411.00000

17.00000 398.00000

20.00000 385.00000

23.00000 372.00000

25.00000 359.00000

26.00000 346.00000

28.00000 333.00000

31.00000 320.00000

35.00000 307.00000

39.00000 294.00000

45.00000 282.00000

52.00000 270.00000

59.00000 259.00000

66.00000 248.00000

74.00000 237.00000

82.00000 226.00000

90.00000 216.00000

100.00000 206.00000

108.00000 196.00000

116.00000 186.00000

125.00000 176.00000

137.00000 169.00000

148.00000 162.00000

158.00000 153.00000

168.00000 144.00000

IMAGE=20191018LEUA01.jpg

ID=20

SCALE=0.009523

LM=0

CURVES=1

POINTS=100

222.00000 682.00000

212.00000 668.00000

201.00000 655.00000

191.00000 642.00000

179.00000 629.00000

171.00000 614.00000

162.00000 600.00000

156.00000 584.00000

150.00000 568.00000

145.00000 551.00000

143.00000 534.00000

145.00000 517.00000

146.00000 500.00000

147.00000 483.00000

149.00000 467.00000

151.00000 450.00000

153.00000 433.00000

157.00000 416.00000

163.00000 400.00000

169.00000 384.00000

175.00000 368.00000

180.00000 352.00000

185.00000 336.00000

190.00000 319.00000

194.00000 303.00000

202.00000 287.00000

211.00000 273.00000

217.00000 257.00000

226.00000 243.00000

234.00000 228.00000

243.00000 213.00000

255.00000 201.00000

265.00000 187.00000

276.00000 175.00000

287.00000 162.00000

299.00000 149.00000

313.00000 140.00000

328.00000 132.00000

344.00000 125.00000

359.00000 118.00000

374.00000 109.00000

390.00000 104.00000

407.00000 99.00000

423.00000 94.00000

440.00000 90.00000

457.00000 90.00000

473.00000 87.00000

489.00000 80.00000

502.00000 69.00000

514.00000 57.00000

525.00000 44.00000

535.00000 30.00000

546.00000 17.00000

549.00000 25.00000

540.00000 40.00000

529.00000 53.00000

525.00000 70.00000

521.00000 86.00000

513.00000 101.00000

503.00000 115.00000

492.00000 129.00000

484.00000 143.00000

478.00000 159.00000

477.00000 176.00000

482.00000 193.00000

487.00000 209.00000

491.00000 225.00000

493.00000 242.00000

496.00000 259.00000

499.00000 276.00000

499.00000 293.00000

498.00000 310.00000

497.00000 327.00000

493.00000 344.00000

490.00000 360.00000

488.00000 377.00000

483.00000 394.00000

477.00000 410.00000

469.00000 425.00000

462.00000 440.00000

455.00000 456.00000

449.00000 472.00000

442.00000 487.00000

435.00000 503.00000

427.00000 518.00000

419.00000 533.00000

410.00000 548.00000

400.00000 561.00000

391.00000 575.00000

377.00000 585.00000

363.00000 596.00000

350.00000 607.00000

336.00000 616.00000

323.00000 628.00000

310.00000 638.00000

295.00000 647.00000

281.00000 656.00000

264.00000 660.00000

248.00000 665.00000

237.00000 678.00000

IMAGE=20191021LCAF01.jpg

ID=21

SCALE=0.020381

LM=0

CURVES=1

POINTS=100

2580.00000 1689.00000

2630.00000 1599.00000

2686.00000 1512.00000

2744.00000 1426.00000

2742.00000 1334.00000

2770.00000 1241.00000

2855.00000 1182.00000

2952.00000 1147.00000

3053.00000 1125.00000

3155.00000 1114.00000

3258.00000 1124.00000

3359.00000 1145.00000

3456.00000 1181.00000

3546.00000 1232.00000

3623.00000 1301.00000

3699.00000 1371.00000

3784.00000 1430.00000

3857.00000 1502.00000

3917.00000 1586.00000

3978.00000 1670.00000

4031.00000 1758.00000

4075.00000 1852.00000

4109.00000 1950.00000

4137.00000 2049.00000

4156.00000 2151.00000

4174.00000 2253.00000

4178.00000 2356.00000

4175.00000 2459.00000

4153.00000 2560.00000

4124.00000 2659.00000

4085.00000 2755.00000

4041.00000 2848.00000

3986.00000 2936.00000

3931.00000 3023.00000

3876.00000 3111.00000

3806.00000 3187.00000

3732.00000 3259.00000

3660.00000 3334.00000

3590.00000 3409.00000

3517.00000 3483.00000

3430.00000 3538.00000

3342.00000 3593.00000

3252.00000 3643.00000

3161.00000 3692.00000

3072.00000 3745.00000

2978.00000 3787.00000

2884.00000 3831.00000

2784.00000 3858.00000

2684.00000 3884.00000

2581.00000 3882.00000

2480.00000 3863.00000

2379.00000 3843.00000

2279.00000 3816.00000

2179.00000 3789.00000

2078.00000 3765.00000

1986.00000 3719.00000

1896.00000 3669.00000

1802.00000 3624.00000

1713.00000 3572.00000

1630.00000 3511.00000

1550.00000 3445.00000

1471.00000 3379.00000

1387.00000 3319.00000

1309.00000 3251.00000

1239.00000 3174.00000

1184.00000 3087.00000

1128.00000 3000.00000

1080.00000 2909.00000

1043.00000 2812.00000

1007.00000 2715.00000

972.00000 2618.00000

939.00000 2520.00000

927.00000 2418.00000

921.00000 2314.00000

927.00000 2211.00000

933.00000 2108.00000

959.00000 2008.00000

1003.00000 1914.00000

1054.00000 1825.00000

1105.00000 1735.00000

1161.00000 1648.00000

1218.00000 1561.00000

1281.00000 1480.00000

1356.00000 1408.00000

1431.00000 1337.00000

1514.00000 1276.00000

1604.00000 1226.00000

1699.00000 1185.00000

1795.00000 1145.00000

1897.00000 1129.00000

2000.00000 1120.00000

2103.00000 1125.00000

2203.00000 1148.00000

2303.00000 1177.00000

2395.00000 1223.00000

2450.00000 1305.00000

2448.00000 1409.00000

2478.00000 1504.00000

2525.00000 1595.00000

2575.00000 1686.00000

IMAGE=20191021NEO01.JPG

ID=22

SCALE=0.009634

LM=0

CURVES=1

POINTS=100

2355.00000 2067.00000

2315.00000 2051.00000

2285.00000 2021.00000

2258.00000 1988.00000

2238.00000 1950.00000

2217.00000 1913.00000

2208.00000 1871.00000

2204.00000 1829.00000

2205.00000 1786.00000

2208.00000 1744.00000

2212.00000 1701.00000

2218.00000 1659.00000

2226.00000 1617.00000

2236.00000 1575.00000

2245.00000 1533.00000

2254.00000 1492.00000

2266.00000 1451.00000

2277.00000 1409.00000

2289.00000 1368.00000

2300.00000 1327.00000

2312.00000 1286.00000

2323.00000 1245.00000

2332.00000 1203.00000

2340.00000 1161.00000

2348.00000 1119.00000

2358.00000 1077.00000

2366.00000 1035.00000

2376.00000 994.00000

2386.00000 952.00000

2395.00000 911.00000

2402.00000 869.00000

2409.00000 826.00000

2416.00000 784.00000

2422.00000 742.00000

2428.00000 700.00000

2432.00000 657.00000

2438.00000 615.00000

2450.00000 574.00000

2457.00000 531.00000

2462.00000 489.00000

2467.00000 447.00000

2475.00000 405.00000

2488.00000 364.00000

2505.00000 325.00000

2532.00000 292.00000

2559.00000 259.00000

2592.00000 232.00000

2626.00000 207.00000

2663.00000 199.00000

2698.00000 190.00000

2723.00000 158.00000

2767.00000 189.00000

2791.00000 225.00000

2815.00000 260.00000

2826.00000 301.00000

2837.00000 342.00000

2848.00000 384.00000

2854.00000 426.00000

2858.00000 468.00000

2858.00000 511.00000

2859.00000 554.00000

2857.00000 597.00000

2854.00000 639.00000

2851.00000 682.00000

2848.00000 724.00000

2846.00000 767.00000

2847.00000 810.00000

2841.00000 852.00000

2837.00000 895.00000

2832.00000 937.00000

2828.00000 980.00000

2823.00000 1022.00000

2819.00000 1065.00000

2814.00000 1107.00000

2810.00000 1150.00000

2800.00000 1191.00000

2790.00000 1233.00000

2777.00000 1273.00000

2763.00000 1314.00000

2760.00000 1356.00000

2751.00000 1398.00000

2742.00000 1440.00000

2731.00000 1481.00000

2722.00000 1523.00000

2707.00000 1563.00000

2690.00000 1602.00000

2677.00000 1643.00000

2662.00000 1683.00000

2653.00000 1725.00000

2639.00000 1765.00000

2624.00000 1805.00000

2608.00000 1844.00000

2590.00000 1883.00000

2571.00000 1921.00000

2551.00000 1959.00000

2531.00000 1997.00000

2506.00000 2032.00000

2478.00000 2064.00000

2439.00000 2081.00000

2397.00000 2089.00000

IMAGE=20191023LNST02 - TUA.JPG

ID=23

SCALE=0.002412

LM=0

CURVES=1

POINTS=100

288.00000 233.00000

298.00000 240.00000

309.00000 245.00000

320.00000 251.00000

332.00000 256.00000

343.00000 260.00000

355.00000 264.00000

366.00000 269.00000

377.00000 274.00000

387.00000 281.00000

398.00000 287.00000

409.00000 293.00000

419.00000 299.00000

430.00000 305.00000

441.00000 311.00000

452.00000 317.00000

462.00000 324.00000

473.00000 330.00000

482.00000 338.00000

491.00000 346.00000

500.00000 354.00000

510.00000 362.00000

519.00000 371.00000

528.00000 379.00000

536.00000 388.00000

543.00000 398.00000

550.00000 409.00000

557.00000 418.00000

565.00000 428.00000

572.00000 438.00000

580.00000 447.00000

587.00000 458.00000

593.00000 468.00000

596.00000 480.00000

603.00000 490.00000

611.00000 500.00000

615.00000 511.00000

621.00000 522.00000

625.00000 534.00000

630.00000 545.00000

634.00000 557.00000

637.00000 568.00000

642.00000 580.00000

646.00000 591.00000

648.00000 603.00000

650.00000 616.00000

650.00000 628.00000

647.00000 640.00000

646.00000 659.00000

648.00000 667.00000

640.00000 666.00000

628.00000 663.00000

616.00000 659.00000

604.00000 656.00000

592.00000 653.00000

581.00000 649.00000

569.00000 645.00000

557.00000 643.00000

545.00000 641.00000

535.00000 634.00000

524.00000 629.00000

512.00000 625.00000

500.00000 622.00000

490.00000 615.00000

479.00000 609.00000

469.00000 602.00000

458.00000 596.00000

449.00000 588.00000

440.00000 580.00000

430.00000 573.00000

421.00000 564.00000

412.00000 555.00000

404.00000 546.00000

397.00000 536.00000

390.00000 526.00000

383.00000 516.00000

377.00000 505.00000

370.00000 495.00000

363.00000 485.00000

355.00000 476.00000

347.00000 466.00000

341.00000 455.00000

335.00000 444.00000

329.00000 434.00000

324.00000 423.00000

320.00000 411.00000

315.00000 400.00000

312.00000 388.00000

309.00000 376.00000

306.00000 364.00000

304.00000 352.00000

301.00000 340.00000

299.00000 328.00000

296.00000 316.00000

293.00000 304.00000

291.00000 292.00000

288.00000 280.00000

287.00000 267.00000

285.00000 255.00000

284.00000 243.00000

IMAGE=20191023LNST02.jpg

ID=24

SCALE=0.031066

LM=0

CURVES=1

POINTS=100

761.00000 465.00000

744.00000 473.00000

727.00000 478.00000

709.00000 482.00000

691.00000 485.00000

673.00000 482.00000

654.00000 482.00000

636.00000 483.00000

618.00000 480.00000

600.00000 478.00000

582.00000 475.00000

564.00000	472.00000
546.00000	468.00000
528.00000	465.00000
510.00000	461.00000
492.00000	457.00000
474.00000	452.00000
457.00000	447.00000
439.00000	442.00000
421.00000	437.00000
404.00000	432.00000
388.00000	423.00000
371.00000	415.00000
355.00000	407.00000
338.00000	400.00000
322.00000	391.00000
306.00000	382.00000
291.00000	371.00000
275.00000	362.00000
260.00000	352.00000
244.00000	343.00000
229.00000	332.00000
215.00000	321.00000
200.00000	309.00000
187.00000	297.00000
172.00000	286.00000
157.00000	275.00000
144.00000	262.00000
133.00000	248.00000
122.00000	233.00000
115.00000	216.00000
111.00000	198.00000

103.00000 182.00000

99.00000 164.00000

89.00000 149.00000

84.00000 131.00000

79.00000 113.00000

68.00000 99.00000

53.00000 89.00000

57.00000 78.00000

75.00000 75.00000

93.00000 70.00000

109.00000 61.00000

126.00000 54.00000

143.00000 47.00000

160.00000 40.00000

177.00000 34.00000

195.00000 32.00000

214.00000 33.00000

232.00000 35.00000

250.00000 39.00000

268.00000 43.00000

286.00000 47.00000

303.00000 51.00000

322.00000 54.00000

339.00000 59.00000

356.00000 65.00000

373.00000 72.00000

390.00000 80.00000

405.00000 90.00000

421.00000 99.00000

436.00000 110.00000

452.00000 119.00000

467.00000 129.00000

483.00000 139.00000

498.00000 149.00000

514.00000 158.00000

530.00000 167.00000

545.00000 177.00000

560.00000 188.00000

575.00000 199.00000

589.00000 211.00000

604.00000 221.00000

618.00000 232.00000

632.00000 245.00000

645.00000 257.00000

659.00000 269.00000

673.00000 282.00000

683.00000 296.00000

696.00000 309.00000

711.00000 320.00000

723.00000 334.00000

733.00000 349.00000

744.00000 364.00000

746.00000 382.00000

753.00000 399.00000

760.00000 416.00000

766.00000 434.00000

768.00000 452.00000

765.00000 434.00000

IMAGE=20191025LCHR01.jpg

ID=25

SCALE=0.006410

LM=0

CURVES=1

POINTS=100

44.00000 29.00000

62.00000 38.00000

80.00000 48.00000

99.00000 56.00000

117.00000 65.00000

136.00000 72.00000

155.00000 80.00000

173.00000 89.00000

191.00000 99.00000

209.00000 109.00000

227.00000 118.00000

245.00000 128.00000

263.00000 138.00000

280.00000 149.00000

297.00000 160.00000

314.00000 171.00000

331.00000 183.00000

347.00000 196.00000

363.00000 208.00000

378.00000 222.00000

390.00000 238.00000

404.00000 253.00000

416.00000 270.00000

429.00000 286.00000

442.00000 301.00000

453.00000 318.00000

461.00000 337.00000

471.00000 355.00000

483.00000 371.00000

494.00000 389.00000

503.00000 407.00000

514.00000 424.00000

525.00000 441.00000

533.00000 460.00000

540.00000 479.00000

546.00000 498.00000

553.00000 518.00000

558.00000 538.00000

562.00000 557.00000

563.00000 578.00000

564.00000 598.00000

564.00000 619.00000

564.00000 639.00000

562.00000 659.00000

560.00000 680.00000

557.00000 700.00000

553.00000 720.00000

547.00000 739.00000

539.00000 758.00000

530.00000 776.00000

511.00000 783.00000

491.00000 786.00000

471.00000 789.00000

450.00000 788.00000

430.00000 785.00000

411.00000 779.00000

391.00000 772.00000

372.00000 765.00000

353.00000 759.00000

336.00000 748.00000

319.00000 736.00000

303.00000 723.00000

287.00000 711.00000

271.00000 699.00000

256.00000 684.00000

243.00000 669.00000

228.00000 655.00000

213.00000 641.00000

200.00000 626.00000

187.00000 610.00000

174.00000 594.00000

160.00000 579.00000

149.00000 562.00000

139.00000 544.00000

129.00000 527.00000

119.00000 509.00000

108.00000 491.00000

98.00000 474.00000

88.00000 456.00000

82.00000 436.00000

77.00000 417.00000

70.00000 398.00000

63.00000 378.00000

56.00000 359.00000

50.00000 340.00000

45.00000 320.00000

42.00000 300.00000

40.00000 280.00000

38.00000 259.00000

35.00000 239.00000

33.00000 219.00000

31.00000 198.00000

30.00000 178.00000

30.00000 158.00000

30.00000 137.00000

29.00000 117.00000

31.00000 97.00000

27.00000 77.00000

26.00000 56.00000

25.00000 36.00000

IMAGE=20191025LCNP01.jpg

ID=26

SCALE=0.034407

LM=0

CURVES=1

POINTS=100

719.00000 556.00000

739.00000 555.00000

760.00000 552.00000

779.00000 546.00000

796.00000 534.00000

806.00000 517.00000

811.00000 497.00000

816.00000 477.00000

821.00000 457.00000

824.00000 437.00000

824.00000 416.00000

820.00000 396.00000

817.00000 376.00000

819.00000 356.00000

810.00000 338.00000

804.00000 318.00000

794.00000 300.00000

785.00000 282.00000

775.00000 264.00000

764.00000 247.00000

750.00000 232.00000

735.00000 218.00000

720.00000 204.00000

708.00000 187.00000

695.00000 171.00000

681.00000 156.00000

667.00000 141.00000

651.00000 129.00000

635.00000 116.00000

620.00000 102.00000

602.00000 93.00000

584.00000 83.00000

566.00000 74.00000

547.00000 65.00000

528.00000 60.00000

507.00000 57.00000

488.00000 50.00000

468.00000 52.00000

447.00000 50.00000

427.00000 48.00000

406.00000 47.00000

386.00000 49.00000

366.00000 53.00000

346.00000 57.00000

326.00000 57.00000

305.00000 58.00000

285.00000 58.00000

264.00000 59.00000

244.00000 60.00000

223.00000 60.00000

203.00000 62.00000

183.00000 68.00000

163.00000 71.00000

143.00000 76.00000

118.00000 80.00000

112.00000 88.00000

114.00000 103.00000

123.00000 118.00000

124.00000 149.00000

127.00000 169.00000

133.00000 189.00000

137.00000 209.00000

139.00000 229.00000

141.00000 250.00000

144.00000 270.00000

148.00000 290.00000

151.00000 310.00000

155.00000 330.00000

163.00000 349.00000

172.00000 367.00000

181.00000 386.00000

189.00000 404.00000

200.00000 422.00000

211.00000 439.00000

223.00000 455.00000

238.00000 469.00000

255.00000 481.00000

272.00000 492.00000

289.00000 503.00000

304.00000 517.00000

320.00000 529.00000

336.00000 542.00000

355.00000 550.00000

374.00000 557.00000

393.00000 565.00000

413.00000 570.00000

433.00000 574.00000

453.00000 577.00000

473.00000 582.00000

490.00000 593.00000

510.00000 598.00000

530.00000 604.00000

550.00000 606.00000

570.00000 606.00000

591.00000 604.00000

610.00000 598.00000

630.00000 592.00000

649.00000 585.00000

668.00000 578.00000

683.00000 564.00000

IMAGE=20191028LALA01.jpg

ID=27

SCALE=0.020913

LM=0

CURVES=1

POINTS=100

946.00000 175.00000

931.00000 154.00000

922.00000 129.00000

911.00000 105.00000

900.00000 82.00000

880.00000 65.00000

858.00000 50.00000

836.00000 38.00000

811.00000 29.00000

786.00000 20.00000

760.00000 17.00000

734.00000 18.00000

708.00000 19.00000

682.00000 23.00000

657.00000 28.00000

632.00000 37.00000

607.00000 44.00000

583.00000 53.00000

557.00000 59.00000

532.00000 64.00000

508.00000 76.00000

485.00000 87.00000

460.00000 97.00000

436.00000 106.00000

413.00000 118.00000

390.00000 130.00000

366.00000 142.00000

347.00000 158.00000

334.00000 181.00000

322.00000 205.00000

309.00000 227.00000

294.00000 249.00000

278.00000 269.00000

263.00000 290.00000

248.00000 312.00000

234.00000 334.00000

221.00000 357.00000

212.00000 381.00000

202.00000 405.00000

193.00000 430.00000

185.00000 455.00000

177.00000 480.00000

169.00000 505.00000

163.00000 530.00000

156.00000 555.00000

150.00000 580.00000

143.00000 606.00000

137.00000 631.00000

125.00000 654.00000

108.00000 674.00000

91.00000 694.00000

110.00000 689.00000

135.00000 682.00000

161.00000 680.00000

187.00000 680.00000

213.00000 679.00000

239.00000 683.00000

265.00000 684.00000

291.00000 681.00000

317.00000 681.00000

343.00000 680.00000

369.00000 681.00000

395.00000 684.00000

422.00000 683.00000

448.00000 682.00000

474.00000 680.00000

500.00000 682.00000

526.00000 684.00000

552.00000 688.00000

577.00000 681.00000

601.00000 672.00000

623.00000 658.00000

648.00000 649.00000

673.00000 641.00000

698.00000 633.00000

722.00000 624.00000

748.00000 619.00000

772.00000 609.00000

795.00000 597.00000

817.00000 583.00000

841.00000 572.00000

865.00000 561.00000

888.00000 549.00000

909.00000 534.00000

928.00000 516.00000

945.00000 496.00000

961.00000 476.00000

977.00000 455.00000

993.00000 434.00000

1005.00000 411.00000

1018.00000 388.00000

1024.00000 363.00000

1022.00000 336.00000

1019.00000 311.00000

1011.00000 286.00000

1002.00000 261.00000

993.00000 237.00000

983.00000 212.00000

966.00000 192.00000

946.00000 176.00000

IMAGE=20191028LALA02.jpg

ID=28

SCALE=0.017112

LM=0

CURVES=1

POINTS=100

212.00000 10.00000

225.00000 16.00000

239.00000 19.00000

253.00000 23.00000

267.00000 24.00000

282.00000 24.00000

296.00000 25.00000

309.00000 32.00000

322.00000 38.00000

336.00000 43.00000

349.00000 48.00000

363.00000 53.00000

374.00000 62.00000

385.00000 72.00000

395.00000 82.00000

406.00000 92.00000

418.00000 100.00000

430.00000 107.00000

443.00000 114.00000

454.00000 122.00000

466.00000 131.00000

478.00000 140.00000

487.00000 151.00000

496.00000 162.00000

505.00000 173.00000

515.00000 184.00000

527.00000 192.00000

539.00000 199.00000

548.00000 210.00000

558.00000 221.00000

565.00000 234.00000

569.00000 247.00000

580.00000 258.00000

591.00000 266.00000

602.00000 276.00000

610.00000 288.00000

615.00000 301.00000

622.00000 314.00000

628.00000 327.00000

631.00000 341.00000

638.00000 354.00000

642.00000 368.00000

649.00000 381.00000

652.00000 395.00000

655.00000 409.00000

662.00000 421.00000

667.00000 435.00000

678.00000 445.00000

685.00000 457.00000

694.00000 469.00000

702.00000 484.00000

691.00000 484.00000

679.00000 473.00000

666.00000 465.00000

653.00000 459.00000

640.00000 454.00000

625.00000 453.00000

611.00000 450.00000

599.00000 442.00000

587.00000 434.00000

574.00000 427.00000

561.00000 422.00000

548.00000 416.00000

535.00000 409.00000

521.00000 404.00000

508.00000 398.00000

496.00000 391.00000

485.00000 381.00000

476.00000 370.00000

468.00000 358.00000

457.00000 350.00000

446.00000 339.00000

435.00000 330.00000

424.00000 321.00000

413.00000 311.00000

404.00000 300.00000

394.00000 290.00000

380.00000 285.00000

368.00000 277.00000

360.00000 265.00000

350.00000 254.00000

341.00000 243.00000

332.00000 233.00000

322.00000 222.00000

312.00000 211.00000

304.00000 199.00000

295.00000 188.00000

285.00000 177.00000

277.00000 166.00000

268.00000 154.00000

263.00000 141.00000

253.00000 130.00000

246.00000 117.00000

239.00000 105.00000

232.00000 92.00000

225.00000 79.00000

218.00000 67.00000

212.00000 54.00000

207.00000 40.00000

201.00000 27.00000

IMAGE=20191030LUVA01.jpg

ID=29

SCALE=0.020512

LM=0

CURVES=1

POINTS=100

568.00000 565.00000

554.00000 560.00000

539.00000 556.00000

525.00000 551.00000

510.00000 547.00000

496.00000 542.00000

482.00000 536.00000

467.00000 532.00000

453.00000 527.00000

438.00000 522.00000

424.00000 517.00000

411.00000 509.00000

399.00000 500.00000

387.00000 491.00000

375.00000 481.00000

364.00000 471.00000

351.00000 462.00000

340.00000 452.00000

328.00000 443.00000

316.00000 433.00000

305.00000 423.00000

294.00000 413.00000

283.00000 402.00000

272.00000 391.00000

262.00000 380.00000

252.00000 368.00000

244.00000 355.00000

234.00000 344.00000

223.00000 333.00000

213.00000 322.00000

203.00000 310.00000

194.00000 298.00000

185.00000 286.00000

176.00000 274.00000

166.00000 262.00000

157.00000 250.00000

150.00000 237.00000

144.00000 223.00000

141.00000 208.00000

138.00000 193.00000

136.00000 178.00000

134.00000 163.00000

132.00000 148.00000

132.00000 133.00000

131.00000 117.00000

131.00000 102.00000

133.00000 87.00000

127.00000 73.00000

122.00000 59.00000

118.00000 44.00000

115.00000 28.00000

119.00000 24.00000

134.00000 35.00000

147.00000 44.00000

160.00000 51.00000

174.00000 57.00000

188.00000 63.00000

202.00000 68.00000

216.00000 75.00000

230.00000 81.00000

243.00000 88.00000

256.00000 96.00000

269.00000 104.00000

281.00000 113.00000

293.00000 122.00000

306.00000 131.00000

318.00000 140.00000

330.00000 150.00000

341.00000 160.00000

351.00000 171.00000

361.00000 183.00000

371.00000 195.00000

380.00000 206.00000

391.00000 218.00000

401.00000 228.00000

412.00000 239.00000

423.00000 250.00000

432.00000 262.00000

440.00000 275.00000

449.00000 287.00000

458.00000 300.00000

466.00000 312.00000

474.00000 325.00000

483.00000 338.00000

490.00000 351.00000

499.00000 364.00000

507.00000 376.00000

516.00000 389.00000

523.00000 402.00000

530.00000 416.00000

536.00000 429.00000

543.00000 443.00000

549.00000 457.00000

555.00000 471.00000

558.00000 486.00000

561.00000 501.00000

562.00000 516.00000

567.00000 530.00000

569.00000 545.00000

573.00000 560.00000

IMAGE=20191030LUVA02.jpg

ID=30

SCALE=0.020512

LM=0

CURVES=1

POINTS=100

31.00000 175.00000

45.00000 166.00000

58.00000 154.00000

69.00000 141.00000

82.00000 129.00000

95.00000 118.00000

108.00000 108.00000

123.00000 99.00000

139.00000 92.00000

155.00000 87.00000

172.00000 83.00000

188.00000 79.00000

205.00000 75.00000

222.00000 72.00000

239.00000 70.00000

256.00000 67.00000

272.00000 63.00000

289.00000 58.00000

305.00000 53.00000

322.00000 48.00000

338.00000 44.00000

355.00000 42.00000

372.00000 39.00000

389.00000 37.00000

406.00000 36.00000

423.00000 32.00000

440.00000 31.00000

457.00000 30.00000

474.00000 30.00000

491.00000 29.00000

508.00000 32.00000

525.00000 32.00000

542.00000 36.00000

559.00000 36.00000

576.00000 39.00000

593.00000 41.00000

610.00000 45.00000

627.00000 49.00000

641.00000 59.00000

656.00000 66.00000

670.00000 75.00000

685.00000 84.00000

700.00000 92.00000

717.00000 96.00000

733.00000 101.00000

753.00000 115.00000

774.00000 122.00000

797.00000 125.00000

816.00000 121.00000

824.00000 123.00000

821.00000 131.00000

807.00000 139.00000

791.00000 143.00000

775.00000 149.00000

760.00000 157.00000

746.00000 167.00000

730.00000 173.00000

713.00000 177.00000

698.00000 185.00000

683.00000 194.00000

669.00000 203.00000

656.00000 214.00000

639.00000 219.00000

624.00000 226.00000

607.00000 228.00000

594.00000 239.00000

578.00000 241.00000

561.00000 243.00000

547.00000 253.00000

533.00000 264.00000

517.00000 267.00000

500.00000 271.00000

483.00000 272.00000

466.00000 274.00000

449.00000 274.00000

432.00000 275.00000

414.00000 274.00000

397.00000 274.00000

380.00000 273.00000

363.00000 272.00000

346.00000 271.00000

329.00000 270.00000

312.00000 271.00000

295.00000 270.00000

278.00000 268.00000

261.00000 266.00000

244.00000 263.00000

227.00000 260.00000

210.00000 257.00000

193.00000 254.00000

176.00000 251.00000

159.00000 248.00000

143.00000 243.00000

127.00000 238.00000

111.00000 232.00000

95.00000 225.00000

80.00000 217.00000

66.00000 207.00000

52.00000 196.00000

37.00000 189.00000

IMAGE=20191101LUVA01.jpg

ID=31

SCALE=0.006492

LM=0

CURVES=1

POINTS=100

444.00000 43.00000

441.00000 54.00000

436.00000 64.00000

431.00000 74.00000

426.00000 84.00000

421.00000 94.00000

417.00000 105.00000

412.00000 115.00000

408.00000 126.00000

401.00000 135.00000

396.00000 145.00000

390.00000 154.00000

383.00000 163.00000

375.00000 170.00000

365.00000 176.00000

358.00000 185.00000

352.00000 195.00000

346.00000 204.00000

339.00000 213.00000

333.00000 223.00000

326.00000 232.00000

320.00000 241.00000

311.00000 248.00000

302.00000 255.00000

294.00000 263.00000

286.00000 270.00000

277.00000 277.00000

267.00000 284.00000

258.00000 290.00000

249.00000 296.00000

239.00000 303.00000

229.00000 308.00000

219.00000 312.00000

208.00000 316.00000

198.00000 322.00000

187.00000 325.00000

177.00000 328.00000

165.00000 329.00000

154.00000 329.00000

143.00000 328.00000

131.00000 327.00000

121.00000 324.00000

110.00000 320.00000

99.00000 316.00000

88.00000 313.00000

77.00000 312.00000

66.00000 314.00000

56.00000 319.00000

46.00000 323.00000

36.00000 331.00000

24.00000 333.00000

21.00000 325.00000

27.00000 315.00000

36.00000 308.00000

45.00000 301.00000

54.00000 294.00000

59.00000 284.00000

64.00000 274.00000

68.00000 263.00000

70.00000 252.00000

72.00000 241.00000

73.00000 230.00000

74.00000 219.00000

80.00000 209.00000

86.00000 199.00000

94.00000 191.00000

101.00000 183.00000

110.00000 175.00000

118.00000 167.00000

126.00000 159.00000

134.00000 152.00000

143.00000 145.00000

153.00000 139.00000

163.00000 133.00000

172.00000 127.00000

182.00000 121.00000

191.00000 115.00000

200.00000 108.00000

210.00000 103.00000

220.00000 97.00000

230.00000 92.00000

240.00000 87.00000

250.00000 82.00000

260.00000 77.00000

271.00000 73.00000

281.00000 68.00000

292.00000 64.00000

302.00000 61.00000

313.00000 57.00000

324.00000 54.00000

335.00000 51.00000

346.00000 48.00000

356.00000 44.00000

367.00000 41.00000

378.00000 38.00000

389.00000 36.00000

400.00000 35.00000

412.00000 33.00000

423.00000 32.00000

434.00000 30.00000

IMAGE=20191108LBOS01.jpg

ID=32

SCALE=0.006451

LM=0

CURVES=1

POINTS=100

702.00000 375.00000

687.00000 381.00000

672.00000 386.00000

657.00000 390.00000

641.00000 392.00000

625.00000 396.00000

610.00000 395.00000

594.00000 395.00000

578.00000 396.00000

562.00000 397.00000

546.00000 397.00000

530.00000 398.00000

514.00000 398.00000

498.00000 399.00000

482.00000 397.00000

467.00000 393.00000

451.00000 393.00000

435.00000 391.00000

419.00000 388.00000

404.00000 384.00000

389.00000 380.00000

374.00000 374.00000

359.00000 368.00000

344.00000 362.00000

330.00000 356.00000

315.00000 349.00000

301.00000 342.00000

287.00000 335.00000

273.00000 327.00000

259.00000 319.00000

245.00000 311.00000

232.00000 303.00000

218.00000 294.00000

205.00000 285.00000

192.00000 276.00000

180.00000 266.00000

167.00000 256.00000

156.00000 245.00000

146.00000 232.00000

137.00000 219.00000

128.00000 206.00000

119.00000 193.00000

110.00000 180.00000

103.00000 166.00000

97.00000 151.00000

89.00000 137.00000

81.00000 123.00000

77.00000 108.00000

71.00000 94.00000

68.00000 78.00000

75.00000 64.00000

90.00000 59.00000

104.00000 53.00000

120.00000 51.00000

136.00000 47.00000

151.00000 42.00000

167.00000 39.00000

182.00000 39.00000

198.00000 35.00000

213.00000 31.00000

229.00000 27.00000

244.00000 23.00000

260.00000 22.00000

275.00000 26.00000

290.00000 33.00000

306.00000 36.00000

321.00000 40.00000

336.00000 46.00000

350.00000 52.00000

365.00000 58.00000

380.00000 64.00000

395.00000 68.00000

410.00000 75.00000

425.00000 80.00000

436.00000 92.00000

449.00000 100.00000

463.00000 108.00000

478.00000 115.00000

492.00000 121.00000

506.00000 129.00000

520.00000 136.00000

533.00000 146.00000

545.00000 156.00000

557.00000 167.00000

570.00000 177.00000

582.00000 186.00000

596.00000 195.00000

609.00000 204.00000

618.00000 217.00000

628.00000 229.00000

639.00000 240.00000

649.00000 253.00000

659.00000 265.00000

667.00000 279.00000

674.00000 293.00000

684.00000 306.00000

697.00000 316.00000

704.00000 330.00000

709.00000 345.00000

714.00000 360.00000

IMAGE=20191111LCHR01.jpg

ID=33

SCALE=0.006410

LM=0

CURVES=1

POINTS=100

791.00000 382.00000

774.00000 386.00000

757.00000 390.00000

740.00000 393.00000

723.00000 395.00000

706.00000 397.00000

689.00000 399.00000

672.00000 400.00000

654.00000 401.00000

637.00000 404.00000

620.00000 406.00000

603.00000 408.00000

586.00000 405.00000

569.00000 405.00000

552.00000 401.00000

535.00000 399.00000

518.00000 394.00000

501.00000 391.00000

485.00000 385.00000

468.00000 380.00000

451.00000 377.00000

436.00000 370.00000

419.00000 366.00000

402.00000 361.00000

385.00000 358.00000

370.00000 350.00000

353.00000 348.00000

339.00000 339.00000

322.00000 333.00000

307.00000 325.00000

291.00000 320.00000

275.00000 314.00000

259.00000 307.00000

244.00000 298.00000

228.00000 291.00000

214.00000 281.00000

201.00000 270.00000

188.00000 259.00000

177.00000 245.00000

167.00000 231.00000

158.00000 216.00000

149.00000 202.00000

140.00000 187.00000

133.00000 171.00000

126.00000 155.00000

120.00000 139.00000

114.00000 123.00000

107.00000 107.00000

94.00000 96.00000

86.00000 89.00000

89.00000 80.00000

118.00000 78.00000

135.00000 77.00000

152.00000 74.00000

169.00000 69.00000

184.00000 61.00000

200.00000 54.00000

216.00000 47.00000

232.00000 43.00000

250.00000 42.00000

266.00000 38.00000

284.00000 38.00000

301.00000 35.00000

318.00000 34.00000

335.00000 38.00000

350.00000 45.00000

366.00000 53.00000

382.00000 59.00000

398.00000 63.00000

415.00000 67.00000

432.00000 70.00000

449.00000 76.00000

465.00000 80.00000

481.00000 87.00000

498.00000 92.00000

513.00000 99.00000

526.00000 110.00000

540.00000 120.00000

556.00000 128.00000

571.00000 136.00000

586.00000 144.00000

602.00000 152.00000

616.00000 162.00000

629.00000 173.00000

642.00000 184.00000

657.00000 193.00000

671.00000 203.00000

686.00000 212.00000

701.00000 221.00000

715.00000 231.00000

725.00000 244.00000

737.00000 257.00000

748.00000 271.00000

755.00000 286.00000

756.00000 303.00000

762.00000 319.00000

775.00000 332.00000

785.00000 346.00000

796.00000 358.00000

807.00000 372.00000

IMAGE=20191111LCHR01BIS.jpg

ID=34

SCALE=0.006451

LM=0

CURVES=1

POINTS=100

257.00000 241.00000

269.00000 250.00000

282.00000 258.00000

296.00000 265.00000

309.00000 272.00000

324.00000 278.00000

338.00000 284.00000

352.00000 290.00000

367.00000 293.00000

382.00000 297.00000

397.00000 300.00000

412.00000 303.00000

427.00000 304.00000

442.00000 305.00000

457.00000 306.00000

473.00000 305.00000

488.00000 304.00000

503.00000 303.00000

518.00000 300.00000

533.00000 296.00000

548.00000 293.00000

563.00000 292.00000

578.00000 290.00000

594.00000 289.00000

608.00000 284.00000

622.00000 278.00000

636.00000 270.00000

647.00000 260.00000

657.00000 249.00000

669.00000 239.00000

683.00000 233.00000

698.00000 229.00000

712.00000 223.00000

725.00000 216.00000

738.00000 207.00000

749.00000 197.00000

761.00000 187.00000

773.00000 177.00000

784.00000 166.00000

793.00000 154.00000

804.00000 144.00000

816.00000 134.00000

826.00000 123.00000

836.00000 111.00000

845.00000 99.00000

854.00000 86.00000

864.00000 75.00000

873.00000 62.00000

882.00000 50.00000

895.00000 42.00000

910.00000 36.00000

924.00000 31.00000

922.00000 25.00000

907.00000 24.00000

892.00000 24.00000

877.00000 25.00000

861.00000 25.00000

846.00000 26.00000

831.00000 27.00000

816.00000 30.00000

800.00000 31.00000

785.00000 30.00000

770.00000 27.00000

755.00000 23.00000

740.00000 24.00000

725.00000 28.00000

710.00000 31.00000

695.00000 33.00000

680.00000 32.00000

665.00000 32.00000

649.00000 34.00000

635.00000 39.00000

620.00000 44.00000

606.00000 48.00000

591.00000 51.00000

576.00000 56.00000

562.00000 61.00000

548.00000 67.00000

533.00000 72.00000

519.00000 78.00000

504.00000 81.00000

490.00000 87.00000

476.00000 93.00000

461.00000 98.00000

447.00000 104.00000

433.00000 108.00000

419.00000 115.00000

404.00000 119.00000

390.00000 123.00000

375.00000 129.00000

361.00000 135.00000

347.00000 141.00000

336.00000 151.00000

324.00000 160.00000

310.00000 168.00000

297.00000 176.00000

286.00000 186.00000

275.00000 196.00000

267.00000 210.00000

258.00000 222.00000

IMAGE=20191111LUVA01.jpg

ID=35

SCALE=0.020253

LM=0

CURVES=1

POINTS=100

61.00000 24.00000

79.00000 24.00000

96.00000 23.00000

114.00000 22.00000

132.00000 24.00000

149.00000 28.00000

166.00000 33.00000

184.00000 35.00000

201.00000 38.00000

219.00000 41.00000

236.00000 44.00000

253.00000 50.00000

270.00000 55.00000

287.00000 61.00000

302.00000 70.00000

318.00000 77.00000

334.00000 86.00000

350.00000 94.00000

365.00000 102.00000

381.00000 111.00000

397.00000 119.00000

412.00000 127.00000

429.00000 132.00000

446.00000 139.00000

461.00000 148.00000

475.00000 158.00000

489.00000 170.00000

502.00000 181.00000

516.00000 193.00000

530.00000 203.00000

545.00000 213.00000

560.00000 223.00000

574.00000 233.00000

587.00000 245.00000

600.00000 257.00000

612.00000 271.00000

623.00000 285.00000

634.00000 299.00000

644.00000 313.00000

654.00000 328.00000

667.00000 340.00000

680.00000 352.00000

692.00000 365.00000

699.00000 381.00000

705.00000 398.00000

711.00000 415.00000

720.00000 430.00000

728.00000 445.00000

743.00000 454.00000

749.00000 467.00000

743.00000 484.00000

729.00000 491.00000

712.00000 490.00000

694.00000 487.00000

676.00000 484.00000

659.00000 483.00000

641.00000 481.00000

624.00000 477.00000

607.00000 471.00000

591.00000 465.00000

574.00000 458.00000

557.00000 457.00000

539.00000 457.00000

522.00000 451.00000

507.00000 443.00000

491.00000 434.00000

476.00000 425.00000

461.00000 416.00000

444.00000 409.00000

428.00000 403.00000

411.00000 396.00000

395.00000 388.00000

380.00000 380.00000

364.00000 371.00000

350.00000 361.00000

336.00000 350.00000

322.00000 339.00000

309.00000 327.00000

294.00000 317.00000

278.00000 309.00000

264.00000 298.00000

251.00000 286.00000

236.00000 277.00000

223.00000 265.00000

210.00000 253.00000

202.00000 237.00000

192.00000 223.00000

178.00000 212.00000

165.00000 200.00000

150.00000 190.00000

133.00000 183.00000

121.00000 171.00000

109.00000 157.00000

97.00000 144.00000

87.00000 130.00000

78.00000 114.00000

69.00000 99.00000

63.00000 82.00000

57.00000 66.00000

51.00000 49.00000

IMAGE=20191112LUVA02.jpg

ID=36

SCALE=0.006410

LM=0

CURVES=1

POINTS=100

2167.00000 1112.00000

2168.00000 1110.00000

2170.00000 1109.00000

2172.00000 1109.00000

2174.00000 1110.00000

2176.00000 1111.00000

2178.00000 1112.00000

2180.00000 1113.00000

2182.00000 1115.00000

2183.00000 1116.00000

2185.00000 1117.00000

2187.00000 1118.00000

2189.00000 1119.00000

2191.00000 1121.00000

2192.00000 1122.00000

2194.00000 1123.00000

2196.00000 1125.00000

2198.00000 1126.00000

2199.00000 1127.00000

2201.00000 1129.00000

2203.00000 1130.00000

2205.00000 1131.00000

2206.00000 1132.00000

2208.00000 1134.00000

2209.00000 1135.00000

2211.00000 1137.00000

2213.00000 1138.00000

2215.00000 1140.00000

2216.00000 1141.00000

2218.00000 1143.00000

2220.00000 1144.00000

2221.00000 1145.00000

2223.00000 1147.00000

2224.00000 1148.00000

2226.00000 1150.00000

2227.00000 1152.00000

2228.00000 1153.00000

2230.00000 1155.00000

2231.00000 1157.00000

2232.00000 1158.00000

2234.00000 1160.00000

2235.00000 1162.00000

2237.00000 1164.00000

2238.00000 1165.00000

2239.00000 1167.00000

2240.00000 1169.00000

2241.00000 1171.00000

2241.00000 1174.00000

2241.00000 1176.00000

2241.00000 1178.00000

2241.00000 1180.00000

2241.00000 1182.00000

2240.00000 1184.00000

2238.00000 1184.00000

2236.00000 1183.00000

2234.00000 1181.00000

2233.00000 1180.00000

2231.00000 1178.00000

2230.00000 1177.00000

2228.00000 1175.00000

2227.00000 1174.00000

2225.00000 1172.00000

2224.00000 1170.00000

2222.00000 1168.00000

2221.00000 1167.00000

2219.00000 1165.00000

2218.00000 1164.00000

2216.00000 1162.00000

2215.00000 1161.00000

2213.00000 1159.00000

2212.00000 1158.00000

2210.00000 1156.00000

2208.00000 1154.00000

2207.00000 1153.00000

2205.00000 1152.00000

2203.00000 1150.00000

2202.00000 1149.00000

2200.00000 1148.00000

2198.00000 1146.00000

2197.00000 1145.00000

2195.00000 1144.00000

2193.00000 1142.00000

2191.00000 1141.00000

2190.00000 1140.00000

2188.00000 1138.00000

2186.00000 1137.00000

2185.00000 1136.00000

2183.00000 1134.00000

2181.00000 1133.00000

2179.00000 1132.00000

2177.00000 1130.00000

2176.00000 1129.00000

2174.00000 1127.00000

2173.00000 1126.00000

2171.00000 1125.00000

2169.00000 1124.00000

2167.00000 1122.00000

2166.00000 1120.00000

2166.00000 1118.00000

2166.00000 1116.00000

IMAGE=20191113LNEW01.JPG

ID=37

SCALE=0.001289

LM=0

CURVES=1

POINTS=100

2570.00000 958.00000

2621.00000 967.00000

2672.00000 972.00000

2721.00000 988.00000

2766.00000 1014.00000

2810.00000 1040.00000

2855.00000 1066.00000

2899.00000 1092.00000

2940.00000 1123.00000

2978.00000 1158.00000

3013.00000 1196.00000

3048.00000 1234.00000

3081.00000 1273.00000

3114.00000 1312.00000

3151.00000 1348.00000

3188.00000 1385.00000

3226.00000 1420.00000

3262.00000 1456.00000

3291.00000 1499.00000

3318.00000 1543.00000

3346.00000 1586.00000

3369.00000 1632.00000

3384.00000 1681.00000

3401.00000 1730.00000

3421.00000 1777.00000

3440.00000 1825.00000

3454.00000 1875.00000

3468.00000 1924.00000

3487.00000 1972.00000

3513.00000 2016.00000

3539.00000 2061.00000

3556.00000 2109.00000

3571.00000 2159.00000

3586.00000 2208.00000

3594.00000 2259.00000

3594.00000 2310.00000

3589.00000 2361.00000

3582.00000 2412.00000

3575.00000 2464.00000

3571.00000 2515.00000

3569.00000 2566.00000

3573.00000 2618.00000

3583.00000 2668.00000

3587.00000 2719.00000

3575.00000 2769.00000

3556.00000 2816.00000

3530.00000 2861.00000

3498.00000 2900.00000

3464.00000 2940.00000

3427.00000 2975.00000

3381.00000 2978.00000

3332.00000 2962.00000

3282.00000 2949.00000

3232.00000 2938.00000

3181.00000 2930.00000

3131.00000 2919.00000

3082.00000 2901.00000

3038.00000 2876.00000

2993.00000 2850.00000

2947.00000 2826.00000

2905.00000 2797.00000

2868.00000 2761.00000

2835.00000 2722.00000

2804.00000 2681.00000

2777.00000 2637.00000

2753.00000 2591.00000

2729.00000 2546.00000

2702.00000 2502.00000

2677.00000 2457.00000

2653.00000 2411.00000

2629.00000 2366.00000

2606.00000 2319.00000

2588.00000 2271.00000

2571.00000 2223.00000

2553.00000 2174.00000

2536.00000 2126.00000

2519.00000 2077.00000

2502.00000 2028.00000

2487.00000 1979.00000

2476.00000 1929.00000

2464.00000 1879.00000

2451.00000 1829.00000

2436.00000 1779.00000

2418.00000 1731.00000

2404.00000 1682.00000

2390.00000 1632.00000

2380.00000 1581.00000

2372.00000 1530.00000

2372.00000 1479.00000

2374.00000 1427.00000

2367.00000 1376.00000

2371.00000 1325.00000

2386.00000 1276.00000

2392.00000 1225.00000

2405.00000 1175.00000

2427.00000 1128.00000

2447.00000 1081.00000

2468.00000 1034.00000

2490.00000 987.00000

2515.00000 942.00000

IMAGE=20191115LCRP01.JPG

ID=38

SCALE=0.012983

LM=0

CURVES=1

POINTS=100

82.00000 311.00000

66.00000 299.00000

58.00000 281.00000

56.00000 262.00000

59.00000 242.00000

65.00000 223.00000

70.00000 204.00000

78.00000 186.00000

87.00000 169.00000

94.00000 150.00000

105.00000 133.00000

120.00000 121.00000

138.00000 113.00000

156.00000 104.00000

174.00000 96.00000

192.00000 87.00000

210.00000 81.00000

229.00000 75.00000

248.00000 70.00000

267.00000 65.00000

287.00000 60.00000

305.00000 54.00000

325.00000 52.00000

345.00000 52.00000

365.00000 51.00000

383.00000 45.00000

403.00000 43.00000

423.00000 42.00000

442.00000 37.00000

462.00000 38.00000

481.00000 39.00000

501.00000 41.00000

521.00000 44.00000

540.00000 46.00000

560.00000 48.00000

579.00000 52.00000

598.00000 57.00000

615.00000 67.00000

634.00000 73.00000

653.00000 79.00000

672.00000 85.00000

691.00000 91.00000

710.00000 96.00000

727.00000 106.00000

743.00000 117.00000

760.00000 128.00000

777.00000 138.00000

793.00000 149.00000

812.00000 156.00000

830.00000 164.00000

847.00000 174.00000

864.00000 183.00000

896.00000 192.00000

912.00000 196.00000

917.00000 205.00000

915.00000 219.00000

896.00000 227.00000

873.00000 241.00000

855.00000 250.00000

839.00000 261.00000

822.00000 272.00000

805.00000 282.00000

789.00000 293.00000

772.00000 304.00000

756.00000 314.00000

738.00000 323.00000

720.00000 331.00000

701.00000 338.00000

683.00000 346.00000

665.00000 354.00000

647.00000 362.00000

629.00000 369.00000

611.00000 377.00000

592.00000 383.00000

572.00000 386.00000

553.00000 390.00000

534.00000 396.00000

515.00000 400.00000

495.00000 403.00000

476.00000 405.00000

456.00000 406.00000

436.00000 408.00000

417.00000 411.00000

397.00000 412.00000

377.00000 414.00000

358.00000 412.00000

338.00000 411.00000

319.00000 406.00000

300.00000 401.00000

281.00000 395.00000

262.00000 390.00000

243.00000 384.00000

224.00000 378.00000

205.00000 372.00000

187.00000 364.00000

169.00000 357.00000

150.00000 349.00000

135.00000 336.00000

118.00000 327.00000

99.00000 322.00000

IMAGE=20191119LALA01.jpg

ID=39

SCALE=0.020512

LM=0

CURVES=1

POINTS=100

531.00000 95.00000

527.00000 105.00000

523.00000 114.00000

516.00000 122.00000

509.00000 130.00000

502.00000 138.00000

494.00000 144.00000

486.00000 150.00000

477.00000 157.00000

469.00000 163.00000

460.00000 168.00000

450.00000 172.00000

441.00000 176.00000

431.00000 180.00000

421.00000 183.00000

410.00000 184.00000

400.00000 185.00000

390.00000 188.00000

381.00000 192.00000

371.00000 195.00000

361.00000 199.00000

351.00000 201.00000

340.00000 201.00000

330.00000 202.00000

319.00000 202.00000

309.00000 203.00000

299.00000 203.00000

288.00000 204.00000

278.00000 205.00000

268.00000 207.00000

258.00000 209.00000

247.00000 209.00000

237.00000 207.00000

227.00000 205.00000

217.00000 203.00000

206.00000 201.00000

196.00000 198.00000

186.00000 195.00000

176.00000 191.00000

167.00000 186.00000

158.00000 181.00000

149.00000 176.00000

140.00000 172.00000

131.00000 167.00000

122.00000 161.00000

114.00000 154.00000

108.00000 146.00000

101.00000 138.00000

92.00000 133.00000

82.00000 129.00000

68.00000 123.00000

75.00000 119.00000

85.00000 115.00000

95.00000 111.00000

102.00000 103.00000

109.00000 95.00000

116.00000 88.00000

123.00000 81.00000

131.00000 73.00000

137.00000 66.00000

146.00000 60.00000

155.00000 54.00000

163.00000 48.00000

173.00000 45.00000

183.00000 41.00000

192.00000 36.00000

202.00000 34.00000

211.00000 29.00000

222.00000 28.00000

232.00000 27.00000

243.00000 28.00000

253.00000 29.00000

263.00000 29.00000

273.00000 26.00000

284.00000 24.00000

294.00000 23.00000

304.00000 21.00000

315.00000 20.00000

325.00000 20.00000

335.00000 20.00000

346.00000 20.00000

356.00000 21.00000

367.00000 22.00000

377.00000 24.00000

387.00000 25.00000

398.00000 27.00000

408.00000 28.00000

418.00000 31.00000

428.00000 32.00000

438.00000 36.00000

447.00000 41.00000

456.00000 45.00000

466.00000 50.00000

474.00000 56.00000

483.00000 62.00000

492.00000 67.00000

500.00000 74.00000

507.00000 81.00000

515.00000 88.00000

523.00000 95.00000

IMAGE=20191119LBOS01.jpg

ID=40

SCALE=0.020512

LM=0

CURVES=1

POINTS=100

91.00000 111.00000

104.00000 104.00000

117.00000 97.00000

129.00000 90.00000

142.00000 82.00000

154.00000 74.00000

167.00000 67.00000

180.00000 60.00000

193.00000 53.00000

206.00000 46.00000

219.00000 40.00000

234.00000 38.00000

248.00000 35.00000

262.00000 33.00000

276.00000 28.00000

290.00000 23.00000

305.00000 21.00000

319.00000 21.00000

334.00000 22.00000

348.00000 20.00000

363.00000 21.00000

378.00000 21.00000

392.00000 22.00000

407.00000 23.00000

421.00000 23.00000

436.00000 26.00000

451.00000 29.00000

460.00000 39.00000

471.00000 47.00000

485.00000 51.00000

500.00000 53.00000

515.00000 54.00000

529.00000 57.00000

543.00000 62.00000

556.00000 68.00000

569.00000 75.00000

580.00000 84.00000

593.00000 91.00000

603.00000 101.00000

615.00000 110.00000

626.00000 119.00000

636.00000 130.00000

647.00000 140.00000

656.00000 151.00000

663.00000 164.00000

671.00000 177.00000

682.00000 186.00000

695.00000 192.00000

710.00000 193.00000

725.00000 194.00000

736.00000 198.00000

722.00000 203.00000

707.00000 206.00000

693.00000 207.00000

679.00000 210.00000

666.00000 218.00000

654.00000 226.00000

641.00000 233.00000

628.00000 240.00000

616.00000 249.00000

606.00000 259.00000

593.00000 265.00000

580.00000 272.00000

566.00000 276.00000

551.00000 278.00000

537.00000 281.00000

522.00000 283.00000

508.00000 285.00000

493.00000 286.00000

478.00000 287.00000

464.00000 286.00000

449.00000 285.00000

435.00000 285.00000

420.00000 283.00000

406.00000 281.00000

391.00000 279.00000

377.00000 276.00000

362.00000 273.00000

348.00000 271.00000

333.00000 268.00000

319.00000 265.00000

305.00000 261.00000

291.00000 257.00000

277.00000 253.00000

263.00000 248.00000

250.00000 242.00000

236.00000 236.00000

223.00000 230.00000

210.00000 223.00000

196.00000 218.00000

184.00000 210.00000

172.00000 201.00000

161.00000 192.00000

149.00000 183.00000

137.00000 175.00000

125.00000 166.00000

114.00000 157.00000

104.00000 147.00000

94.00000 136.00000

84.00000 125.00000

IMAGE=20191119LBOS02.jpg

ID=41

SCALE=0.006410

LM=0

CURVES=1

POINTS=100

65.00000 52.00000

76.00000 51.00000

86.00000 45.00000

94.00000 37.00000

102.00000 29.00000

111.00000 23.00000

122.00000 20.00000

133.00000 18.00000

145.00000 18.00000

156.00000 19.00000

167.00000 21.00000

178.00000 25.00000

189.00000 24.00000

199.00000 28.00000

210.00000 31.00000

221.00000 35.00000

229.00000 43.00000

239.00000 47.00000

250.00000 51.00000

257.00000 59.00000

267.00000 65.00000

277.00000 70.00000

280.00000 80.00000

286.00000 89.00000

295.00000 96.00000

303.00000 103.00000

312.00000 111.00000

320.00000 119.00000

326.00000 128.00000

334.00000 136.00000

339.00000 146.00000

342.00000 157.00000

349.00000 166.00000

354.00000 175.00000

357.00000 186.00000

363.00000 196.00000

368.00000 206.00000

372.00000 216.00000

377.00000 226.00000

379.00000 237.00000

382.00000 248.00000

386.00000 259.00000

390.00000 269.00000

395.00000 279.00000

401.00000 289.00000

407.00000 299.00000

413.00000 308.00000

419.00000 318.00000

423.00000 328.00000

427.00000 339.00000

422.00000 344.00000

413.00000 339.00000

405.00000 331.00000

396.00000 323.00000

387.00000 318.00000

376.00000 315.00000

365.00000 312.00000

354.00000 313.00000

343.00000 311.00000

332.00000 310.00000

321.00000 314.00000

310.00000 315.00000

299.00000 315.00000

288.00000 314.00000

277.00000 313.00000

266.00000 311.00000

254.00000 310.00000

243.00000 308.00000

232.00000 309.00000

221.00000 305.00000

211.00000 301.00000

200.00000 297.00000

189.00000 294.00000

178.00000 292.00000

169.00000 287.00000

158.00000 285.00000

148.00000 280.00000

137.00000 276.00000

129.00000 268.00000

123.00000 258.00000

112.00000 259.00000

105.00000 251.00000

98.00000 241.00000

91.00000 233.00000

85.00000 224.00000

81.00000 213.00000

78.00000 202.00000

76.00000 191.00000

73.00000 180.00000

70.00000 169.00000

68.00000 158.00000

65.00000 147.00000

62.00000 137.00000

59.00000 126.00000

55.00000 115.00000

57.00000 104.00000

60.00000 93.00000

61.00000 82.00000

60.00000 71.00000

54.00000 61.00000

IMAGE=20191120LCAF01.jpg

ID=42

SCALE=0.020252

LM=0

CURVES=1

POINTS=100

304.00000 23.00000

314.00000 28.00000

321.00000 35.00000

326.00000 45.00000

330.00000 55.00000

334.00000 65.00000

339.00000 74.00000

343.00000 84.00000

345.00000 95.00000

347.00000 105.00000

348.00000 116.00000

349.00000 127.00000

347.00000 137.00000

343.00000 147.00000

340.00000 157.00000

337.00000 168.00000

334.00000 178.00000

331.00000 188.00000

328.00000 199.00000

325.00000 209.00000

319.00000 218.00000

313.00000 226.00000

309.00000 236.00000

301.00000 242.00000

294.00000 251.00000

287.00000 258.00000

279.00000 265.00000

273.00000 274.00000

264.00000 277.00000

258.00000 286.00000

249.00000 292.00000

240.00000 297.00000

230.00000 302.00000

221.00000 308.00000

212.00000 313.00000

204.00000 320.00000

195.00000 325.00000

184.00000 328.00000

175.00000 333.00000

165.00000 337.00000

155.00000 341.00000

145.00000 343.00000

143.00000 336.00000

134.00000 343.00000

125.00000 348.00000

122.00000 344.00000

114.00000 351.00000

106.00000 358.00000

98.00000 365.00000

89.00000 371.00000

79.00000 375.00000

77.00000 367.00000

82.00000 357.00000

88.00000 348.00000

93.00000 339.00000

98.00000 329.00000

104.00000 320.00000

96.00000 318.00000

100.00000 308.00000

104.00000 298.00000

106.00000 288.00000

100.00000 285.00000

101.00000 274.00000

102.00000 264.00000

93.00000 262.00000

95.00000 251.00000

97.00000 242.00000

94.00000 235.00000

95.00000 224.00000

95.00000 214.00000

98.00000 204.00000

96.00000 197.00000

100.00000 187.00000

104.00000 177.00000

100.00000 170.00000

105.00000 161.00000

105.00000 153.00000

107.00000 145.00000

113.00000 136.00000

120.00000 128.00000

125.00000 118.00000

130.00000 109.00000

137.00000 100.00000

144.00000 92.00000

151.00000 85.00000

158.00000 77.00000

166.00000 70.00000

175.00000 63.00000

183.00000 56.00000

192.00000 50.00000

200.00000 43.00000

209.00000 37.00000

218.00000 31.00000

228.00000 28.00000

239.00000 25.00000

249.00000 22.00000

260.00000 21.00000

270.00000 21.00000

281.00000 24.00000

291.00000 27.00000

IMAGE=20191120LCAF02.jpg

ID=43

SCALE=0.020512

LM=0

CURVES=1

POINTS=100

4044.00000 942.00000

4043.00000 941.00000

4042.00000 940.00000

4040.00000 941.00000

4039.00000 942.00000

4038.00000 943.00000

4038.00000 944.00000

4037.00000 945.00000

4036.00000 946.00000

4035.00000 948.00000

4034.00000 949.00000

4033.00000 950.00000

4032.00000 951.00000

4032.00000 953.00000

4031.00000 954.00000

4030.00000 955.00000

4029.00000 956.00000

4029.00000 958.00000

4028.00000 959.00000

4027.00000 960.00000

4026.00000 961.00000

4025.00000 963.00000

4025.00000 964.00000

4024.00000 965.00000

4023.00000 967.00000

4023.00000 968.00000

4022.00000 969.00000

4021.00000 970.00000

4021.00000 972.00000

4021.00000 973.00000

4020.00000 975.00000

4020.00000 976.00000

4019.00000 978.00000

4019.00000 979.00000

4019.00000 981.00000

4018.00000 982.00000

4018.00000 984.00000

4018.00000 985.00000

4018.00000 987.00000

4018.00000 988.00000

4018.00000 990.00000

4018.00000 991.00000

4018.00000 993.00000

4018.00000 994.00000

4018.00000 996.00000

4018.00000 997.00000

4018.00000 999.00000

4018.00000 1001.00000

4018.00000 1002.00000

4018.00000 1004.00000

4019.00000 1005.00000

4019.00000 1006.00000

4021.00000 1005.00000

4021.00000 1004.00000

4022.00000 1003.00000

4022.00000 1001.00000

4023.00000 1000.00000

4023.00000 998.00000

4024.00000 997.00000

4024.00000 995.00000

4025.00000 994.00000

4025.00000 993.00000

4026.00000 991.00000

4027.00000 990.00000

4027.00000 989.00000

4028.00000 988.00000

4029.00000 986.00000

4029.00000 985.00000

4029.00000 984.00000

4030.00000 982.00000

4031.00000 981.00000

4031.00000 980.00000

4032.00000 979.00000

4033.00000 977.00000

4033.00000 976.00000

4034.00000 975.00000

4035.00000 973.00000

4035.00000 972.00000

4036.00000 971.00000

4037.00000 970.00000

4037.00000 968.00000

4038.00000 967.00000

4039.00000 966.00000

4040.00000 965.00000

4041.00000 963.00000

4042.00000 962.00000

4042.00000 961.00000

4043.00000 960.00000

4044.00000 958.00000

4045.00000 957.00000

4045.00000 956.00000

4046.00000 955.00000

4047.00000 954.00000

4048.00000 952.00000

4049.00000 951.00000

4050.00000 950.00000

4049.00000 949.00000

4049.00000 947.00000

4048.00000 946.00000

4047.00000 945.00000

IMAGE=20191120LNEW01.JPG

ID=44

SCALE=0.001607

LM=0

CURVES=1

POINTS=100

720.00000 302.00000

708.00000 313.00000

695.00000 324.00000

682.00000 334.00000

667.00000 342.00000

652.00000 349.00000

636.00000 355.00000

620.00000 359.00000

604.00000 364.00000

588.00000 367.00000

571.00000 370.00000

555.00000 372.00000

538.00000 374.00000

522.00000 374.00000

505.00000 373.00000

488.00000 373.00000

472.00000 372.00000

455.00000 372.00000

438.00000 370.00000

422.00000 367.00000

406.00000 363.00000

390.00000 359.00000

374.00000 353.00000

359.00000 347.00000

343.00000 341.00000

327.00000 335.00000

312.00000 329.00000

296.00000 323.00000

281.00000 317.00000

265.00000 311.00000

250.00000 304.00000

235.00000 298.00000

221.00000 288.00000

208.00000 278.00000

195.00000 268.00000

182.00000 257.00000

169.00000 246.00000

157.00000 235.00000

144.00000 224.00000

133.00000 212.00000

121.00000 201.00000

111.00000 187.00000

102.00000 173.00000

93.00000 159.00000

85.00000 144.00000

77.00000 130.00000

72.00000 114.00000

58.00000 104.00000

44.00000 96.00000

29.00000 89.00000

13.00000 73.00000

12.00000 64.00000

21.00000 61.00000

43.00000 66.00000

59.00000 72.00000

75.00000 76.00000

91.00000 71.00000

106.00000 65.00000

122.00000 59.00000

137.00000 52.00000

153.00000 47.00000

168.00000 41.00000

185.00000 39.00000

202.00000 39.00000

218.00000 38.00000

235.00000 36.00000

251.00000 34.00000

268.00000 35.00000

285.00000 36.00000

301.00000 40.00000

316.00000 46.00000

332.00000 51.00000

348.00000 56.00000

365.00000 56.00000

381.00000 53.00000

397.00000 58.00000

413.00000 63.00000

428.00000 71.00000

443.00000 77.00000

459.00000 84.00000

474.00000 91.00000

489.00000 98.00000

504.00000 105.00000

519.00000 113.00000

534.00000 120.00000

548.00000 129.00000

561.00000 138.00000

575.00000 148.00000

589.00000 158.00000

600.00000 170.00000

612.00000 182.00000

623.00000 194.00000

635.00000 206.00000

647.00000 218.00000

657.00000 230.00000

670.00000 241.00000

682.00000 253.00000

693.00000 265.00000

707.00000 275.00000

720.00000 285.00000

IMAGE=20191126LBOS01.jpg

ID=45

SCALE=0.004761

LM=0

CURVES=1

POINTS=100

735.00000 409.00000

720.00000 417.00000

705.00000 424.00000

690.00000 432.00000

673.00000 435.00000

657.00000 439.00000

640.00000 442.00000

623.00000 444.00000

606.00000 447.00000

590.00000 449.00000

573.00000 449.00000

556.00000 449.00000

539.00000 448.00000

522.00000 446.00000

505.00000 444.00000

488.00000 441.00000

472.00000 438.00000

455.00000 435.00000

439.00000 431.00000

423.00000 426.00000

408.00000 418.00000

393.00000 410.00000

378.00000 402.00000

363.00000 393.00000

348.00000 385.00000

333.00000 378.00000

318.00000 370.00000

304.00000 361.00000

290.00000 352.00000

276.00000 341.00000

261.00000 333.00000

246.00000 327.00000

234.00000 315.00000

225.00000 301.00000

211.00000 291.00000

197.00000 282.00000

183.00000 272.00000

169.00000 263.00000

157.00000 251.00000

150.00000 236.00000

142.00000 221.00000

133.00000 206.00000

124.00000 192.00000

117.00000 176.00000

112.00000 160.00000

109.00000 143.00000

107.00000 127.00000

114.00000 106.00000

108.00000 96.00000

98.00000 82.00000

87.00000 69.00000

84.00000 62.00000

99.00000 62.00000

118.00000 69.00000

139.00000 58.00000

151.00000 46.00000

167.00000 40.00000

184.00000 37.00000

200.00000 32.00000

216.00000 26.00000

232.00000 22.00000

249.00000 20.00000

266.00000 22.00000

283.00000 24.00000

299.00000 27.00000

316.00000 30.00000

332.00000 34.00000

349.00000 39.00000

365.00000 44.00000

381.00000 49.00000

397.00000 54.00000

413.00000 61.00000

428.00000 69.00000

442.00000 78.00000

455.00000 88.00000

468.00000 99.00000

482.00000 110.00000

495.00000 120.00000

508.00000 131.00000

521.00000 142.00000

534.00000 153.00000

546.00000 164.00000

558.00000 176.00000

570.00000 188.00000

583.00000 200.00000

594.00000 213.00000

605.00000 226.00000

615.00000 239.00000

626.00000 252.00000

637.00000 265.00000

648.00000 278.00000

659.00000 291.00000

668.00000 305.00000

677.00000 319.00000

686.00000 334.00000

696.00000 348.00000

705.00000 362.00000

714.00000 376.00000

725.00000 389.00000

736.00000 402.00000

IMAGE=20191126LBOS01BIS.jpg

ID=46

SCALE=0.004739

LM=0

CURVES=1

POINTS=100

143.00000 100.00000

153.00000 98.00000

162.00000 95.00000

171.00000 90.00000

179.00000 85.00000

188.00000 79.00000

196.00000 73.00000

204.00000 67.00000

212.00000 61.00000

220.00000 55.00000

229.00000 51.00000

238.00000 47.00000

247.00000 44.00000

256.00000 38.00000

265.00000 34.00000

274.00000 29.00000

283.00000 25.00000

292.00000 22.00000

301.00000 18.00000

311.00000 15.00000

321.00000 13.00000

331.00000 11.00000

341.00000 11.00000

350.00000 9.00000

360.00000 7.00000

370.00000 7.00000

380.00000 6.00000

390.00000 5.00000

400.00000 5.00000

410.00000 5.00000

420.00000 4.00000

430.00000 4.00000

440.00000 3.00000

450.00000 3.00000

460.00000 3.00000

470.00000 3.00000

480.00000 4.00000

490.00000 5.00000

499.00000 9.00000

508.00000 14.00000

516.00000 19.00000

525.00000 24.00000

534.00000 29.00000

543.00000 32.00000

553.00000 35.00000

562.00000 38.00000

572.00000 41.00000

581.00000 44.00000

594.00000 46.00000

603.00000 49.00000

605.00000 54.00000

601.00000 59.00000

588.00000 65.00000

578.00000 68.00000

569.00000 71.00000

560.00000 76.00000

554.00000 84.00000

548.00000 92.00000

542.00000 100.00000

533.00000 105.00000

525.00000 111.00000

517.00000 116.00000

508.00000 121.00000

499.00000 126.00000

491.00000 131.00000

482.00000 136.00000

473.00000 141.00000

464.00000 145.00000

454.00000 147.00000

445.00000 149.00000

435.00000 151.00000

425.00000 154.00000

415.00000 156.00000

406.00000 159.00000

396.00000 161.00000

387.00000 164.00000

377.00000 165.00000

367.00000 165.00000

357.00000 164.00000

347.00000 163.00000

337.00000 162.00000

327.00000 161.00000

317.00000 160.00000

307.00000 159.00000

297.00000 158.00000

287.00000 156.00000

277.00000 154.00000

268.00000 152.00000

258.00000 149.00000

249.00000 146.00000

239.00000 142.00000

230.00000 139.00000

221.00000 135.00000

211.00000 131.00000

202.00000 127.00000

193.00000 123.00000

184.00000 119.00000

175.00000 114.00000

166.00000 110.00000

157.00000 106.00000

IMAGE=2021101LTEC02.jpg

ID=48

SCALE=0.030289

LM=0

CURVES=1

POINTS=100

40.00000 32.00000

66.00000 31.00000

92.00000 30.00000

117.00000 30.00000

143.00000 29.00000

169.00000 29.00000

195.00000 28.00000

220.00000 30.00000

246.00000 35.00000

271.00000 40.00000

295.00000 49.00000

319.00000 59.00000

343.00000 69.00000

367.00000 79.00000

389.00000 91.00000

412.00000 103.00000

437.00000 111.00000

459.00000 124.00000

479.00000 141.00000

501.00000 155.00000

522.00000 170.00000

543.00000 184.00000

564.00000 199.00000

584.00000 215.00000

605.00000 230.00000

626.00000 245.00000

647.00000 261.00000

667.00000 277.00000

685.00000 296.00000

703.00000 314.00000

720.00000 333.00000

738.00000 352.00000

750.00000 375.00000

765.00000 395.00000

782.00000 415.00000

798.00000 435.00000

815.00000 455.00000

830.00000 475.00000

845.00000 497.00000

857.00000 519.00000

866.00000 543.00000

871.00000 569.00000

877.00000 594.00000

884.00000 619.00000

891.00000 644.00000

898.00000 668.00000

901.00000 694.00000

915.00000 715.00000

932.00000 735.00000

948.00000 755.00000

960.00000 778.00000

941.00000 772.00000

918.00000 759.00000

897.00000 744.00000

874.00000 732.00000

850.00000 723.00000

825.00000 718.00000

799.00000 714.00000

773.00000 714.00000

748.00000 717.00000

722.00000 716.00000

697.00000 710.00000

672.00000 702.00000

648.00000 695.00000

623.00000 687.00000

599.00000 678.00000

575.00000 668.00000

553.00000 654.00000

531.00000 641.00000

509.00000 627.00000

487.00000 615.00000

467.00000 598.00000

444.00000 585.00000

423.00000 571.00000

406.00000 552.00000

385.00000 537.00000

363.00000 524.00000

342.00000 509.00000

325.00000 489.00000

305.00000 473.00000

284.00000 458.00000

266.00000 439.00000

248.00000 421.00000

231.00000 402.00000

212.00000 384.00000

195.00000 364.00000

180.00000 343.00000

163.00000 324.00000

149.00000 302.00000

135.00000 281.00000

120.00000 260.00000

107.00000 237.00000

96.00000 214.00000

86.00000 190.00000

80.00000 165.00000

74.00000 140.00000

69.00000 115.00000

62.00000 90.00000

47.00000 69.00000

32.00000 48.00000

IMAGE=20211029LLNC01.jpg

ID=49

SCALE=0.020407

LM=0

CURVES=1

POINTS=100

134.00000 78.00000

153.00000 72.00000

171.00000 66.00000

191.00000 62.00000

210.00000 60.00000

230.00000 60.00000

249.00000 60.00000

269.00000 61.00000

288.00000 63.00000

308.00000 66.00000

327.00000 66.00000

347.00000 67.00000

366.00000 69.00000

385.00000 74.00000

404.00000 80.00000

423.00000 85.00000

441.00000 92.00000

458.00000 101.00000

476.00000 110.00000

493.00000 119.00000

510.00000 128.00000

527.00000 138.00000

544.00000 148.00000

561.00000 158.00000

577.00000 170.00000

592.00000 182.00000

606.00000 195.00000

621.00000 208.00000

637.00000 220.00000

653.00000 231.00000

669.00000 243.00000

685.00000 254.00000

700.00000 266.00000

715.00000 279.00000

728.00000 293.00000

741.00000 308.00000

752.00000 324.00000

763.00000 340.00000

774.00000 357.00000

785.00000 373.00000

795.00000 390.00000

805.00000 407.00000

815.00000 424.00000

824.00000 441.00000

834.00000 458.00000

843.00000 476.00000

853.00000 492.00000

865.00000 508.00000

885.00000 528.00000

902.00000 544.00000

904.00000 551.00000

875.00000 537.00000

857.00000 529.00000

839.00000 523.00000

819.00000 520.00000

800.00000 521.00000

781.00000 523.00000

761.00000 523.00000

741.00000 523.00000

722.00000 520.00000

703.00000 516.00000

684.00000 511.00000

665.00000 506.00000

645.00000 503.00000

626.00000 499.00000

607.00000 495.00000

588.00000 491.00000

569.00000 488.00000

550.00000 483.00000

531.00000 479.00000

513.00000 471.00000

495.00000 463.00000

476.00000 456.00000

458.00000 450.00000

440.00000 442.00000

422.00000 434.00000

405.00000 425.00000

387.00000 416.00000

370.00000 407.00000

354.00000 396.00000

338.00000 384.00000

323.00000 371.00000

308.00000 359.00000

292.00000 347.00000

277.00000 335.00000

262.00000 322.00000

249.00000 307.00000

236.00000 293.00000

223.00000 278.00000

212.00000 262.00000

199.00000 247.00000

189.00000 231.00000

178.00000 214.00000

167.00000 198.00000

157.00000 181.00000

149.00000 163.00000

142.00000 145.00000

138.00000 126.00000

136.00000 106.00000

129.00000 88.00000

IMAGE=20211029LLNC02.jpg

ID=50

SCALE=0.020407

LM=0

CURVES=1

POINTS=100

76.00000 104.00000

89.00000 92.00000

104.00000 81.00000

119.00000 72.00000

135.00000 63.00000

151.00000 55.00000

168.00000 47.00000

185.00000 42.00000

203.00000 40.00000

221.00000 40.00000

239.00000 39.00000

256.00000 34.00000

272.00000 41.00000

289.00000 48.00000

307.00000 51.00000

323.00000 60.00000

339.00000 67.00000

356.00000 74.00000

372.00000 82.00000

388.00000 89.00000

405.00000 96.00000

420.00000 105.00000

438.00000 110.00000

452.00000 121.00000

467.00000 131.00000

482.00000 141.00000

498.00000 150.00000

513.00000 160.00000

526.00000 173.00000

539.00000 185.00000

549.00000 200.00000

559.00000 215.00000

569.00000 230.00000

581.00000 243.00000

592.00000 258.00000

603.00000 272.00000

615.00000 286.00000

623.00000 302.00000

630.00000 318.00000

635.00000 336.00000

642.00000 352.00000

646.00000 370.00000

651.00000 387.00000

656.00000 405.00000

662.00000 422.00000

668.00000 439.00000

674.00000 455.00000

684.00000 470.00000

695.00000 485.00000

706.00000 499.00000

716.00000 514.00000

725.00000 529.00000

738.00000 545.00000

725.00000 540.00000

710.00000 528.00000

696.00000 518.00000

680.00000 509.00000

663.00000 502.00000

646.00000 498.00000

628.00000 499.00000

610.00000 498.00000

592.00000 498.00000

574.00000 496.00000

556.00000 493.00000

538.00000 490.00000

520.00000 488.00000

502.00000 487.00000

485.00000 483.00000

467.00000 479.00000

450.00000 474.00000

432.00000 469.00000

415.00000 467.00000

397.00000 465.00000

379.00000 460.00000

363.00000 453.00000

346.00000 446.00000

330.00000 439.00000

314.00000 430.00000

299.00000 420.00000

284.00000 410.00000

270.00000 399.00000

255.00000 388.00000

240.00000 379.00000

225.00000 368.00000

210.00000 358.00000

198.00000 345.00000

184.00000 333.00000

172.00000 321.00000

160.00000 307.00000

148.00000 293.00000

138.00000 278.00000

128.00000 264.00000

118.00000 248.00000

113.00000 231.00000

108.00000 214.00000

101.00000 197.00000

91.00000 182.00000

81.00000 167.00000

75.00000 150.00000

70.00000 133.00000

IMAGE=20211103LCDU01.jpg

ID=51

SCALE=0.020617

LM=0

CURVES=1

POINTS=100

65.00000 184.00000

73.00000 169.00000

81.00000 154.00000

91.00000 140.00000

100.00000 125.00000

112.00000 112.00000

124.00000 99.00000

137.00000 88.00000

150.00000 77.00000

165.00000 69.00000

179.00000 59.00000

194.00000 50.00000

210.00000 43.00000

226.00000 37.00000

243.00000 34.00000

260.00000 33.00000

277.00000 30.00000

294.00000 28.00000

311.00000 26.00000

328.00000 24.00000

346.00000 22.00000

363.00000 20.00000

380.00000 19.00000

397.00000 18.00000

414.00000 17.00000

432.00000 17.00000

449.00000 17.00000

466.00000 17.00000

483.00000 19.00000

500.00000 19.00000

518.00000 21.00000

535.00000 22.00000

552.00000 23.00000

569.00000 24.00000

586.00000 27.00000

603.00000 32.00000

620.00000 36.00000

636.00000 41.00000

652.00000 47.00000

668.00000 53.00000

685.00000 59.00000

700.00000 66.00000

716.00000 74.00000

732.00000 80.00000

748.00000 85.00000

765.00000 89.00000

782.00000 91.00000

799.00000 92.00000

817.00000 92.00000

833.00000 87.00000

849.00000 80.00000

853.00000 85.00000

839.00000 95.00000

825.00000 105.00000

811.00000 115.00000

797.00000 125.00000

783.00000 135.00000

769.00000 146.00000

754.00000 155.00000

740.00000 164.00000

725.00000 173.00000

710.00000 181.00000

694.00000 188.00000

679.00000 196.00000

662.00000 202.00000

646.00000 207.00000

629.00000 212.00000

613.00000 217.00000

597.00000 223.00000

580.00000 228.00000

563.00000 231.00000

547.00000 237.00000

531.00000 242.00000

514.00000 247.00000

497.00000 250.00000

480.00000 254.00000

463.00000 257.00000

446.00000 260.00000

429.00000 262.00000

412.00000 263.00000

395.00000 264.00000

377.00000 264.00000

360.00000 265.00000

343.00000 266.00000

326.00000 266.00000

309.00000 267.00000

291.00000 267.00000

274.00000 266.00000

257.00000 264.00000

240.00000 261.00000

223.00000 258.00000

206.00000 254.00000

190.00000 248.00000

174.00000 243.00000

157.00000 237.00000

141.00000 232.00000

125.00000 225.00000

111.00000 216.00000

96.00000 206.00000

81.00000 198.00000

IMAGE=20211103LCDU02.jpg

ID=52

SCALE=0.020617

LM=0

CURVES=1

POINTS=100

1900.00000 1350.00000

1900.00000 1351.00000

1900.00000 1353.00000

1900.00000 1354.00000

1902.00000 1355.00000

1903.00000 1355.00000

1904.00000 1356.00000

1905.00000 1356.00000

1907.00000 1357.00000

1908.00000 1357.00000

1909.00000 1358.00000

1911.00000 1358.00000

1912.00000 1359.00000

1913.00000 1359.00000

1915.00000 1360.00000

1916.00000 1360.00000

1917.00000 1361.00000

1919.00000 1362.00000

1920.00000 1362.00000

1921.00000 1363.00000

1922.00000 1363.00000

1924.00000 1363.00000

1925.00000 1364.00000

1927.00000 1364.00000

1928.00000 1364.00000

1929.00000 1365.00000

1931.00000 1365.00000

1933.00000 1366.00000

1934.00000 1366.00000

1935.00000 1366.00000

1936.00000 1367.00000

1937.00000 1367.00000

1939.00000 1368.00000

1940.00000 1368.00000

1941.00000 1368.00000

1943.00000 1369.00000

1944.00000 1369.00000

1945.00000 1369.00000

1946.00000 1369.00000

1948.00000 1370.00000

1950.00000 1370.00000

1951.00000 1370.00000

1953.00000 1370.00000

1954.00000 1370.00000

1955.00000 1369.00000

1957.00000 1369.00000

1958.00000 1368.00000

1959.00000 1368.00000

1961.00000 1367.00000

1962.00000 1366.00000

1963.00000 1365.00000

1964.00000 1364.00000

1965.00000 1363.00000

1964.00000 1363.00000

1963.00000 1362.00000

1962.00000 1362.00000

1960.00000 1361.00000

1959.00000 1361.00000

1958.00000 1360.00000

1956.00000 1359.00000

1955.00000 1359.00000

1954.00000 1358.00000

1952.00000 1358.00000

1951.00000 1357.00000

1950.00000 1357.00000

1948.00000 1357.00000

1947.00000 1356.00000

1946.00000 1356.00000

1944.00000 1355.00000

1943.00000 1355.00000

1942.00000 1355.00000

1940.00000 1354.00000

1939.00000 1354.00000

1937.00000 1353.00000

1936.00000 1353.00000

1935.00000 1353.00000

1933.00000 1352.00000

1932.00000 1352.00000

1931.00000 1351.00000

1929.00000 1351.00000

1928.00000 1351.00000

1927.00000 1350.00000

1925.00000 1350.00000

1924.00000 1350.00000

1923.00000 1349.00000

1921.00000 1349.00000

1920.00000 1348.00000

1918.00000 1348.00000

1917.00000 1348.00000

1915.00000 1347.00000

1914.00000 1347.00000

1913.00000 1347.00000

1912.00000 1346.00000

1910.00000 1346.00000

1909.00000 1346.00000

1907.00000 1346.00000

1906.00000 1346.00000

1905.00000 1346.00000

1903.00000 1347.00000

1902.00000 1347.00000

IMAGE=20211103LNEW01.jpg

ID=53

SCALE=0.001697

LM=0

CURVES=1

POINTS=100

143.00000 225.00000

133.00000 208.00000

136.00000 188.00000

142.00000 169.00000

150.00000 151.00000

156.00000 132.00000

168.00000 116.00000

184.00000 103.00000

197.00000 89.00000

213.00000 77.00000

231.00000 68.00000

246.00000 54.00000

262.00000 42.00000

279.00000 32.00000

297.00000 24.00000

315.00000 16.00000

334.00000 9.00000

354.00000 5.00000

373.00000 3.00000

393.00000 2.00000

413.00000 2.00000

433.00000 6.00000

452.00000 10.00000

472.00000 11.00000

492.00000 11.00000

512.00000 10.00000

532.00000 9.00000

552.00000 11.00000

572.00000 13.00000

591.00000 17.00000

611.00000 21.00000

630.00000 26.00000

650.00000 30.00000

669.00000 35.00000

688.00000 41.00000

707.00000 47.00000

726.00000 54.00000

745.00000 60.00000

764.00000 66.00000

782.00000 74.00000

801.00000 82.00000

816.00000 94.00000

833.00000 105.00000

849.00000 117.00000

866.00000 128.00000

883.00000 138.00000

898.00000 152.00000

912.00000 165.00000

928.00000 177.00000

959.00000 193.00000

971.00000 202.00000

962.00000 204.00000

941.00000 211.00000

924.00000 221.00000

907.00000 232.00000

892.00000 246.00000

878.00000 260.00000

862.00000 271.00000

846.00000 283.00000

830.00000 295.00000

814.00000 306.00000

797.00000 317.00000

781.00000 329.00000

764.00000 339.00000

746.00000 348.00000

729.00000 358.00000

710.00000 365.00000

691.00000 372.00000

671.00000 375.00000

652.00000 378.00000

632.00000 381.00000

612.00000 385.00000

593.00000 388.00000

573.00000 386.00000

553.00000 390.00000

533.00000 392.00000

513.00000 392.00000

494.00000 390.00000

474.00000 386.00000

454.00000 388.00000

434.00000 387.00000

414.00000 385.00000

395.00000 389.00000

375.00000 390.00000

355.00000 391.00000

335.00000 391.00000

315.00000 388.00000

297.00000 380.00000

279.00000 371.00000

262.00000 360.00000

246.00000 349.00000

230.00000 337.00000

210.00000 333.00000

192.00000 327.00000

174.00000 316.00000

162.00000 301.00000

151.00000 284.00000

140.00000 260.00000

136.00000 241.00000

144.00000 231.00000

IMAGE=20211104LCTN01.jpg

ID=54

SCALE=0.020407

LM=0

CURVES=1

POINTS=100

76.00000 103.00000

91.00000 112.00000

105.00000 124.00000

119.00000 136.00000

134.00000 147.00000

148.00000 158.00000

161.00000 171.00000

174.00000 183.00000

189.00000 193.00000

206.00000 199.00000

224.00000 205.00000

240.00000 212.00000

258.00000 217.00000

275.00000 223.00000

292.00000 229.00000

309.00000 236.00000

326.00000 241.00000

344.00000 244.00000

361.00000 239.00000

378.00000 234.00000

396.00000 236.00000

414.00000 239.00000

432.00000 242.00000

450.00000 241.00000

468.00000 237.00000

485.00000 234.00000

504.00000 234.00000

522.00000 234.00000

539.00000 229.00000

557.00000 226.00000

574.00000 220.00000

591.00000 213.00000

607.00000 204.00000

624.00000 199.00000

642.00000 196.00000

660.00000 196.00000

677.00000 190.00000

694.00000 182.00000

708.00000 172.00000

724.00000 163.00000

742.00000 158.00000

759.00000 152.00000

775.00000 145.00000

792.00000 138.00000

809.00000 132.00000

826.00000 126.00000

844.00000 121.00000

861.00000 116.00000

879.00000 111.00000

897.00000 111.00000

921.00000 107.00000

911.00000 106.00000

893.00000 106.00000

875.00000 106.00000

857.00000 106.00000

839.00000 104.00000

821.00000 101.00000

804.00000 95.00000

788.00000 86.00000

772.00000 78.00000

754.00000 73.00000

739.00000 64.00000

722.00000 57.00000

705.00000 51.00000

688.00000 46.00000

670.00000 42.00000

652.00000 39.00000

635.00000 32.00000

618.00000 28.00000

599.00000 26.00000

581.00000 25.00000

563.00000 23.00000

545.00000 21.00000

527.00000 21.00000

509.00000 24.00000

491.00000 22.00000

473.00000 23.00000

455.00000 25.00000

437.00000 22.00000

419.00000 23.00000

401.00000 24.00000

383.00000 27.00000

365.00000 29.00000

347.00000 32.00000

329.00000 33.00000

311.00000 34.00000

293.00000 34.00000

275.00000 35.00000

257.00000 36.00000

239.00000 39.00000

221.00000 40.00000

203.00000 40.00000

185.00000 46.00000

169.00000 53.00000

152.00000 59.00000

134.00000 61.00000

118.00000 70.00000

100.00000 74.00000

83.00000 80.00000

67.00000 88.00000

IMAGE=20211104LOLW01.jpg

ID=55

SCALE=0.020201

LM=0

CURVES=1

POINTS=100

104.00000 149.00000

115.00000 162.00000

126.00000 174.00000

141.00000 182.00000

153.00000 193.00000

167.00000 203.00000

181.00000 211.00000

196.00000 219.00000

210.00000 228.00000

225.00000 235.00000

241.00000 240.00000

258.00000 242.00000

274.00000 244.00000

291.00000 247.00000

307.00000 251.00000

322.00000 259.00000

338.00000 263.00000

355.00000 263.00000

371.00000 264.00000

388.00000 263.00000

404.00000 258.00000

421.00000 259.00000

437.00000 256.00000

454.00000 256.00000

470.00000 257.00000

487.00000 258.00000

504.00000 256.00000

518.00000 248.00000

533.00000 241.00000

550.00000 237.00000

566.00000 235.00000

583.00000 233.00000

599.00000 228.00000

614.00000 223.00000

630.00000 216.00000

646.00000 210.00000

658.00000 200.00000

672.00000 190.00000

688.00000 184.00000

704.00000 182.00000

720.00000 176.00000

734.00000 167.00000

748.00000 158.00000

764.00000 153.00000

780.00000 147.00000

795.00000 141.00000

811.00000 135.00000

826.00000 128.00000

842.00000 123.00000

855.00000 114.00000

873.00000 109.00000

870.00000 103.00000

845.00000 101.00000

828.00000 100.00000

811.00000 99.00000

795.00000 96.00000

778.00000 94.00000

762.00000 89.00000

747.00000 84.00000

731.00000 78.00000

715.00000 73.00000

699.00000 68.00000

684.00000 60.00000

670.00000 51.00000

654.00000 46.00000

639.00000 39.00000

623.00000 36.00000

607.00000 32.00000

591.00000 25.00000

576.00000 20.00000

559.00000 20.00000

542.00000 20.00000

526.00000 17.00000

512.00000 8.00000

495.00000 6.00000

479.00000 10.00000

462.00000 10.00000

446.00000 10.00000

429.00000 9.00000

412.00000 11.00000

396.00000 13.00000

379.00000 16.00000

363.00000 20.00000

347.00000 22.00000

330.00000 22.00000

314.00000 25.00000

297.00000 29.00000

281.00000 34.00000

265.00000 39.00000

250.00000 45.00000

237.00000 55.00000

221.00000 61.00000

206.00000 68.00000

192.00000 77.00000

178.00000 87.00000

165.00000 97.00000

151.00000 107.00000

139.00000 118.00000

126.00000 128.00000

113.00000 139.00000

IMAGE=20211104LOLW02.jpg

ID=56

SCALE=0.010204

LM=0

CURVES=1

POINTS=100

1793.00000 1292.00000

1769.00000 1286.00000

1746.00000 1277.00000

1721.00000 1276.00000

1697.00000 1282.00000

1674.00000 1293.00000

1652.00000 1305.00000

1633.00000 1321.00000

1616.00000 1339.00000

1601.00000 1359.00000

1586.00000 1379.00000

1573.00000 1400.00000

1561.00000 1422.00000

1549.00000 1443.00000

1538.00000 1466.00000

1524.00000 1486.00000

1512.00000 1508.00000

1502.00000 1531.00000

1493.00000 1554.00000

1486.00000 1578.00000

1477.00000 1601.00000

1465.00000 1623.00000

1456.00000 1646.00000

1451.00000 1670.00000

1445.00000 1695.00000

1435.00000 1717.00000

1429.00000 1741.00000

1424.00000 1766.00000

1421.00000 1790.00000

1414.00000 1814.00000

1407.00000 1838.00000

1399.00000 1862.00000

1392.00000 1886.00000

1392.00000 1910.00000

1394.00000 1935.00000

1391.00000 1960.00000

1385.00000 1984.00000

1379.00000 2008.00000

1374.00000 2032.00000

1377.00000 2057.00000

1381.00000 2081.00000

1384.00000 2106.00000

1384.00000 2131.00000

1385.00000 2156.00000

1384.00000 2181.00000

1380.00000 2205.00000

1376.00000 2230.00000

1374.00000 2255.00000

1376.00000 2279.00000

1381.00000 2304.00000

1394.00000 2325.00000

1415.00000 2338.00000

1436.00000 2352.00000

1452.00000 2363.00000

1470.00000 2348.00000

1488.00000 2332.00000

1502.00000 2311.00000

1516.00000 2291.00000

1529.00000 2269.00000

1541.00000 2248.00000

1551.00000 2225.00000

1562.00000 2202.00000

1571.00000 2179.00000

1580.00000 2156.00000

1587.00000 2133.00000

1594.00000 2109.00000

1602.00000 2085.00000

1606.00000 2060.00000

1611.00000 2036.00000

1616.00000 2012.00000

1623.00000 1988.00000

1628.00000 1963.00000

1633.00000 1939.00000

1639.00000 1915.00000

1642.00000 1890.00000

1645.00000 1866.00000

1652.00000 1842.00000

1660.00000 1818.00000

1669.00000 1795.00000

1678.00000 1772.00000

1685.00000 1748.00000

1686.00000 1723.00000

1687.00000 1698.00000

1695.00000 1675.00000

1708.00000 1653.00000

1718.00000 1630.00000

1723.00000 1606.00000

1729.00000 1582.00000

1742.00000 1561.00000

1754.00000 1539.00000

1768.00000 1519.00000

1782.00000 1498.00000

1792.00000 1475.00000

1802.00000 1452.00000

1812.00000 1429.00000

1818.00000 1405.00000

1821.00000 1381.00000

1818.00000 1356.00000

1815.00000 1331.00000

1806.00000 1308.00000

IMAGE=20211112LTUA01.jpg

ID=57

SCALE=0.021642

LM=0

CURVES=1

POINTS=100

1676.00000 1300.00000

1683.00000 1273.00000

1685.00000 1245.00000

1687.00000 1217.00000

1690.00000 1189.00000

1690.00000 1161.00000

1683.00000 1134.00000

1678.00000 1106.00000

1672.00000 1079.00000

1666.00000 1052.00000

1662.00000 1024.00000

1656.00000 997.00000

1649.00000 969.00000

1641.00000 943.00000

1631.00000 916.00000

1620.00000 891.00000

1610.00000 864.00000

1600.00000 838.00000

1590.00000 812.00000

1579.00000 786.00000

1569.00000 760.00000

1557.00000 734.00000

1546.00000 709.00000

1536.00000 683.00000

1524.00000 657.00000

1513.00000 631.00000

1502.00000 606.00000

1491.00000 580.00000

1477.00000 556.00000

1461.00000 533.00000

1449.00000 507.00000

1436.00000 483.00000

1422.00000 458.00000

1406.00000 435.00000

1391.00000 412.00000

1376.00000 388.00000

1357.00000 367.00000

1338.00000 346.00000

1318.00000 327.00000

1297.00000 308.00000

1276.00000 290.00000

1256.00000 270.00000

1235.00000 252.00000

1215.00000 233.00000

1194.00000 214.00000

1172.00000 196.00000

1151.00000 178.00000

1129.00000 160.00000

1107.00000 142.00000

1102.00000 115.00000

1101.00000 129.00000

1100.00000 157.00000

1098.00000 185.00000

1097.00000 213.00000

1099.00000 241.00000

1100.00000 269.00000

1103.00000 297.00000

1109.00000 324.00000

1116.00000 351.00000

1126.00000 377.00000

1136.00000 403.00000

1147.00000 429.00000

1159.00000 455.00000

1169.00000 481.00000

1183.00000 505.00000

1196.00000 530.00000

1208.00000 555.00000

1221.00000 580.00000

1232.00000 606.00000

1244.00000 631.00000

1254.00000 657.00000

1264.00000 683.00000

1275.00000 709.00000

1285.00000 735.00000

1299.00000 760.00000

1313.00000 784.00000

1324.00000 810.00000

1336.00000 835.00000

1347.00000 861.00000

1360.00000 886.00000

1372.00000 911.00000

1381.00000 938.00000

1390.00000 964.00000

1399.00000 991.00000

1410.00000 1017.00000

1421.00000 1042.00000

1431.00000 1069.00000

1442.00000 1094.00000

1452.00000 1121.00000

1464.00000 1146.00000

1476.00000 1171.00000

1490.00000 1195.00000

1505.00000 1219.00000

1521.00000 1242.00000

1539.00000 1264.00000

1559.00000 1282.00000

1583.00000 1298.00000

1609.00000 1309.00000

1636.00000 1314.00000

1664.00000 1311.00000

IMAGE=20211112LTUA02.jpg

ID=58

SCALE=0.022220

LM=0

CURVES=1

POINTS=100

2592.00000 1481.00000

2561.00000 1466.00000

2529.00000 1453.00000

2497.00000 1441.00000

2465.00000 1429.00000

2433.00000 1417.00000

2401.00000 1403.00000

2370.00000 1390.00000

2338.00000 1376.00000

2308.00000 1360.00000

2278.00000 1343.00000

2250.00000 1323.00000

2222.00000 1303.00000

2193.00000 1285.00000

2162.00000 1270.00000

2136.00000 1247.00000

2109.00000 1226.00000

2079.00000 1209.00000

2053.00000 1188.00000

2023.00000 1171.00000

1994.00000 1152.00000

1965.00000 1133.00000

1939.00000 1111.00000

1913.00000 1088.00000

1888.00000 1066.00000

1861.00000 1044.00000

1835.00000 1021.00000

1810.00000 998.00000

1783.00000 977.00000

1760.00000 952.00000

1734.00000 929.00000

1713.00000 902.00000

1695.00000 873.00000

1674.00000 846.00000

1654.00000 818.00000

1636.00000 788.00000

1619.00000 758.00000

1598.00000 732.00000

1584.00000 700.00000

1568.00000 670.00000

1555.00000 638.00000

1547.00000 605.00000

1536.00000 572.00000

1526.00000 539.00000

1524.00000 505.00000

1520.00000 471.00000

1517.00000 437.00000

1504.00000 405.00000

1458.00000 353.00000

1455.00000 337.00000

1467.00000 331.00000

1497.00000 348.00000

1529.00000 361.00000

1561.00000 375.00000

1593.00000 385.00000

1626.00000 395.00000

1659.00000 403.00000

1693.00000 411.00000

1725.00000 422.00000

1757.00000 435.00000

1788.00000 450.00000

1818.00000 467.00000

1849.00000 482.00000

1879.00000 498.00000

1910.00000 514.00000

1940.00000 530.00000

1967.00000 551.00000

1994.00000 572.00000

2018.00000 597.00000

2045.00000 618.00000

2070.00000 642.00000

2094.00000 665.00000

2119.00000 689.00000

2144.00000 713.00000

2169.00000 737.00000

2193.00000 761.00000

2216.00000 787.00000

2237.00000 814.00000

2259.00000 840.00000

2285.00000 863.00000

2308.00000 888.00000

2329.00000 915.00000

2349.00000 943.00000

2368.00000 972.00000

2387.00000 1000.00000

2406.00000 1029.00000

2424.00000 1058.00000

2443.00000 1087.00000

2462.00000 1115.00000

2479.00000 1145.00000

2492.00000 1177.00000

2506.00000 1208.00000

2518.00000 1240.00000

2528.00000 1273.00000

2541.00000 1305.00000

2552.00000 1338.00000

2563.00000 1370.00000

2573.00000 1403.00000

2584.00000 1436.00000

2595.00000 1468.00000

IMAGE=20211117LAPH01.jpg

ID=59

SCALE=0.012016

LM=0

CURVES=1

POINTS=100

47.00000 136.00000

60.00000 124.00000

73.00000 112.00000

86.00000 100.00000

100.00000 88.00000

114.00000 77.00000

128.00000 67.00000

143.00000 58.00000

159.00000 50.00000

176.00000 44.00000

193.00000 39.00000

210.00000 36.00000

227.00000 29.00000

244.00000 26.00000

261.00000 21.00000

279.00000 18.00000

296.00000 21.00000

314.00000 24.00000

332.00000 24.00000

349.00000 25.00000

367.00000 27.00000

385.00000 26.00000

402.00000 25.00000

420.00000 24.00000

438.00000 24.00000

456.00000 25.00000

473.00000 26.00000

491.00000 27.00000

509.00000 28.00000

527.00000 28.00000

544.00000 32.00000

561.00000 35.00000

579.00000 38.00000

596.00000 41.00000

614.00000 45.00000

631.00000 49.00000

648.00000 53.00000

664.00000 60.00000

680.00000 69.00000

696.00000 77.00000

710.00000 87.00000

728.00000 91.00000

744.00000 97.00000

758.00000 108.00000

774.00000 116.00000

790.00000 124.00000

807.00000 129.00000

824.00000 132.00000

842.00000 135.00000

854.00000 140.00000

838.00000 149.00000

822.00000 156.00000

806.00000 164.00000

791.00000 174.00000

777.00000 185.00000

764.00000 197.00000

750.00000 208.00000

736.00000 218.00000

720.00000 226.00000

705.00000 235.00000

689.00000 245.00000

674.00000 254.00000

659.00000 262.00000

642.00000 269.00000

626.00000 276.00000

609.00000 281.00000

592.00000 286.00000

575.00000 290.00000

557.00000 294.00000

540.00000 296.00000

522.00000 297.00000

504.00000 299.00000

487.00000 300.00000

469.00000 302.00000

451.00000 305.00000

434.00000 307.00000

416.00000 308.00000

398.00000 308.00000

381.00000 304.00000

364.00000 299.00000

346.00000 298.00000

329.00000 296.00000

311.00000 294.00000

293.00000 292.00000

276.00000 291.00000

258.00000 289.00000

241.00000 285.00000

224.00000 280.00000

207.00000 275.00000

190.00000 269.00000

174.00000 261.00000

157.00000 255.00000

142.00000 246.00000

126.00000 238.00000

110.00000 229.00000

97.00000 217.00000

88.00000 203.00000

76.00000 189.00000

63.00000 177.00000

50.00000 165.00000

IMAGE=20211119LCDU01.jpg

ID=60

SCALE=0.020407

LM=0

CURVES=1

POINTS=100

763.00000 125.00000

751.00000 134.00000

739.00000 145.00000

729.00000 156.00000

718.00000 167.00000

706.00000 178.00000

695.00000 189.00000

683.00000 198.00000

671.00000 208.00000

657.00000 213.00000

641.00000 216.00000

627.00000 222.00000

612.00000 227.00000

597.00000 230.00000

582.00000 233.00000

567.00000 236.00000

552.00000 239.00000

536.00000 242.00000

522.00000 247.00000

508.00000 253.00000

493.00000 259.00000

479.00000 265.00000

465.00000 270.00000

450.00000 276.00000

435.00000 276.00000

419.00000 277.00000

404.00000 278.00000

389.00000 282.00000

374.00000 286.00000

358.00000 288.00000

343.00000 288.00000

327.00000 287.00000

312.00000 286.00000

297.00000 283.00000

282.00000 279.00000

266.00000 280.00000

251.00000 278.00000

236.00000 277.00000

220.00000 276.00000

205.00000 272.00000

191.00000 267.00000

177.00000 260.00000

162.00000 257.00000

146.00000 254.00000

131.00000 252.00000

116.00000 250.00000

100.00000 248.00000

85.00000 245.00000

70.00000 242.00000

55.00000 241.00000

75.00000 226.00000

88.00000 218.00000

101.00000 211.00000

115.00000 203.00000

128.00000 196.00000

142.00000 187.00000

154.00000 179.00000

166.00000 169.00000

178.00000 159.00000

190.00000 149.00000

201.00000 138.00000

212.00000 128.00000

226.00000 121.00000

240.00000 114.00000

254.00000 108.00000

268.00000 101.00000

281.00000 93.00000

295.00000 85.00000

308.00000 77.00000

322.00000 71.00000

336.00000 64.00000

351.00000 59.00000

366.00000 55.00000

381.00000 51.00000

396.00000 48.00000

411.00000 45.00000

426.00000 42.00000

441.00000 38.00000

456.00000 36.00000

472.00000 35.00000

487.00000 33.00000

503.00000 31.00000

518.00000 30.00000

534.00000 29.00000

549.00000 29.00000

564.00000 28.00000

580.00000 29.00000

595.00000 34.00000

609.00000 39.00000

625.00000 41.00000

640.00000 43.00000

655.00000 48.00000

670.00000 52.00000

684.00000 57.00000

699.00000 62.00000

713.00000 69.00000

726.00000 77.00000

739.00000 85.00000

749.00000 97.00000

761.00000 107.00000

IMAGE=20211119LDYO01.jpg

ID=61

SCALE=0.020617

LM=0

CURVES=1

POINTS=100

3039.00000 1454.00000

3048.00000 1475.00000

3056.00000 1496.00000

3064.00000 1517.00000

3070.00000 1538.00000

3074.00000 1561.00000

3076.00000 1583.00000

3074.00000 1606.00000

3072.00000 1628.00000

3068.00000 1650.00000

3063.00000 1672.00000

3058.00000 1694.00000

3051.00000 1715.00000

3047.00000 1738.00000

3042.00000 1760.00000

3036.00000 1781.00000

3028.00000 1802.00000

3019.00000 1823.00000

3013.00000 1845.00000

3005.00000 1866.00000

2994.00000 1886.00000

2982.00000 1905.00000

2974.00000 1925.00000

2964.00000 1946.00000

2952.00000 1965.00000

2939.00000 1984.00000

2926.00000 2001.00000

2914.00000 2021.00000

2903.00000 2040.00000

2894.00000 2061.00000

2882.00000 2080.00000

2872.00000 2100.00000

2860.00000 2119.00000

2849.00000 2139.00000

2839.00000 2159.00000

2830.00000 2180.00000

2818.00000 2199.00000

2806.00000 2218.00000

2794.00000 2237.00000

2780.00000 2255.00000

2766.00000 2273.00000

2750.00000 2288.00000

2732.00000 2301.00000

2714.00000 2315.00000

2694.00000 2326.00000

2651.00000 2335.00000

2616.00000 2336.00000

2594.00000 2339.00000

2582.00000 2353.00000

2573.00000 2355.00000

2568.00000 2348.00000

2568.00000 2332.00000

2572.00000 2317.00000

2569.00000 2303.00000

2558.00000 2275.00000

2555.00000 2250.00000

2555.00000 2216.00000

2557.00000 2194.00000

2559.00000 2171.00000

2563.00000 2149.00000

2570.00000 2128.00000

2577.00000 2106.00000

2583.00000 2084.00000

2588.00000 2062.00000

2594.00000 2041.00000

2605.00000 2021.00000

2617.00000 2002.00000

2628.00000 1982.00000

2637.00000 1962.00000

2646.00000 1941.00000

2653.00000 1920.00000

2662.00000 1899.00000

2671.00000 1879.00000

2681.00000 1858.00000

2692.00000 1839.00000

2701.00000 1818.00000

2711.00000 1798.00000

2722.00000 1778.00000

2732.00000 1758.00000

2740.00000 1737.00000

2750.00000 1716.00000

2759.00000 1696.00000

2768.00000 1675.00000

2778.00000 1655.00000

2788.00000 1635.00000

2798.00000 1615.00000

2808.00000 1595.00000

2818.00000 1575.00000

2830.00000 1555.00000

2841.00000 1536.00000

2854.00000 1517.00000

2869.00000 1501.00000

2885.00000 1485.00000

2903.00000 1471.00000

2921.00000 1458.00000

2942.00000 1449.00000

2963.00000 1442.00000

2986.00000 1439.00000

3008.00000 1438.00000

3030.00000 1433.00000

IMAGE=20211119LTUA01.jpg

ID=62

SCALE=0.020617

LM=0

CURVES=1

POINTS=100

62.00000 195.00000

76.00000 184.00000

90.00000 174.00000

105.00000 164.00000

120.00000 155.00000

136.00000 146.00000

151.00000 137.00000

167.00000 129.00000

182.00000 121.00000

199.00000 114.00000

214.00000 106.00000

230.00000 98.00000

246.00000 91.00000

262.00000 83.00000

278.00000 75.00000

294.00000 68.00000

311.00000 62.00000

328.00000 56.00000

345.00000 55.00000

363.00000 54.00000

381.00000 52.00000

398.00000 49.00000

415.00000 44.00000

432.00000 41.00000

450.00000 40.00000

468.00000 38.00000

485.00000 35.00000

503.00000 33.00000

520.00000 33.00000

538.00000 33.00000

556.00000 33.00000

573.00000 33.00000

591.00000 33.00000

609.00000 34.00000

626.00000 34.00000

643.00000 39.00000

660.00000 44.00000

677.00000 49.00000

694.00000 54.00000

711.00000 59.00000

728.00000 64.00000

745.00000 70.00000

760.00000 78.00000

772.00000 91.00000

784.00000 105.00000

799.00000 114.00000

815.00000 120.00000

832.00000 125.00000

850.00000 127.00000

867.00000 130.00000

852.00000 135.00000

836.00000 142.00000

821.00000 151.00000

806.00000 162.00000

794.00000 175.00000

785.00000 190.00000

775.00000 204.00000

762.00000 216.00000

747.00000 226.00000

732.00000 236.00000

718.00000 245.00000

702.00000 254.00000

687.00000 263.00000

671.00000 270.00000

654.00000 275.00000

637.00000 280.00000

620.00000 285.00000

603.00000 290.00000

586.00000 296.00000

569.00000 300.00000

552.00000 303.00000

534.00000 306.00000

517.00000 308.00000

499.00000 310.00000

481.00000 312.00000

464.00000 314.00000

446.00000 315.00000

429.00000 312.00000

411.00000 310.00000

393.00000 310.00000

376.00000 309.00000

358.00000 310.00000

340.00000 310.00000

323.00000 308.00000

305.00000 305.00000

289.00000 299.00000

272.00000 295.00000

254.00000 293.00000

237.00000 289.00000

220.00000 283.00000

203.00000 277.00000

186.00000 272.00000

170.00000 266.00000

153.00000 260.00000

136.00000 254.00000

120.00000 248.00000

103.00000 242.00000

88.00000 233.00000

73.00000 224.00000

58.00000 214.00000

IMAGE=20211122LLNC01.jpg

ID=63

SCALE=0.010309

LM=0

CURVES=1

POINTS=100

114.00000 162.00000

109.00000 146.00000

112.00000 129.00000

120.00000 115.00000

133.00000 104.00000

146.00000 94.00000

160.00000 84.00000

174.00000 75.00000

189.00000 67.00000

204.00000 61.00000

221.00000 57.00000

237.00000 52.00000

253.00000 48.00000

270.00000 45.00000

286.00000 44.00000

303.00000 43.00000

320.00000 40.00000

336.00000 36.00000

353.00000 34.00000

369.00000 32.00000

386.00000 30.00000

403.00000 31.00000

419.00000 33.00000

436.00000 35.00000

453.00000 38.00000

469.00000 39.00000

486.00000 41.00000

503.00000 43.00000

519.00000 44.00000

536.00000 46.00000

553.00000 48.00000

569.00000 51.00000

586.00000 54.00000

602.00000 58.00000

618.00000 64.00000

634.00000 70.00000

649.00000 76.00000

664.00000 85.00000

678.00000 94.00000

692.00000 103.00000

705.00000 113.00000

721.00000 120.00000

735.00000 128.00000

748.00000 139.00000

764.00000 145.00000

780.00000 151.00000

795.00000 157.00000

812.00000 161.00000

827.00000 167.00000

842.00000 174.00000

848.00000 179.00000

818.00000 182.00000

801.00000 184.00000

784.00000 186.00000

768.00000 190.00000

753.00000 197.00000

741.00000 209.00000

730.00000 221.00000

716.00000 231.00000

702.00000 240.00000

688.00000 249.00000

674.00000 259.00000

661.00000 270.00000

647.00000 279.00000

633.00000 288.00000

618.00000 296.00000

603.00000 303.00000

588.00000 310.00000

572.00000 315.00000

555.00000 319.00000

539.00000 322.00000

523.00000 327.00000

508.00000 334.00000

491.00000 338.00000

475.00000 340.00000

458.00000 341.00000

441.00000 339.00000

425.00000 337.00000

408.00000 335.00000

392.00000 331.00000

375.00000 328.00000

359.00000 323.00000

342.00000 320.00000

326.00000 317.00000

309.00000 314.00000

293.00000 312.00000

276.00000 309.00000

260.00000 304.00000

244.00000 299.00000

229.00000 293.00000

213.00000 287.00000

198.00000 280.00000

184.00000 270.00000

171.00000 260.00000

158.00000 249.00000

144.00000 239.00000

131.00000 229.00000

122.00000 215.00000

117.00000 199.00000

119.00000 182.00000

IMAGE=20211123LNST01.jpg

ID=65

SCALE=0.020407

LM=0

CURVES=1

POINTS=100

48.00000 116.00000

57.00000 102.00000

66.00000 88.00000

78.00000 77.00000

93.00000 71.00000

106.00000 61.00000

121.00000 53.00000

136.00000 46.00000

151.00000 41.00000

168.00000 38.00000

184.00000 36.00000

200.00000 34.00000

217.00000 33.00000

233.00000 35.00000

250.00000 36.00000

266.00000 35.00000

283.00000 34.00000

299.00000 35.00000

316.00000 36.00000

332.00000 38.00000

348.00000 39.00000

365.00000 39.00000

381.00000 41.00000

397.00000 44.00000

414.00000 48.00000

430.00000 50.00000

446.00000 51.00000

463.00000 54.00000

479.00000 57.00000

495.00000 59.00000

511.00000 63.00000

527.00000 68.00000

542.00000 74.00000

558.00000 79.00000

574.00000 80.00000

590.00000 84.00000

606.00000 88.00000

622.00000 93.00000

638.00000 98.00000

653.00000 105.00000

669.00000 108.00000

685.00000 111.00000

701.00000 115.00000

718.00000 116.00000

734.00000 121.00000

749.00000 126.00000

765.00000 131.00000

781.00000 135.00000

797.00000 138.00000

812.00000 146.00000

827.00000 152.00000

836.00000 161.00000

821.00000 156.00000

806.00000 151.00000

789.00000 150.00000

773.00000 149.00000

756.00000 150.00000

740.00000 153.00000

724.00000 155.00000

708.00000 158.00000

691.00000 159.00000

675.00000 161.00000

658.00000 163.00000

642.00000 163.00000

625.00000 164.00000

609.00000 164.00000

593.00000 166.00000

576.00000 168.00000

560.00000 169.00000

543.00000 170.00000

527.00000 171.00000

510.00000 171.00000

494.00000 171.00000

477.00000 171.00000

461.00000 171.00000

444.00000 171.00000

428.00000 170.00000

411.00000 170.00000

395.00000 171.00000

378.00000 172.00000

362.00000 172.00000

345.00000 172.00000

329.00000 173.00000

312.00000 173.00000

296.00000 173.00000

279.00000 173.00000

263.00000 172.00000

246.00000 172.00000

230.00000 172.00000

214.00000 171.00000

197.00000 171.00000

181.00000 167.00000

165.00000 165.00000

148.00000 162.00000

133.00000 157.00000

116.00000 154.00000

101.00000 149.00000

86.00000 142.00000

71.00000 134.00000

56.00000 128.00000

IMAGE=20211124LEUC01.jpg

ID=66

SCALE=0.020407

LM=0

CURVES=1

POINTS=100

758.00000 69.00000

752.00000 84.00000

746.00000 98.00000

738.00000 112.00000

733.00000 127.00000

726.00000 141.00000

720.00000 156.00000

713.00000 170.00000

703.00000 182.00000

692.00000 193.00000

679.00000 202.00000

665.00000 209.00000

650.00000 215.00000

635.00000 219.00000

620.00000 224.00000

605.00000 227.00000

589.00000 231.00000

574.00000 233.00000

558.00000 233.00000

542.00000 232.00000

527.00000 232.00000

511.00000 233.00000

495.00000 233.00000

479.00000 234.00000

464.00000 236.00000

448.00000 235.00000

432.00000 234.00000

417.00000 236.00000

401.00000 237.00000

385.00000 238.00000

370.00000 235.00000

354.00000 231.00000

339.00000 228.00000

323.00000 227.00000

307.00000 227.00000

292.00000 228.00000

276.00000 224.00000

261.00000 222.00000

245.00000 220.00000

230.00000 216.00000

215.00000 211.00000

200.00000 207.00000

185.00000 202.00000

170.00000 196.00000

155.00000 190.00000

141.00000 184.00000

126.00000 179.00000

111.00000 174.00000

96.00000 168.00000

81.00000 164.00000

67.00000 158.00000

53.00000 150.00000

37.00000 148.00000

31.00000 143.00000

46.00000 138.00000

61.00000 132.00000

76.00000 129.00000

92.00000 128.00000

107.00000 124.00000

122.00000 119.00000

138.00000 117.00000

153.00000 115.00000

169.00000 111.00000

183.00000 106.00000

198.00000 101.00000

214.00000 98.00000

229.00000 94.00000

244.00000 90.00000

259.00000 85.00000

275.00000 81.00000

290.00000 77.00000

305.00000 73.00000

320.00000 68.00000

335.00000 64.00000

350.00000 59.00000

366.00000 55.00000

381.00000 51.00000

396.00000 47.00000

412.00000 45.00000

427.00000 43.00000

443.00000 40.00000

458.00000 37.00000

474.00000 34.00000

490.00000 33.00000

505.00000 31.00000

520.00000 27.00000

536.00000 27.00000

552.00000 26.00000

568.00000 26.00000

583.00000 27.00000

599.00000 28.00000

615.00000 28.00000

631.00000 29.00000

646.00000 34.00000

661.00000 38.00000

675.00000 46.00000

689.00000 53.00000

704.00000 58.00000

719.00000 60.00000

735.00000 60.00000

IMAGE=20211124LEUC02.jpg

ID=67

SCALE=0.020407

LM=0

CURVES=1

POINTS=100

38.00000 93.00000

44.00000 110.00000

50.00000 126.00000

57.00000 143.00000

67.00000 158.00000

78.00000 171.00000

89.00000 185.00000

101.00000 198.00000

113.00000 211.00000

128.00000 221.00000

143.00000 230.00000

160.00000 237.00000

177.00000 241.00000

194.00000 247.00000

210.00000 253.00000

228.00000 257.00000

245.00000 259.00000

263.00000 259.00000

281.00000 261.00000

298.00000 263.00000

316.00000 265.00000

334.00000 264.00000

351.00000 263.00000

369.00000 260.00000

387.00000 258.00000

404.00000 253.00000

421.00000 249.00000

439.00000 247.00000

456.00000 244.00000

473.00000 239.00000

490.00000 235.00000

508.00000 231.00000

525.00000 227.00000

542.00000 223.00000

559.00000 218.00000

576.00000 213.00000

593.00000 207.00000

610.00000 201.00000

627.00000 195.00000

644.00000 190.00000

661.00000 185.00000

678.00000 179.00000

694.00000 174.00000

711.00000 168.00000

727.00000 160.00000

743.00000 153.00000

758.00000 143.00000

772.00000 132.00000

785.00000 120.00000

799.00000 109.00000

813.00000 98.00000

849.00000 65.00000

852.00000 58.00000

849.00000 57.00000

839.00000 62.00000

825.00000 71.00000

808.00000 78.00000

791.00000 84.00000

774.00000 88.00000

756.00000 88.00000

739.00000 86.00000

721.00000 85.00000

703.00000 85.00000

686.00000 85.00000

668.00000 84.00000

650.00000 83.00000

632.00000 83.00000

615.00000 82.00000

597.00000 81.00000

579.00000 79.00000

562.00000 77.00000

544.00000 74.00000

527.00000 79.00000

509.00000 79.00000

492.00000 77.00000

474.00000 75.00000

456.00000 73.00000

439.00000 70.00000

421.00000 68.00000

403.00000 67.00000

386.00000 65.00000

368.00000 63.00000

350.00000 62.00000

333.00000 61.00000

315.00000 58.00000

297.00000 56.00000

280.00000 56.00000

262.00000 56.00000

244.00000 56.00000

226.00000 57.00000

209.00000 57.00000

191.00000 58.00000

173.00000 59.00000

155.00000 60.00000

138.00000 62.00000

121.00000 68.00000

105.00000 76.00000

89.00000 83.00000

72.00000 87.00000

54.00000 85.00000

IMAGE=20211124LEUC03.jpg

ID=68

SCALE=0.020407

LM=0

CURVES=1

POINTS=100

81.00000 256.00000

73.00000 240.00000

69.00000 222.00000

69.00000 204.00000

75.00000 187.00000

80.00000 169.00000

86.00000 152.00000

94.00000 136.00000

102.00000 120.00000

111.00000 104.00000

120.00000 88.00000

132.00000 75.00000

146.00000 63.00000

162.00000 54.00000

177.00000 44.00000

193.00000 36.00000

211.00000 31.00000

229.00000 30.00000

247.00000 27.00000

264.00000 23.00000

282.00000 21.00000

301.00000 20.00000

319.00000 20.00000

337.00000 20.00000

355.00000 18.00000

373.00000 17.00000

391.00000 20.00000

408.00000 25.00000

426.00000 30.00000

443.00000 35.00000

461.00000 39.00000

477.00000 47.00000

493.00000 56.00000

509.00000 64.00000

525.00000 73.00000

541.00000 82.00000

557.00000 90.00000

573.00000 100.00000

586.00000 111.00000

601.00000 123.00000

614.00000 134.00000

628.00000 146.00000

642.00000 158.00000

654.00000 171.00000

665.00000 186.00000

677.00000 199.00000

691.00000 212.00000

704.00000 223.00000

719.00000 235.00000

730.00000 249.00000

744.00000 261.00000

759.00000 271.00000

775.00000 277.00000

777.00000 289.00000

772.00000 293.00000

737.00000 290.00000

719.00000 293.00000

702.00000 300.00000

686.00000 309.00000

670.00000 316.00000

653.00000 322.00000

636.00000 329.00000

619.00000 336.00000

602.00000 342.00000

586.00000 351.00000

571.00000 361.00000

556.00000 372.00000

541.00000 382.00000

526.00000 391.00000

509.00000 399.00000

493.00000 407.00000

477.00000 415.00000

459.00000 420.00000

442.00000 425.00000

424.00000 430.00000

407.00000 434.00000

389.00000 436.00000

371.00000 438.00000

353.00000 440.00000

335.00000 443.00000

317.00000 446.00000

299.00000 448.00000

281.00000 448.00000

263.00000 447.00000

245.00000 444.00000

227.00000 440.00000

210.00000 433.00000

194.00000 425.00000

178.00000 417.00000

163.00000 407.00000

146.00000 399.00000

131.00000 390.00000

116.00000 379.00000

103.00000 366.00000

91.00000 353.00000

81.00000 338.00000

74.00000 321.00000

70.00000 303.00000

72.00000 285.00000

82.00000 270.00000

IMAGE=20211125LCTN01.jpg

ID=69

SCALE=0.020546

LM=0

CURVES=1

POINTS=100

124.00000 184.00000

136.00000 172.00000

146.00000 159.00000

159.00000 147.00000

172.00000 136.00000

184.00000 124.00000

196.00000 112.00000

207.00000 100.00000

220.00000 89.00000

234.00000 80.00000

250.00000 73.00000

266.00000 69.00000

283.00000 66.00000

299.00000 60.00000

315.00000 55.00000

331.00000 51.00000

348.00000 47.00000

364.00000 43.00000

381.00000 39.00000

397.00000 34.00000

414.00000 32.00000

431.00000 32.00000

448.00000 31.00000

464.00000 32.00000

481.00000 34.00000

498.00000 36.00000

515.00000 39.00000

532.00000 40.00000

549.00000 39.00000

565.00000 39.00000

582.00000 41.00000

599.00000 45.00000

615.00000 51.00000

630.00000 57.00000

646.00000 64.00000

662.00000 70.00000

678.00000 75.00000

693.00000 82.00000

708.00000 91.00000

722.00000 99.00000

737.00000 108.00000

751.00000 117.00000

766.00000 125.00000

781.00000 134.00000

795.00000 142.00000

810.00000 150.00000

826.00000 156.00000

843.00000 160.00000

878.00000 165.00000

890.00000 172.00000

888.00000 175.00000

872.00000 181.00000

856.00000 185.00000

839.00000 185.00000

822.00000 187.00000

806.00000 193.00000

791.00000 201.00000

777.00000 211.00000

763.00000 219.00000

749.00000 229.00000

735.00000 240.00000

722.00000 250.00000

708.00000 259.00000

692.00000 266.00000

677.00000 272.00000

661.00000 279.00000

645.00000 284.00000

629.00000 290.00000

613.00000 295.00000

597.00000 300.00000

581.00000 305.00000

564.00000 310.00000

548.00000 314.00000

531.00000 316.00000

514.00000 317.00000

498.00000 319.00000

481.00000 320.00000

464.00000 319.00000

447.00000 319.00000

430.00000 318.00000

413.00000 318.00000

396.00000 317.00000

379.00000 314.00000

363.00000 311.00000

346.00000 307.00000

330.00000 304.00000

313.00000 299.00000

297.00000 294.00000

282.00000 288.00000

266.00000 281.00000

250.00000 275.00000

235.00000 268.00000

219.00000 261.00000

205.00000 253.00000

191.00000 243.00000

177.00000 233.00000

163.00000 223.00000

149.00000 214.00000

135.00000 204.00000

121.00000 195.00000

IMAGE=20211125LOLW01.jpg

ID=70

SCALE=0.020617

LM=0

CURVES=1

POINTS=100

37.00000 204.00000

41.00000 223.00000

48.00000 240.00000

56.00000 258.00000

67.00000 273.00000

79.00000 287.00000

92.00000 302.00000

106.00000 314.00000

123.00000 323.00000

141.00000 330.00000

159.00000 336.00000

176.00000 343.00000

195.00000 347.00000

213.00000 351.00000

231.00000 357.00000

250.00000 362.00000

269.00000 363.00000

288.00000 363.00000

306.00000 366.00000

325.00000 369.00000

344.00000 371.00000

363.00000 372.00000

382.00000 373.00000

401.00000 374.00000

420.00000 374.00000

439.00000 373.00000

458.00000 371.00000

477.00000 371.00000

496.00000 369.00000

514.00000 365.00000

533.00000 362.00000

552.00000 360.00000

571.00000 358.00000

590.00000 354.00000

607.00000 347.00000

624.00000 338.00000

640.00000 328.00000

656.00000 318.00000

673.00000 310.00000

690.00000 301.00000

704.00000 288.00000

716.00000 274.00000

731.00000 261.00000

747.00000 251.00000

761.00000 239.00000

776.00000 227.00000

788.00000 212.00000

803.00000 201.00000

813.00000 186.00000

829.00000 173.00000

845.00000 163.00000

863.00000 148.00000

862.00000 144.00000

855.00000 141.00000

836.00000 143.00000

820.00000 141.00000

782.00000 119.00000

765.00000 110.00000

749.00000 100.00000

735.00000 88.00000

719.00000 76.00000

703.00000 66.00000

686.00000 59.00000

668.00000 53.00000

650.00000 47.00000

631.00000 42.00000

613.00000 38.00000

594.00000 33.00000

576.00000 27.00000

558.00000 21.00000

540.00000 16.00000

521.00000 15.00000

502.00000 16.00000

483.00000 15.00000

464.00000 13.00000

445.00000 12.00000

426.00000 14.00000

407.00000 16.00000

388.00000 17.00000

369.00000 19.00000

351.00000 21.00000

332.00000 23.00000

314.00000 28.00000

296.00000 35.00000

278.00000 41.00000

259.00000 46.00000

242.00000 52.00000

224.00000 60.00000

207.00000 67.00000

190.00000 75.00000

173.00000 84.00000

156.00000 92.00000

138.00000 100.00000

123.00000 111.00000

106.00000 119.00000

89.00000 128.00000

76.00000 143.00000

66.00000 158.00000

54.00000 174.00000

41.00000 187.00000

IMAGE=20211126LSTR01.jpg

ID=71

SCALE=0.020832

LM=0

CURVES=1

POINTS=100

20.00000 169.00000

28.00000 148.00000

39.00000 128.00000

51.00000 108.00000

63.00000 89.00000

77.00000 71.00000

97.00000 59.00000

119.00000 53.00000

140.00000 46.00000

162.00000 39.00000

184.00000 33.00000

207.00000 29.00000

229.00000 26.00000

252.00000 24.00000

275.00000 25.00000

298.00000 26.00000

321.00000 29.00000

343.00000 33.00000

366.00000 37.00000

388.00000 40.00000

411.00000 45.00000

432.00000 52.00000

454.00000 58.00000

476.00000 65.00000

498.00000 71.00000

520.00000 77.00000

542.00000 83.00000

562.00000 94.00000

583.00000 104.00000

602.00000 116.00000

622.00000 128.00000

640.00000 142.00000

659.00000 154.00000

677.00000 168.00000

697.00000 179.00000

715.00000 194.00000

732.00000 208.00000

750.00000 223.00000

767.00000 238.00000

783.00000 255.00000

798.00000 271.00000

815.00000 287.00000

830.00000 304.00000

845.00000 321.00000

857.00000 341.00000

866.00000 362.00000

873.00000 384.00000

881.00000 405.00000

890.00000 426.00000

899.00000 447.00000

915.00000 474.00000

918.00000 488.00000

924.00000 497.00000

921.00000 504.00000

899.00000 508.00000

871.00000 509.00000

848.00000 515.00000

804.00000 525.00000

782.00000 530.00000

759.00000 536.00000

737.00000 542.00000

715.00000 547.00000

692.00000 549.00000

670.00000 552.00000

647.00000 552.00000

624.00000 552.00000

601.00000 553.00000

578.00000 551.00000

555.00000 551.00000

533.00000 550.00000

510.00000 545.00000

488.00000 539.00000

466.00000 533.00000

444.00000 528.00000

422.00000 523.00000

399.00000 518.00000

377.00000 512.00000

355.00000 506.00000

333.00000 499.00000

313.00000 490.00000

292.00000 480.00000

271.00000 471.00000

250.00000 461.00000

231.00000 449.00000

213.00000 435.00000

193.00000 424.00000

174.00000 411.00000

154.00000 400.00000

132.00000 393.00000

112.00000 382.00000

96.00000 366.00000

78.00000 352.00000

62.00000 336.00000

48.00000 317.00000

34.00000 299.00000

21.00000 281.00000

15.00000 259.00000

14.00000 236.00000

15.00000 213.00000

23.00000 192.00000

IMAGE=20211126LSTR02.jpg

ID=72

SCALE=0.020617

LM=0

CURVES=1

POINTS=100

56.00000 247.00000

61.00000 228.00000

67.00000 209.00000

72.00000 191.00000

77.00000 172.00000

82.00000 153.00000

88.00000 134.00000

99.00000 118.00000

114.00000 105.00000

130.00000 95.00000

146.00000 83.00000

165.00000 76.00000

184.00000 74.00000

203.00000 69.00000

222.00000 66.00000

242.00000 64.00000

261.00000 60.00000

280.00000 57.00000

299.00000 51.00000

318.00000 45.00000

337.00000 43.00000

355.00000 36.00000

375.00000 32.00000

394.00000 32.00000

414.00000 32.00000

433.00000 32.00000

453.00000 30.00000

472.00000 27.00000

492.00000 25.00000

511.00000 25.00000

531.00000 24.00000

550.00000 24.00000

570.00000 25.00000

589.00000 26.00000

608.00000 31.00000

627.00000 38.00000

645.00000 45.00000

663.00000 54.00000

680.00000 63.00000

697.00000 72.00000

714.00000 81.00000

732.00000 90.00000

749.00000 99.00000

767.00000 108.00000

783.00000 118.00000

801.00000 128.00000

816.00000 139.00000

832.00000 150.00000

846.00000 164.00000

860.00000 178.00000

871.00000 195.00000

860.00000 210.00000

849.00000 227.00000

836.00000 242.00000

824.00000 257.00000

811.00000 271.00000

796.00000 285.00000

780.00000 298.00000

766.00000 309.00000

751.00000 322.00000

737.00000 335.00000

722.00000 349.00000

707.00000 361.00000

690.00000 371.00000

673.00000 380.00000

655.00000 387.00000

636.00000 393.00000

617.00000 398.00000

598.00000 404.00000

580.00000 412.00000

562.00000 418.00000

543.00000 422.00000

523.00000 425.00000

504.00000 427.00000

484.00000 429.00000

465.00000 431.00000

445.00000 432.00000

426.00000 432.00000

406.00000 432.00000

387.00000 429.00000

368.00000 425.00000

348.00000 424.00000

328.00000 424.00000

309.00000 422.00000

289.00000 422.00000

270.00000 421.00000

251.00000 415.00000

232.00000 410.00000

213.00000 406.00000

194.00000 404.00000

175.00000 399.00000

157.00000 391.00000

139.00000 383.00000

122.00000 373.00000

107.00000 360.00000

95.00000 345.00000

85.00000 328.00000

76.00000 311.00000

66.00000 294.00000

58.00000 276.00000

IMAGE=20211126LSTR03.jpg

ID=73

SCALE=0.020201

LM=0

CURVES=1

POINTS=100

3172.00000 1307.00000

3154.00000 1308.00000

3138.00000 1314.00000

3121.00000 1318.00000

3104.00000 1320.00000

3086.00000 1318.00000

3070.00000 1310.00000

3057.00000 1298.00000

3047.00000 1285.00000

3038.00000 1269.00000

3030.00000 1254.00000

3022.00000 1238.00000

3014.00000 1222.00000

3005.00000 1207.00000

2998.00000 1191.00000

2991.00000 1175.00000

2988.00000 1158.00000

2978.00000 1144.00000

2968.00000 1130.00000

2958.00000 1115.00000

2951.00000 1099.00000

2943.00000 1083.00000

2934.00000 1068.00000

2928.00000 1051.00000

2923.00000 1035.00000

2917.00000 1018.00000

2910.00000 1002.00000

2903.00000 986.00000

2896.00000 970.00000

2889.00000 954.00000

2883.00000 938.00000

2876.00000 921.00000

2870.00000 905.00000

2865.00000 888.00000

2859.00000 872.00000

2853.00000 855.00000

2848.00000 839.00000

2844.00000 821.00000

2840.00000 804.00000

2836.00000 787.00000

2834.00000 770.00000

2835.00000 752.00000

2835.00000 735.00000

2835.00000 717.00000

2836.00000 700.00000

2837.00000 682.00000

2840.00000 665.00000

2846.00000 648.00000

2853.00000 632.00000

2863.00000 618.00000

2874.00000 605.00000

2888.00000 594.00000

2902.00000 585.00000

2903.00000 594.00000

2927.00000 610.00000

2940.00000 622.00000

2952.00000 635.00000

2962.00000 649.00000

2970.00000 665.00000

2980.00000 679.00000

2992.00000 693.00000

3003.00000 706.00000

3015.00000 719.00000

3026.00000 732.00000

3036.00000 747.00000

3044.00000 763.00000

3051.00000 779.00000

3060.00000 794.00000

3065.00000 811.00000

3071.00000 827.00000

3075.00000 844.00000

3079.00000 861.00000

3087.00000 877.00000

3097.00000 891.00000

3103.00000 907.00000

3109.00000 924.00000

3116.00000 940.00000

3125.00000 955.00000

3132.00000 971.00000

3139.00000 987.00000

3145.00000 1004.00000

3153.00000 1019.00000

3162.00000 1034.00000

3169.00000 1050.00000

3173.00000 1067.00000

3178.00000 1084.00000

3183.00000 1101.00000

3187.00000 1118.00000

3192.00000 1135.00000

3200.00000 1151.00000

3208.00000 1166.00000

3215.00000 1182.00000

3225.00000 1197.00000

3230.00000 1214.00000

3233.00000 1231.00000

3230.00000 1248.00000

3224.00000 1265.00000

3216.00000 1280.00000

3205.00000 1294.00000

3189.00000 1301.00000

IMAGE=20211126LTUA01.jpg

ID=74

SCALE=0.021177

LM=0

CURVES=1

POINTS=100

30.00000 274.00000

43.00000 291.00000

58.00000 305.00000

75.00000 317.00000

91.00000 330.00000

111.00000 336.00000

131.00000 341.00000

151.00000 348.00000

170.00000 357.00000

189.00000 365.00000

209.00000 371.00000

230.00000 372.00000

251.00000 374.00000

272.00000 375.00000

292.00000 376.00000

313.00000 375.00000

334.00000 373.00000

355.00000 371.00000

376.00000 371.00000

396.00000 368.00000

417.00000 363.00000

437.00000 358.00000

457.00000 354.00000

477.00000 347.00000

497.00000 342.00000

517.00000 334.00000

536.00000 326.00000

555.00000 318.00000

574.00000 309.00000

593.00000 300.00000

612.00000 291.00000

631.00000 282.00000

649.00000 271.00000

666.00000 261.00000

684.00000 249.00000

700.00000 236.00000

715.00000 221.00000

729.00000 206.00000

742.00000 190.00000

755.00000 173.00000

767.00000 156.00000

781.00000 141.00000

794.00000 125.00000

810.00000 111.00000

826.00000 97.00000

840.00000 82.00000

858.00000 73.00000

878.00000 65.00000

897.00000 57.00000

917.00000 51.00000

937.00000 45.00000

958.00000 39.00000

948.00000 35.00000

920.00000 37.00000

899.00000 40.00000

879.00000 44.00000

859.00000 48.00000

838.00000 52.00000

817.00000 52.00000

796.00000 52.00000

776.00000 50.00000

755.00000 46.00000

735.00000 41.00000

714.00000 36.00000

694.00000 33.00000

673.00000 30.00000

653.00000 27.00000

632.00000 24.00000

611.00000 23.00000

590.00000 24.00000

569.00000 26.00000

549.00000 27.00000

528.00000 30.00000

507.00000 33.00000

487.00000 36.00000

466.00000 41.00000

446.00000 45.00000

425.00000 49.00000

405.00000 53.00000

385.00000 58.00000

365.00000 66.00000

346.00000 74.00000

327.00000 82.00000

308.00000 90.00000

287.00000 95.00000

269.00000 105.00000

250.00000 113.00000

230.00000 119.00000

209.00000 124.00000

190.00000 132.00000

171.00000 140.00000

152.00000 149.00000

135.00000 161.00000

118.00000 173.00000

101.00000 185.00000

86.00000 200.00000

71.00000 214.00000

59.00000 232.00000

44.00000 245.00000

30.00000 261.00000

IMAGE=20211129LCDU01.jpg

ID=81

SCALE=0.020407

LM=0

CURVES=1

POINTS=100

603.00000 47.00000

616.00000 51.00000

628.00000 55.00000

641.00000 59.00000

654.00000 63.00000

667.00000 67.00000

679.00000 71.00000

690.00000 78.00000

701.00000 86.00000

714.00000 91.00000

727.00000 94.00000

739.00000 99.00000

750.00000 106.00000

761.00000 115.00000

771.00000 123.00000

781.00000 132.00000

790.00000 141.00000

800.00000 150.00000

810.00000 159.00000

820.00000 168.00000

830.00000 177.00000

839.00000 186.00000

849.00000 195.00000

859.00000 204.00000

869.00000 213.00000

879.00000 222.00000

889.00000 231.00000

899.00000 240.00000

909.00000 249.00000

917.00000 259.00000

926.00000 269.00000

933.00000 280.00000

942.00000 291.00000

950.00000 301.00000

958.00000 312.00000

967.00000 322.00000

975.00000 332.00000

982.00000 344.00000

989.00000 355.00000

995.00000 367.00000

1001.00000 379.00000

1009.00000 390.00000

1015.00000 401.00000

1022.00000 413.00000

1030.00000 424.00000

1038.00000 434.00000

1047.00000 444.00000

1053.00000 456.00000

1053.00000 463.00000

1041.00000 457.00000

1030.00000 450.00000

1017.00000 446.00000

1005.00000 440.00000

993.00000 435.00000

980.00000 432.00000

967.00000 431.00000

953.00000 430.00000

940.00000 428.00000

927.00000 425.00000

914.00000 423.00000

901.00000 418.00000

889.00000 415.00000

876.00000 410.00000

864.00000 405.00000

851.00000 400.00000

839.00000 395.00000

827.00000 389.00000

817.00000 381.00000

805.00000 375.00000

793.00000 368.00000

783.00000 360.00000

773.00000 351.00000

762.00000 343.00000

752.00000 334.00000

742.00000 326.00000

732.00000 317.00000

722.00000 308.00000

712.00000 299.00000

703.00000 290.00000

693.00000 280.00000

684.00000 270.00000

676.00000 260.00000

668.00000 250.00000

659.00000 240.00000

650.00000 229.00000

643.00000 218.00000

635.00000 207.00000

629.00000 195.00000

624.00000 183.00000

619.00000 171.00000

615.00000 158.00000

608.00000 147.00000

603.00000 134.00000

600.00000 121.00000

596.00000 109.00000

591.00000 96.00000

590.00000 83.00000

587.00000 70.00000

583.00000 57.00000

578.00000 45.00000

IMAGE=20211129LTEC01.jpg

ID=82

SCALE=0.035082

LM=0

CURVES=1

POINTS=100

65.00000 141.00000

79.00000 133.00000

93.00000 125.00000

107.00000 116.00000

121.00000 108.00000

135.00000 100.00000

148.00000 91.00000

162.00000 82.00000

177.00000 76.00000

191.00000 67.00000

204.00000 57.00000

218.00000 50.00000

234.00000 45.00000

249.00000 40.00000

265.00000 37.00000

281.00000 37.00000

297.00000 41.00000

312.00000 36.00000

328.00000 31.00000

342.00000 23.00000

357.00000 28.00000

373.00000 33.00000

389.00000 35.00000

405.00000 36.00000

421.00000 35.00000

438.00000 35.00000

453.00000 41.00000

468.00000 46.00000

484.00000 46.00000

500.00000 42.00000

516.00000 38.00000

532.00000 39.00000

548.00000 40.00000

563.00000 46.00000

580.00000 46.00000

595.00000 42.00000

611.00000 46.00000

626.00000 52.00000

640.00000 60.00000

655.00000 67.00000

670.00000 72.00000

685.00000 78.00000

701.00000 83.00000

715.00000 91.00000

730.00000 96.00000

747.00000 97.00000

763.00000 100.00000

779.00000 103.00000

795.00000 104.00000

811.00000 107.00000

800.00000 116.00000

785.00000 122.00000

770.00000 128.00000

755.00000 135.00000

742.00000 144.00000

727.00000 151.00000

714.00000 160.00000

699.00000 166.00000

684.00000 173.00000

670.00000 182.00000

657.00000 190.00000

644.00000 201.00000

630.00000 209.00000

615.00000 215.00000

600.00000 221.00000

585.00000 226.00000

569.00000 229.00000

552.00000 230.00000

536.00000 230.00000

520.00000 232.00000

504.00000 234.00000

488.00000 232.00000

472.00000 228.00000

456.00000 232.00000

440.00000 235.00000

424.00000 238.00000

408.00000 236.00000

393.00000 230.00000

377.00000 231.00000

361.00000 234.00000

345.00000 237.00000

329.00000 238.00000

313.00000 236.00000

299.00000 227.00000

284.00000 223.00000

268.00000 219.00000

252.00000 220.00000

236.00000 218.00000

220.00000 212.00000

205.00000 206.00000

189.00000 203.00000

173.00000 201.00000

158.00000 197.00000

143.00000 190.00000

129.00000 181.00000

116.00000 172.00000

101.00000 165.00000

85.00000 162.00000

69.00000 159.00000

53.00000 159.00000

IMAGE=20211201LDYO01.jpg

ID=83

SCALE=0.020201

LM=0

CURVES=1

POINTS=100

40.00000 179.00000

54.00000 168.00000

69.00000 158.00000

84.00000 148.00000

100.00000 139.00000

115.00000 131.00000

131.00000 123.00000

147.00000 114.00000

163.00000 105.00000

180.00000 99.00000

196.00000 91.00000

212.00000 84.00000

229.00000 77.00000

246.00000 72.00000

263.00000 67.00000

281.00000 64.00000

299.00000 61.00000

316.00000 57.00000

334.00000 54.00000

352.00000 53.00000

370.00000 52.00000

388.00000 51.00000

405.00000 50.00000

423.00000 50.00000

441.00000 51.00000

459.00000 52.00000

477.00000 52.00000

495.00000 52.00000

513.00000 52.00000

531.00000 52.00000

549.00000 54.00000

566.00000 57.00000

584.00000 59.00000

602.00000 61.00000

620.00000 64.00000

637.00000 68.00000

655.00000 71.00000

673.00000 75.00000

689.00000 81.00000

705.00000 89.00000

721.00000 97.00000

738.00000 105.00000

754.00000 113.00000

770.00000 120.00000

787.00000 126.00000

802.00000 135.00000

818.00000 145.00000

834.00000 152.00000

851.00000 158.00000

868.00000 163.00000

870.00000 168.00000

853.00000 176.00000

822.00000 181.00000

805.00000 185.00000

789.00000 194.00000

773.00000 202.00000

758.00000 211.00000

742.00000 221.00000

729.00000 232.00000

713.00000 241.00000

698.00000 251.00000

682.00000 258.00000

665.00000 265.00000

649.00000 272.00000

633.00000 281.00000

616.00000 286.00000

599.00000 292.00000

582.00000 296.00000

564.00000 298.00000

546.00000 301.00000

529.00000 306.00000

511.00000 309.00000

493.00000 312.00000

476.00000 315.00000

458.00000 317.00000

440.00000 319.00000

422.00000 319.00000

404.00000 318.00000

386.00000 316.00000

369.00000 313.00000

351.00000 311.00000

333.00000 309.00000

315.00000 307.00000

297.00000 305.00000

280.00000 301.00000

262.00000 297.00000

245.00000 293.00000

228.00000 286.00000

212.00000 280.00000

195.00000 274.00000

178.00000 268.00000

161.00000 262.00000

144.00000 256.00000

127.00000 249.00000

113.00000 238.00000

98.00000 228.00000

83.00000 218.00000

69.00000 207.00000

55.00000 196.00000

40.00000 186.00000

IMAGE=20211201LDYO02.jpg

ID=84

SCALE=0.020407

LM=0

CURVES=1

POINTS=100

47.00000 122.00000

64.00000 114.00000

80.00000 104.00000

97.00000 96.00000

113.00000 87.00000

130.00000 79.00000

147.00000 73.00000

165.00000 66.00000

182.00000 59.00000

199.00000 51.00000

217.00000 46.00000

235.00000 45.00000

253.00000 40.00000

271.00000 36.00000

289.00000 32.00000

308.00000 28.00000

326.00000 26.00000

345.00000 24.00000

363.00000 23.00000

382.00000 24.00000

401.00000 25.00000

419.00000 26.00000

438.00000 25.00000

456.00000 23.00000

475.00000 23.00000

494.00000 22.00000

512.00000 22.00000

531.00000 20.00000

549.00000 19.00000

568.00000 21.00000

586.00000 23.00000

605.00000 24.00000

623.00000 26.00000

642.00000 28.00000

660.00000 31.00000

679.00000 35.00000

697.00000 39.00000

715.00000 44.00000

733.00000 49.00000

751.00000 54.00000

769.00000 59.00000

786.00000 64.00000

804.00000 70.00000

822.00000 76.00000

839.00000 82.00000

857.00000 87.00000

875.00000 92.00000

900.00000 98.00000

920.00000 100.00000

922.00000 107.00000

918.00000 113.00000

880.00000 127.00000

858.00000 141.00000

839.00000 151.00000

826.00000 161.00000

811.00000 171.00000

795.00000 181.00000

778.00000 187.00000

760.00000 194.00000

743.00000 200.00000

725.00000 206.00000

707.00000 211.00000

689.00000 213.00000

671.00000 218.00000

652.00000 221.00000

634.00000 225.00000

616.00000 226.00000

597.00000 224.00000

578.00000 222.00000

560.00000 223.00000

543.00000 229.00000

525.00000 234.00000

507.00000 240.00000

488.00000 241.00000

470.00000 236.00000

452.00000 237.00000

434.00000 241.00000

415.00000 241.00000

396.00000 241.00000

378.00000 240.00000

359.00000 238.00000

341.00000 236.00000

322.00000 234.00000

304.00000 238.00000

285.00000 240.00000

267.00000 238.00000

248.00000 237.00000

230.00000 232.00000

213.00000 226.00000

195.00000 221.00000

177.00000 217.00000

159.00000 212.00000

140.00000 208.00000

123.00000 201.00000

107.00000 192.00000

92.00000 181.00000

76.00000 171.00000

63.00000 158.00000

47.00000 149.00000

32.00000 137.00000

IMAGE=20211201LDYO02BIS.jpg

ID=85

SCALE=0.020407

LM=0

CURVES=1

POINTS=100

86.00000 111.00000

99.00000 106.00000

112.00000 99.00000

125.00000 93.00000

138.00000 87.00000

149.00000 79.00000

161.00000 71.00000

173.00000 64.00000

185.00000 56.00000

198.00000 51.00000

212.00000 49.00000

225.00000 42.00000

238.00000 36.00000

251.00000 31.00000

265.00000 33.00000

279.00000 34.00000

293.00000 33.00000

307.00000 32.00000

321.00000 29.00000

335.00000 26.00000

349.00000 25.00000

363.00000 25.00000

378.00000 24.00000

392.00000 22.00000

406.00000 22.00000

420.00000 24.00000

434.00000 25.00000

448.00000 27.00000

462.00000 30.00000

476.00000 33.00000

490.00000 36.00000

504.00000 37.00000

518.00000 37.00000

532.00000 40.00000

546.00000 42.00000

560.00000 45.00000

574.00000 49.00000

587.00000 54.00000

600.00000 59.00000

613.00000 65.00000

626.00000 71.00000

639.00000 77.00000

652.00000 82.00000

665.00000 88.00000

678.00000 94.00000

691.00000 99.00000

704.00000 105.00000

718.00000 109.00000

732.00000 111.00000

746.00000 111.00000

756.00000 114.00000

756.00000 119.00000

748.00000 124.00000

710.00000 132.00000

697.00000 137.00000

684.00000 143.00000

670.00000 146.00000

657.00000 150.00000

643.00000 154.00000

630.00000 159.00000

616.00000 162.00000

603.00000 167.00000

589.00000 171.00000

575.00000 176.00000

562.00000 180.00000

548.00000 184.00000

534.00000 187.00000

520.00000 190.00000

506.00000 191.00000

493.00000 195.00000

479.00000 198.00000

465.00000 200.00000

451.00000 202.00000

437.00000 204.00000

423.00000 207.00000

409.00000 209.00000

395.00000 210.00000

380.00000 211.00000

366.00000 213.00000

352.00000 216.00000

338.00000 216.00000

324.00000 215.00000

310.00000 212.00000

296.00000 209.00000

282.00000 206.00000

268.00000 204.00000

254.00000 201.00000

240.00000 199.00000

227.00000 195.00000

213.00000 192.00000

200.00000 187.00000

186.00000 182.00000

173.00000 177.00000

160.00000 171.00000

148.00000 164.00000

136.00000 156.00000

124.00000 149.00000

112.00000 141.00000

99.00000 135.00000

86.00000 129.00000

IMAGE=20211201LDYO02TER.jpg

ID=86

SCALE=0.020201

LM=0

CURVES=1

POINTS=100

52.00000 203.00000

63.00000 188.00000

76.00000 174.00000

91.00000 162.00000

105.00000 148.00000

119.00000 135.00000

134.00000 123.00000

149.00000 112.00000

164.00000 99.00000

176.00000 85.00000

188.00000 70.00000

203.00000 59.00000

221.00000 52.00000

239.00000 48.00000

258.00000 41.00000

275.00000 35.00000

294.00000 33.00000

313.00000 31.00000

333.00000 32.00000

352.00000 32.00000

371.00000 33.00000

390.00000 34.00000

409.00000 35.00000

428.00000 35.00000

447.00000 36.00000

466.00000 36.00000

485.00000 37.00000

504.00000 37.00000

523.00000 43.00000

541.00000 48.00000

559.00000 54.00000

578.00000 59.00000

595.00000 66.00000

613.00000 72.00000

631.00000 79.00000

649.00000 86.00000

666.00000 95.00000

681.00000 106.00000

697.00000 117.00000

713.00000 128.00000

729.00000 139.00000

744.00000 150.00000

760.00000 161.00000

775.00000 172.00000

790.00000 185.00000

803.00000 198.00000

815.00000 213.00000

827.00000 228.00000

825.00000 242.00000

827.00000 252.00000

819.00000 270.00000

798.00000 296.00000

785.00000 309.00000

771.00000 323.00000

757.00000 336.00000

745.00000 350.00000

731.00000 364.00000

716.00000 375.00000

701.00000 387.00000

685.00000 398.00000

669.00000 408.00000

652.00000 416.00000

634.00000 423.00000

617.00000 432.00000

600.00000 440.00000

581.00000 445.00000

563.00000 450.00000

544.00000 454.00000

525.00000 457.00000

507.00000 462.00000

488.00000 465.00000

469.00000 467.00000

450.00000 470.00000

431.00000 472.00000

412.00000 473.00000

393.00000 472.00000

374.00000 471.00000

355.00000 470.00000

336.00000 467.00000

317.00000 463.00000

299.00000 459.00000

280.00000 454.00000

262.00000 449.00000

243.00000 443.00000

225.00000 437.00000

208.00000 428.00000

191.00000 419.00000

176.00000 408.00000

161.00000 396.00000

146.00000 384.00000

132.00000 371.00000

119.00000 357.00000

107.00000 342.00000

96.00000 327.00000

85.00000 311.00000

74.00000 295.00000

67.00000 278.00000

62.00000 259.00000

59.00000 240.00000

54.00000 222.00000

IMAGE=20211201LSTR01.jpg

ID=87

SCALE=0.020617

LM=0

CURVES=1

POINTS=100

114.00000 254.00000

116.00000 237.00000

117.00000 220.00000

119.00000 204.00000

125.00000 188.00000

130.00000 172.00000

135.00000 156.00000

142.00000 141.00000

150.00000 126.00000

160.00000 113.00000

173.00000 101.00000

188.00000 93.00000

203.00000 87.00000

220.00000 83.00000

236.00000 78.00000

251.00000 72.00000

267.00000 67.00000

284.00000 65.00000

301.00000 63.00000

317.00000 60.00000

334.00000 56.00000

350.00000 53.00000

367.00000 50.00000

384.00000 50.00000

401.00000 50.00000

417.00000 52.00000

434.00000 56.00000

450.00000 61.00000

466.00000 66.00000

482.00000 70.00000

498.00000 74.00000

514.00000 80.00000

530.00000 87.00000

544.00000 95.00000

559.00000 104.00000

573.00000 113.00000

588.00000 121.00000

602.00000 130.00000

617.00000 138.00000

631.00000 148.00000

644.00000 158.00000

657.00000 169.00000

670.00000 180.00000

682.00000 192.00000

693.00000 204.00000

701.00000 218.00000

713.00000 231.00000

728.00000 239.00000

741.00000 249.00000

754.00000 259.00000

767.00000 267.00000

766.00000 271.00000

748.00000 283.00000

731.00000 305.00000

719.00000 317.00000

708.00000 329.00000

693.00000 338.00000

680.00000 348.00000

666.00000 357.00000

652.00000 366.00000

640.00000 378.00000

625.00000 387.00000

610.00000 394.00000

595.00000 402.00000

581.00000 411.00000

565.00000 418.00000

549.00000 421.00000

533.00000 426.00000

517.00000 432.00000

501.00000 436.00000

485.00000 441.00000

469.00000 446.00000

453.00000 451.00000

436.00000 455.00000

420.00000 459.00000

403.00000 462.00000

387.00000 463.00000

370.00000 461.00000

353.00000 460.00000

336.00000 458.00000

320.00000 456.00000

303.00000 454.00000

286.00000 452.00000

270.00000 449.00000

253.00000 445.00000

238.00000 437.00000

223.00000 431.00000

206.00000 426.00000

191.00000 419.00000

177.00000 410.00000

164.00000 399.00000

154.00000 386.00000

144.00000 373.00000

131.00000 362.00000

121.00000 348.00000

114.00000 333.00000

108.00000 317.00000

107.00000 300.00000

105.00000 284.00000

107.00000 267.00000

IMAGE=20211201LSTR02.jpg

ID=88

SCALE=0.020614

LM=0

CURVES=1

POINTS=100

236.00000 159.00000

244.00000 154.00000

250.00000 148.00000

256.00000 141.00000

260.00000 133.00000

265.00000 125.00000

269.00000 117.00000

273.00000 109.00000

277.00000 101.00000

282.00000 93.00000

287.00000 86.00000

295.00000 81.00000

302.00000 76.00000

310.00000 72.00000

318.00000 68.00000

327.00000 65.00000

336.00000 63.00000

344.00000 59.00000

352.00000 56.00000

361.00000 53.00000

369.00000 49.00000

378.00000 46.00000

386.00000 45.00000

395.00000 43.00000

404.00000 42.00000

413.00000 40.00000

422.00000 38.00000

431.00000 36.00000

440.00000 36.00000

449.00000 34.00000

457.00000 32.00000

466.00000 31.00000

475.00000 27.00000

483.00000 25.00000

492.00000 25.00000

501.00000 27.00000

510.00000 29.00000

519.00000 31.00000

527.00000 34.00000

535.00000 38.00000

544.00000 41.00000

552.00000 45.00000

561.00000 47.00000

570.00000 50.00000

578.00000 54.00000

586.00000 56.00000

595.00000 59.00000

604.00000 62.00000

612.00000 66.00000

624.00000 72.00000

634.00000 75.00000

635.00000 77.00000

633.00000 80.00000

623.00000 85.00000

613.00000 93.00000

605.00000 98.00000

598.00000 104.00000

592.00000 110.00000

586.00000 117.00000

579.00000 123.00000

572.00000 129.00000

565.00000 134.00000

557.00000 138.00000

549.00000 142.00000

540.00000 146.00000

532.00000 150.00000

524.00000 153.00000

516.00000 157.00000

508.00000 161.00000

499.00000 164.00000

491.00000 168.00000

482.00000 171.00000

474.00000 174.00000

465.00000 177.00000

457.00000 179.00000

448.00000 182.00000

439.00000 183.00000

430.00000 185.00000

421.00000 186.00000

412.00000 188.00000

403.00000 189.00000

394.00000 190.00000

385.00000 191.00000

377.00000 192.00000

368.00000 193.00000

358.00000 193.00000

349.00000 193.00000

340.00000 193.00000

332.00000 191.00000

323.00000 188.00000

314.00000 186.00000

305.00000 184.00000

297.00000 182.00000

288.00000 179.00000

279.00000 176.00000

271.00000 172.00000

263.00000 168.00000

255.00000 165.00000

246.00000 164.00000

237.00000 162.00000

IMAGE=20211201LTEC01.jpg

ID=89

SCALE=0.030300

LM=0

CURVES=1

POINTS=100

95.00000 154.00000

106.00000 140.00000

120.00000 127.00000

135.00000 118.00000

152.00000 109.00000

167.00000 100.00000

184.00000 92.00000

201.00000 85.00000

218.00000 79.00000

236.00000 75.00000

253.00000 70.00000

271.00000 66.00000

289.00000 63.00000

307.00000 60.00000

325.00000 58.00000

344.00000 56.00000

362.00000 55.00000

380.00000 53.00000

398.00000 53.00000

417.00000 52.00000

435.00000 51.00000

453.00000 50.00000

471.00000 50.00000

490.00000 51.00000

508.00000 52.00000

526.00000 52.00000

544.00000 55.00000

562.00000 59.00000

580.00000 63.00000

598.00000 66.00000

616.00000 70.00000

634.00000 74.00000

652.00000 77.00000

669.00000 84.00000

687.00000 87.00000

705.00000 90.00000

722.00000 94.00000

740.00000 100.00000

757.00000 107.00000

772.00000 116.00000

788.00000 125.00000

806.00000 131.00000

822.00000 139.00000

839.00000 146.00000

856.00000 152.00000

874.00000 154.00000

893.00000 153.00000

911.00000 152.00000

929.00000 156.00000

945.00000 163.00000

927.00000 168.00000

909.00000 169.00000

891.00000 173.00000

873.00000 176.00000

855.00000 177.00000

837.00000 177.00000

819.00000 183.00000

801.00000 187.00000

784.00000 192.00000

767.00000 198.00000

750.00000 205.00000

735.00000 216.00000

719.00000 224.00000

702.00000 232.00000

685.00000 239.00000

667.00000 243.00000

650.00000 249.00000

633.00000 255.00000

615.00000 258.00000

597.00000 262.00000

579.00000 267.00000

562.00000 272.00000

544.00000 275.00000

526.00000 277.00000

507.00000 278.00000

489.00000 278.00000

471.00000 276.00000

453.00000 274.00000

434.00000 274.00000

416.00000 275.00000

398.00000 276.00000

380.00000 276.00000

361.00000 276.00000

343.00000 276.00000

325.00000 276.00000

307.00000 275.00000

288.00000 273.00000

270.00000 271.00000

252.00000 268.00000

234.00000 265.00000

216.00000 261.00000

198.00000 257.00000

181.00000 251.00000

164.00000 244.00000

148.00000 236.00000

132.00000 227.00000

117.00000 217.00000

106.00000 202.00000

96.00000 187.00000

84.00000 173.00000

IMAGE=20211206LCDU01.jpg

ID=90

SCALE=0.020617

LM=0

CURVES=1

POINTS=100

132.00000 108.00000

136.00000 123.00000

140.00000 138.00000

146.00000 152.00000

152.00000 167.00000

161.00000 179.00000

171.00000 191.00000

182.00000 202.00000

196.00000 210.00000

210.00000 216.00000

224.00000 222.00000

239.00000 226.00000

254.00000 230.00000

270.00000 232.00000

285.00000 235.00000

300.00000 237.00000

316.00000 239.00000

331.00000 241.00000

347.00000 241.00000

362.00000 240.00000

378.00000 240.00000

393.00000 238.00000

409.00000 235.00000

424.00000 232.00000

439.00000 229.00000

454.00000 226.00000

469.00000 222.00000

485.00000 219.00000

499.00000 215.00000

514.00000 210.00000

529.00000 205.00000

544.00000 199.00000

558.00000 194.00000

573.00000 189.00000

587.00000 182.00000

601.00000 175.00000

615.00000 169.00000

629.00000 162.00000

643.00000 156.00000

657.00000 150.00000

671.00000 143.00000

685.00000 136.00000

699.00000 129.00000

712.00000 120.00000

725.00000 111.00000

738.00000 103.00000

750.00000 94.00000

764.00000 86.00000

777.00000 79.00000

791.00000 71.00000

804.00000 63.00000

817.00000 55.00000

830.00000 45.00000

844.00000 31.00000

836.00000 34.00000

822.00000 40.00000

808.00000 47.00000

793.00000 51.00000

778.00000 54.00000

763.00000 54.00000

747.00000 54.00000

732.00000 55.00000

716.00000 56.00000

701.00000 56.00000

685.00000 57.00000

670.00000 58.00000

654.00000 59.00000

639.00000 60.00000

623.00000 61.00000

607.00000 62.00000

592.00000 63.00000

577.00000 64.00000

561.00000 64.00000

545.00000 63.00000

530.00000 63.00000

514.00000 63.00000

499.00000 62.00000

483.00000 62.00000

468.00000 62.00000

452.00000 61.00000

437.00000 60.00000

421.00000 58.00000

406.00000 57.00000

390.00000 57.00000

375.00000 58.00000

359.00000 58.00000

344.00000 59.00000

328.00000 60.00000

313.00000 62.00000

297.00000 63.00000

282.00000 65.00000

266.00000 66.00000

251.00000 67.00000

235.00000 68.00000

220.00000 71.00000

206.00000 78.00000

192.00000 85.00000

178.00000 91.00000

163.00000 95.00000

148.00000 99.00000

IMAGE=20211206LEUC01.jpg

ID=91

SCALE=0.020617

LM=0

CURVES=1

POINTS=100

317.00000 275.00000

326.00000 275.00000

335.00000 275.00000

345.00000 274.00000

354.00000 275.00000

363.00000 277.00000

372.00000 279.00000

381.00000 281.00000

390.00000 283.00000

399.00000 285.00000

408.00000 287.00000

417.00000 289.00000

426.00000 290.00000

435.00000 290.00000

444.00000 293.00000

453.00000 297.00000

461.00000 300.00000

469.00000 304.00000

477.00000 309.00000

485.00000 314.00000

493.00000 319.00000

502.00000 322.00000

510.00000 326.00000

518.00000 330.00000

525.00000 336.00000

532.00000 343.00000

539.00000 349.00000

546.00000 355.00000

554.00000 360.00000

561.00000 365.00000

568.00000 371.00000

575.00000 378.00000

581.00000 384.00000

587.00000 392.00000

593.00000 398.00000

598.00000 406.00000

604.00000 413.00000

609.00000 421.00000

613.00000 429.00000

618.00000 437.00000

623.00000 445.00000

628.00000 452.00000

633.00000 460.00000

637.00000 469.00000

641.00000 477.00000

645.00000 485.00000

651.00000 492.00000

656.00000 500.00000

662.00000 507.00000

665.00000 514.00000

664.00000 519.00000

654.00000 519.00000

640.00000 513.00000

631.00000 511.00000

622.00000 514.00000

613.00000 515.00000

604.00000 516.00000

595.00000 515.00000

586.00000 513.00000

577.00000 510.00000

568.00000 508.00000

559.00000 506.00000

550.00000 505.00000

541.00000 502.00000

533.00000 498.00000

525.00000 493.00000

517.00000 488.00000

509.00000 484.00000

500.00000 481.00000

492.00000 477.00000

483.00000 474.00000

474.00000 471.00000

466.00000 467.00000

458.00000 463.00000

450.00000 459.00000

442.00000 454.00000

435.00000 448.00000

427.00000 442.00000

420.00000 437.00000

413.00000 431.00000

406.00000 425.00000

399.00000 418.00000

393.00000 411.00000

386.00000 405.00000

380.00000 399.00000

372.00000 394.00000

365.00000 388.00000

359.00000 381.00000

353.00000 374.00000

347.00000 366.00000

342.00000 359.00000

336.00000 352.00000

331.00000 344.00000

327.00000 336.00000

322.00000 328.00000

319.00000 319.00000

316.00000 311.00000

313.00000 302.00000

311.00000 293.00000

308.00000 284.00000

IMAGE=20211207LAPH01.jpg

ID=93

SCALE=0.030766

LM=0

CURVES=1

POINTS=100

85.00000 166.00000

80.00000 155.00000

83.00000 141.00000

95.00000 123.00000

106.00000 112.00000

118.00000 102.00000

131.00000 93.00000

145.00000 85.00000

158.00000 77.00000

172.00000 71.00000

188.00000 68.00000

203.00000 64.00000

218.00000 61.00000

234.00000 58.00000

249.00000 55.00000

265.00000 53.00000

280.00000 52.00000

296.00000 50.00000

312.00000 49.00000

327.00000 47.00000

343.00000 45.00000

358.00000 46.00000

374.00000 47.00000

390.00000 48.00000

405.00000 50.00000

421.00000 52.00000

436.00000 52.00000

452.00000 53.00000

468.00000 55.00000

483.00000 56.00000

499.00000 58.00000

514.00000 60.00000

529.00000 64.00000

545.00000 68.00000

560.00000 73.00000

575.00000 78.00000

589.00000 84.00000

604.00000 89.00000

619.00000 94.00000

634.00000 99.00000

648.00000 105.00000

662.00000 111.00000

677.00000 118.00000

691.00000 125.00000

705.00000 131.00000

720.00000 135.00000

735.00000 140.00000

750.00000 143.00000

771.00000 147.00000

777.00000 152.00000

759.00000 159.00000

745.00000 166.00000

731.00000 173.00000

717.00000 179.00000

703.00000 187.00000

689.00000 195.00000

677.00000 205.00000

664.00000 214.00000

652.00000 223.00000

639.00000 232.00000

625.00000 240.00000

611.00000 247.00000

598.00000 256.00000

585.00000 264.00000

571.00000 271.00000

556.00000 276.00000

541.00000 281.00000

527.00000 286.00000

511.00000 291.00000

496.00000 295.00000

481.00000 298.00000

465.00000 300.00000

450.00000 301.00000

434.00000 303.00000

419.00000 305.00000

403.00000 306.00000

387.00000 307.00000

372.00000 308.00000

356.00000 309.00000

340.00000 310.00000

325.00000 309.00000

309.00000 308.00000

294.00000 306.00000

278.00000 304.00000

262.00000 302.00000

247.00000 298.00000

232.00000 295.00000

217.00000 290.00000

202.00000 284.00000

188.00000 278.00000

173.00000 273.00000

159.00000 266.00000

145.00000 259.00000

132.00000 251.00000

119.00000 242.00000

108.00000 231.00000

98.00000 219.00000

83.00000 196.00000

79.00000 184.00000

82.00000 176.00000

IMAGE=20211207LCTN01.jpg

ID=94

SCALE=0.020404

LM=0

CURVES=1

POINTS=100

93.00000 220.00000

86.00000 205.00000

86.00000 188.00000

91.00000 172.00000

97.00000 156.00000

103.00000 141.00000

111.00000 126.00000

120.00000 112.00000

132.00000 100.00000

144.00000 89.00000

158.00000 79.00000

173.00000 71.00000

189.00000 65.00000

204.00000 58.00000

220.00000 53.00000

236.00000 48.00000

252.00000 46.00000

269.00000 44.00000

286.00000 42.00000

302.00000 41.00000

319.00000 42.00000

336.00000 44.00000

353.00000 46.00000

369.00000 47.00000

386.00000 46.00000

403.00000 46.00000

419.00000 49.00000

436.00000 52.00000

452.00000 55.00000

469.00000 59.00000

485.00000 63.00000

501.00000 67.00000

517.00000 72.00000

533.00000 78.00000

548.00000 85.00000

562.00000 94.00000

577.00000 103.00000

591.00000 112.00000

605.00000 121.00000

619.00000 129.00000

634.00000 138.00000

648.00000 147.00000

662.00000 156.00000

676.00000 166.00000

693.00000 179.00000

706.00000 193.00000

723.00000 204.00000

748.00000 212.00000

773.00000 216.00000

779.00000 220.00000

777.00000 225.00000

765.00000 228.00000

748.00000 231.00000

732.00000 236.00000

717.00000 243.00000

702.00000 251.00000

687.00000 258.00000

673.00000 267.00000

658.00000 275.00000

644.00000 284.00000

629.00000 294.00000

616.00000 303.00000

602.00000 311.00000

587.00000 319.00000

572.00000 327.00000

558.00000 335.00000

543.00000 344.00000

528.00000 351.00000

513.00000 358.00000

497.00000 363.00000

481.00000 368.00000

465.00000 373.00000

449.00000 378.00000

433.00000 383.00000

417.00000 387.00000

400.00000 391.00000

384.00000 394.00000

367.00000 396.00000

350.00000 396.00000

334.00000 397.00000

317.00000 397.00000

300.00000 397.00000

283.00000 395.00000

267.00000 392.00000

251.00000 388.00000

235.00000 383.00000

219.00000 377.00000

203.00000 373.00000

188.00000 365.00000

173.00000 357.00000

158.00000 350.00000

144.00000 341.00000

131.00000 330.00000

120.00000 317.00000

109.00000 304.00000

101.00000 290.00000

94.00000 275.00000

91.00000 258.00000

91.00000 241.00000

98.00000 226.00000

IMAGE=20211207LCTN02.jpg

ID=95

SCALE=0.020407

LM=0

CURVES=1

POINTS=100

1647.00000 1950.00000

1675.00000 1962.00000

1704.00000 1970.00000

1734.00000 1975.00000

1764.00000 1979.00000

1794.00000 1984.00000

1823.00000 1994.00000

1852.00000 2004.00000

1880.00000 2015.00000

1907.00000 2029.00000

1933.00000 2045.00000

1959.00000 2061.00000

1985.00000 2077.00000

2010.00000 2094.00000

2035.00000 2111.00000

2059.00000 2130.00000

2083.00000 2149.00000

2105.00000 2169.00000

2127.00000 2190.00000

2150.00000 2210.00000

2174.00000 2229.00000

2198.00000 2247.00000

2219.00000 2269.00000

2240.00000 2292.00000

2256.00000 2318.00000

2274.00000 2342.00000

2291.00000 2367.00000

2309.00000 2392.00000

2323.00000 2419.00000

2344.00000 2441.00000

2366.00000 2462.00000

2384.00000 2486.00000

2399.00000 2512.00000

2412.00000 2540.00000

2426.00000 2567.00000

2440.00000 2594.00000

2454.00000 2621.00000

2464.00000 2650.00000

2478.00000 2677.00000

2492.00000 2704.00000

2504.00000 2732.00000

2515.00000 2760.00000

2522.00000 2789.00000

2529.00000 2819.00000

2541.00000 2868.00000

2544.00000 2912.00000

2557.00000 2963.00000

2560.00000 2982.00000

2567.00000 3008.00000

2566.00000 3021.00000

2559.00000 3025.00000

2549.00000 3025.00000

2534.00000 2999.00000

2493.00000 2975.00000

2459.00000 2967.00000

2428.00000 2966.00000

2398.00000 2963.00000

2371.00000 2950.00000

2345.00000 2934.00000

2321.00000 2916.00000

2296.00000 2899.00000

2271.00000 2881.00000

2245.00000 2866.00000

2220.00000 2848.00000

2195.00000 2830.00000

2172.00000 2811.00000

2147.00000 2793.00000

2126.00000 2771.00000

2104.00000 2750.00000

2081.00000 2731.00000

2056.00000 2714.00000

2031.00000 2697.00000

2011.00000 2673.00000

1989.00000 2653.00000

1966.00000 2633.00000

1944.00000 2611.00000

1925.00000 2588.00000

1907.00000 2563.00000

1889.00000 2539.00000

1871.00000 2515.00000

1852.00000 2491.00000

1836.00000 2465.00000

1823.00000 2438.00000

1808.00000 2411.00000

1791.00000 2386.00000

1775.00000 2360.00000

1759.00000 2334.00000

1744.00000 2308.00000

1731.00000 2280.00000

1716.00000 2254.00000

1702.00000 2227.00000

1688.00000 2200.00000

1676.00000 2172.00000

1666.00000 2143.00000

1658.00000 2114.00000

1650.00000 2084.00000

1644.00000 2055.00000

1640.00000 2024.00000

1636.00000 1994.00000

1634.00000 1964.00000

IMAGE=20211209LAPH01.jpg

ID=96

SCALE=0.013889

LM=0

CURVES=1

POINTS=100

82.00000 113.00000

94.00000 104.00000

106.00000 95.00000

117.00000 86.00000

130.00000 78.00000

142.00000 70.00000

155.00000 62.00000

169.00000 57.00000

183.00000 54.00000

197.00000 50.00000

211.00000 45.00000

226.00000 43.00000

241.00000 42.00000

255.00000 43.00000

270.00000 41.00000

284.00000 36.00000

298.00000 34.00000

313.00000 33.00000

328.00000 33.00000

343.00000 32.00000

356.00000 26.00000

371.00000 24.00000

385.00000 27.00000

400.00000 30.00000

414.00000 33.00000

429.00000 31.00000

443.00000 35.00000

457.00000 39.00000

472.00000 41.00000

487.00000 44.00000

501.00000 47.00000

515.00000 51.00000

528.00000 59.00000

541.00000 66.00000

555.00000 69.00000

569.00000 74.00000

582.00000 81.00000

594.00000 90.00000

608.00000 96.00000

621.00000 101.00000

632.00000 112.00000

645.00000 119.00000

659.00000 123.00000

673.00000 128.00000

686.00000 135.00000

700.00000 140.00000

714.00000 143.00000

728.00000 148.00000

743.00000 150.00000

758.00000 150.00000

772.00000 149.00000

770.00000 154.00000

756.00000 160.00000

733.00000 163.00000

718.00000 165.00000

704.00000 167.00000

689.00000 171.00000

675.00000 172.00000

660.00000 173.00000

645.00000 176.00000

631.00000 175.00000

616.00000 179.00000

602.00000 184.00000

588.00000 186.00000

573.00000 189.00000

559.00000 194.00000

545.00000 198.00000

531.00000 203.00000

518.00000 210.00000

505.00000 217.00000

491.00000 220.00000

476.00000 221.00000

461.00000 223.00000

447.00000 225.00000

432.00000 229.00000

418.00000 227.00000

403.00000 225.00000

389.00000 223.00000

374.00000 227.00000

360.00000 230.00000

345.00000 228.00000

331.00000 225.00000

316.00000 221.00000

302.00000 220.00000

287.00000 220.00000

272.00000 219.00000

259.00000 213.00000

244.00000 209.00000

230.00000 206.00000

216.00000 200.00000

204.00000 192.00000

190.00000 187.00000

176.00000 182.00000

164.00000 174.00000

151.00000 167.00000

138.00000 159.00000

125.00000 152.00000

112.00000 146.00000

100.00000 136.00000

89.00000 127.00000

IMAGE=20211209LOLW01.jpg

ID=97

SCALE=0.020689

LM=0

CURVES=1

POINTS=100

57.00000 141.00000

69.00000 127.00000

82.00000 116.00000

97.00000 104.00000

110.00000 93.00000

125.00000 83.00000

139.00000 71.00000

155.00000 64.00000

172.00000 57.00000

190.00000 54.00000

208.00000 55.00000

226.00000 54.00000

244.00000 52.00000

260.00000 45.00000

274.00000 33.00000

288.00000 22.00000

305.00000 17.00000

323.00000 15.00000

341.00000 15.00000

359.00000 15.00000

377.00000 13.00000

395.00000 10.00000

413.00000 9.00000

431.00000 8.00000

449.00000 7.00000

467.00000 7.00000

485.00000 7.00000

503.00000 8.00000

520.00000 13.00000

538.00000 12.00000

556.00000 9.00000

574.00000 11.00000

591.00000 14.00000

608.00000 19.00000

626.00000 24.00000

643.00000 28.00000

661.00000 29.00000

679.00000 31.00000

697.00000 34.00000

714.00000 39.00000

730.00000 47.00000

748.00000 52.00000

765.00000 56.00000

783.00000 61.00000

800.00000 66.00000

818.00000 68.00000

836.00000 70.00000

854.00000 71.00000

871.00000 72.00000

898.00000 77.00000

884.00000 85.00000

867.00000 90.00000

849.00000 94.00000

832.00000 99.00000

815.00000 104.00000

800.00000 113.00000

783.00000 120.00000

768.00000 129.00000

754.00000 141.00000

739.00000 150.00000

721.00000 153.00000

704.00000 160.00000

689.00000 169.00000

674.00000 179.00000

658.00000 188.00000

642.00000 196.00000

625.00000 202.00000

608.00000 207.00000

590.00000 210.00000

573.00000 215.00000

556.00000 222.00000

540.00000 229.00000

523.00000 235.00000

505.00000 237.00000

487.00000 238.00000

469.00000 236.00000

451.00000 235.00000

434.00000 240.00000

417.00000 245.00000

399.00000 246.00000

381.00000 247.00000

363.00000 243.00000

345.00000 241.00000

327.00000 245.00000

310.00000 244.00000

292.00000 246.00000

274.00000 244.00000

256.00000 238.00000

239.00000 233.00000

222.00000 227.00000

205.00000 224.00000

187.00000 222.00000

169.00000 218.00000

152.00000 212.00000

136.00000 204.00000

120.00000 195.00000

105.00000 185.00000

90.00000 175.00000

79.00000 162.00000

64.00000 151.00000

IMAGE=2021129LOLW01.jpg

ID=98

SCALE=0.020547

LM=0

CURVES=1

POINTS=100

280.00000 118.00000

287.00000 130.00000

293.00000 142.00000

302.00000 153.00000

310.00000 163.00000

318.00000 175.00000

323.00000 187.00000

329.00000 199.00000

334.00000 212.00000

340.00000 224.00000

346.00000 237.00000

349.00000 250.00000

355.00000 262.00000

362.00000 274.00000

368.00000 286.00000

372.00000 299.00000

375.00000 313.00000

378.00000 326.00000

381.00000 339.00000

386.00000 352.00000

392.00000 364.00000

394.00000 378.00000

395.00000 391.00000

396.00000 405.00000

398.00000 418.00000

400.00000 432.00000

399.00000 446.00000

399.00000 459.00000

401.00000 473.00000

402.00000 486.00000

400.00000 500.00000

397.00000 513.00000

394.00000 526.00000

391.00000 540.00000

390.00000 553.00000

386.00000 566.00000

382.00000 579.00000

376.00000 592.00000

371.00000 604.00000

364.00000 616.00000

355.00000 626.00000

345.00000 635.00000

334.00000 645.00000

325.00000 654.00000

316.00000 664.00000

308.00000 675.00000

299.00000 686.00000

291.00000 697.00000

285.00000 709.00000

278.00000 725.00000

274.00000 742.00000

270.00000 748.00000

268.00000 743.00000

269.00000 729.00000

271.00000 708.00000

270.00000 695.00000

268.00000 681.00000

265.00000 668.00000

259.00000 656.00000

250.00000 645.00000

242.00000 635.00000

234.00000 624.00000

227.00000 612.00000

220.00000 600.00000

214.00000 588.00000

210.00000 575.00000

207.00000 561.00000

205.00000 548.00000

203.00000 534.00000

202.00000 521.00000

202.00000 507.00000

201.00000 493.00000

200.00000 480.00000

199.00000 466.00000

196.00000 453.00000

195.00000 439.00000

196.00000 426.00000

198.00000 412.00000

201.00000 399.00000

204.00000 385.00000

206.00000 372.00000

210.00000 359.00000

211.00000 345.00000

212.00000 332.00000

215.00000 318.00000

219.00000 305.00000

222.00000 292.00000

225.00000 279.00000

228.00000 265.00000

232.00000 252.00000

234.00000 239.00000

238.00000 226.00000

245.00000 214.00000

249.00000 201.00000

254.00000 188.00000

260.00000 176.00000

263.00000 163.00000

270.00000 151.00000

273.00000 138.00000

274.00000 124.00000

IMAGE=2021129LTEC02.jpg

ID=99

SCALE=0.030598

LM=0

CURVES=1

POINTS=100

146.00000 24.00000

160.00000 23.00000

173.00000 24.00000

187.00000 23.00000

201.00000 22.00000

215.00000 22.00000

228.00000 24.00000

242.00000 25.00000

256.00000 26.00000

269.00000 27.00000

283.00000 28.00000

297.00000 27.00000

310.00000 30.00000

323.00000 34.00000

336.00000 40.00000

348.00000 46.00000

361.00000 52.00000

373.00000 58.00000

385.00000 65.00000

397.00000 71.00000

409.00000 78.00000

421.00000 85.00000

431.00000 94.00000

443.00000 101.00000

454.00000 109.00000

465.00000 117.00000

476.00000 125.00000

488.00000 132.00000

500.00000 139.00000

512.00000 146.00000

523.00000 155.00000

534.00000 163.00000

543.00000 173.00000

553.00000 183.00000

561.00000 194.00000

570.00000 204.00000

579.00000 215.00000

587.00000 226.00000

595.00000 237.00000

603.00000 248.00000

611.00000 259.00000

618.00000 271.00000

624.00000 283.00000

629.00000 296.00000

632.00000 310.00000

637.00000 322.00000

643.00000 335.00000

649.00000 347.00000

655.00000 360.00000

662.00000 372.00000

669.00000 383.00000

672.00000 388.00000

668.00000 391.00000

634.00000 386.00000

621.00000 384.00000

607.00000 382.00000

593.00000 382.00000

579.00000 382.00000

566.00000 381.00000

552.00000 378.00000

539.00000 374.00000

526.00000 369.00000

513.00000 366.00000

500.00000 361.00000

487.00000 356.00000

474.00000 351.00000

462.00000 346.00000

449.00000 341.00000

436.00000 337.00000

423.00000 332.00000

410.00000 327.00000

398.00000 321.00000

387.00000 312.00000

376.00000 304.00000

364.00000 297.00000

353.00000 289.00000

341.00000 282.00000

329.00000 275.00000

318.00000 267.00000

306.00000 260.00000

295.00000 252.00000

284.00000 243.00000

275.00000 234.00000

265.00000 224.00000

254.00000 215.00000

243.00000 207.00000

233.00000 198.00000

223.00000 189.00000

214.00000 178.00000

206.00000 167.00000

198.00000 156.00000

189.00000 145.00000

182.00000 134.00000

178.00000 120.00000

173.00000 108.00000

167.00000 95.00000

160.00000 83.00000

154.00000 71.00000

146.00000 60.00000

139.00000 48.00000

IMAGE=2021129LTEC03.jpg

ID=100

SCALE=0.030926

LM=0

CURVES=1

POINTS=100

1704.00000 1361.00000

1681.00000 1352.00000

1658.00000 1343.00000

1634.00000 1335.00000

1611.00000 1328.00000

1588.00000 1318.00000

1567.00000 1305.00000

1547.00000 1290.00000

1529.00000 1273.00000

1516.00000 1252.00000

1505.00000 1230.00000

1493.00000 1208.00000

1482.00000 1186.00000

1469.00000 1165.00000

1459.00000 1142.00000

1447.00000 1120.00000

1438.00000 1097.00000

1431.00000 1074.00000

1424.00000 1050.00000

1416.00000 1026.00000

1408.00000 1003.00000

1400.00000 980.00000

1391.00000 956.00000

1384.00000 933.00000

1377.00000 909.00000

1369.00000 885.00000

1361.00000 862.00000

1358.00000 837.00000

1353.00000 813.00000

1347.00000 789.00000

1341.00000 765.00000

1339.00000 740.00000

1329.00000 718.00000

1322.00000 694.00000

1319.00000 669.00000

1316.00000 645.00000

1314.00000 620.00000

1313.00000 595.00000

1314.00000 571.00000

1315.00000 546.00000

1317.00000 521.00000

1318.00000 496.00000

1324.00000 472.00000

1331.00000 449.00000

1341.00000 426.00000

1354.00000 404.00000

1371.00000 387.00000

1407.00000 362.00000

1421.00000 335.00000

1419.00000 314.00000

1414.00000 303.00000

1426.00000 316.00000

1439.00000 337.00000

1459.00000 352.00000

1482.00000 359.00000

1506.00000 366.00000

1529.00000 376.00000

1549.00000 390.00000

1569.00000 406.00000

1586.00000 423.00000

1601.00000 443.00000

1614.00000 464.00000

1625.00000 486.00000

1636.00000 508.00000

1645.00000 531.00000

1655.00000 554.00000

1667.00000 576.00000

1674.00000 600.00000

1683.00000 623.00000

1689.00000 647.00000

1695.00000 671.00000

1702.00000 694.00000

1706.00000 719.00000

1714.00000 742.00000

1723.00000 766.00000

1729.00000 790.00000

1733.00000 814.00000

1736.00000 839.00000

1741.00000 863.00000

1740.00000 888.00000

1741.00000 912.00000

1745.00000 937.00000

1749.00000 961.00000

1753.00000 986.00000

1757.00000 1010.00000

1761.00000 1035.00000

1766.00000 1059.00000

1771.00000 1083.00000

1776.00000 1107.00000

1780.00000 1132.00000

1785.00000 1156.00000

1790.00000 1180.00000

1792.00000 1205.00000

1791.00000 1230.00000

1786.00000 1254.00000

1783.00000 1279.00000

1774.00000 1302.00000

1763.00000 1324.00000

1745.00000 1341.00000

1727.00000 1358.00000

IMAGE=CRP01.jpg

ID=101

SCALE=0.017351

LM=0

CURVES=1

POINTS=100

1683.00000 1240.00000

1664.00000 1265.00000

1648.00000 1291.00000

1626.00000 1313.00000

1604.00000 1335.00000

1584.00000 1359.00000

1561.00000 1379.00000

1533.00000 1393.00000

1505.00000 1407.00000

1476.00000 1419.00000

1448.00000 1431.00000

1417.00000 1437.00000

1388.00000 1447.00000

1359.00000 1458.00000

1330.00000 1470.00000

1300.00000 1476.00000

1269.00000 1480.00000

1239.00000 1487.00000

1208.00000 1492.00000

1177.00000 1493.00000

1146.00000 1497.00000

1115.00000 1499.00000

1084.00000 1500.00000

1053.00000 1502.00000

1022.00000 1505.00000

991.00000 1506.00000

960.00000 1507.00000

929.00000 1508.00000

898.00000 1509.00000

867.00000 1510.00000

836.00000 1511.00000

805.00000 1509.00000

774.00000 1506.00000

743.00000 1503.00000

712.00000 1500.00000

682.00000 1497.00000

651.00000 1492.00000

621.00000 1484.00000

591.00000 1477.00000

560.00000 1476.00000

530.00000 1467.00000

501.00000 1455.00000

472.00000 1444.00000

444.00000 1430.00000

421.00000 1409.00000

395.00000 1393.00000

368.00000 1378.00000

343.00000 1360.00000

316.00000 1344.00000

290.00000 1327.00000

269.00000 1320.00000

299.00000 1315.00000

324.00000 1284.00000

340.00000 1258.00000

359.00000 1234.00000

380.00000 1211.00000

404.00000 1190.00000

431.00000 1175.00000

458.00000 1161.00000

487.00000 1148.00000

515.00000 1136.00000

544.00000 1125.00000

573.00000 1115.00000

603.00000 1106.00000

634.00000 1100.00000

665.00000 1099.00000

695.00000 1092.00000

725.00000 1092.00000

756.00000 1094.00000

787.00000 1091.00000

818.00000 1087.00000

849.00000 1084.00000

880.00000 1080.00000

911.00000 1077.00000

942.00000 1075.00000

973.00000 1073.00000

1004.00000 1070.00000

1035.00000 1069.00000

1066.00000 1071.00000

1096.00000 1075.00000

1127.00000 1073.00000

1158.00000 1069.00000

1189.00000 1070.00000

1220.00000 1072.00000

1251.00000 1078.00000

1282.00000 1079.00000

1313.00000 1083.00000

1343.00000 1086.00000

1374.00000 1092.00000

1404.00000 1099.00000

1434.00000 1108.00000

1464.00000 1117.00000

1492.00000 1129.00000

1521.00000 1141.00000

1550.00000 1152.00000

1576.00000 1169.00000

1603.00000 1184.00000

1630.00000 1199.00000

1655.00000 1218.00000

1685.00000 1225.00000

IMAGE=CRP02.jpg

ID=102

SCALE=0.024190

LM=0

CURVES=1

POINTS=100

1524.00000 1733.00000

1545.00000 1712.00000

1568.00000 1692.00000

1592.00000 1674.00000

1619.00000 1660.00000

1647.00000 1647.00000

1676.00000 1638.00000

1705.00000 1630.00000

1734.00000 1623.00000

1764.00000 1617.00000

1794.00000 1615.00000

1824.00000 1615.00000

1854.00000 1615.00000

1884.00000 1618.00000

1914.00000 1624.00000

1944.00000 1631.00000

1973.00000 1640.00000

2001.00000 1650.00000

2030.00000 1659.00000

2059.00000 1667.00000

2088.00000 1675.00000

2117.00000 1684.00000

2146.00000 1693.00000

2175.00000 1702.00000

2204.00000 1709.00000

2233.00000 1718.00000

2261.00000 1729.00000

2289.00000 1741.00000

2317.00000 1752.00000

2346.00000 1762.00000

2374.00000 1773.00000

2402.00000 1785.00000

2429.00000 1798.00000

2456.00000 1812.00000

2482.00000 1826.00000

2509.00000 1841.00000

2535.00000 1857.00000

2560.00000 1873.00000

2585.00000 1891.00000

2608.00000 1910.00000

2629.00000 1931.00000

2648.00000 1955.00000

2665.00000 1980.00000

2679.00000 2007.00000

2691.00000 2034.00000

2701.00000 2063.00000

2712.00000 2091.00000

2722.00000 2120.00000

2732.00000 2148.00000

2747.00000 2174.00000

2754.00000 2185.00000

2724.00000 2179.00000

2694.00000 2181.00000

2666.00000 2192.00000

2638.00000 2202.00000

2608.00000 2209.00000

2581.00000 2222.00000

2554.00000 2234.00000

2525.00000 2243.00000

2495.00000 2249.00000

2465.00000 2252.00000

2435.00000 2252.00000

2405.00000 2250.00000

2375.00000 2245.00000

2345.00000 2240.00000

2315.00000 2235.00000

2285.00000 2229.00000

2256.00000 2224.00000

2226.00000 2218.00000

2196.00000 2211.00000

2167.00000 2206.00000

2137.00000 2201.00000

2108.00000 2193.00000

2078.00000 2186.00000

2049.00000 2179.00000

2019.00000 2173.00000

1990.00000 2164.00000

1961.00000 2156.00000

1932.00000 2148.00000

1903.00000 2140.00000

1874.00000 2130.00000

1845.00000 2121.00000

1817.00000 2110.00000

1789.00000 2100.00000

1760.00000 2090.00000

1732.00000 2078.00000

1706.00000 2063.00000

1679.00000 2050.00000

1654.00000 2033.00000

1630.00000 2015.00000

1607.00000 1995.00000

1585.00000 1974.00000

1564.00000 1953.00000

1546.00000 1928.00000

1529.00000 1903.00000

1516.00000 1876.00000

1506.00000 1847.00000

1500.00000 1817.00000

1499.00000 1787.00000

1507.00000 1758.00000

IMAGE=CRP03.jpg

ID=103

SCALE=0.016475

LM=0

CURVES=1

POINTS=100

1440.00000 1781.00000

1442.00000 1807.00000

1447.00000 1833.00000

1455.00000 1858.00000

1466.00000 1882.00000

1479.00000 1904.00000

1494.00000 1925.00000

1513.00000 1943.00000

1532.00000 1962.00000

1550.00000 1980.00000

1567.00000 2000.00000

1587.00000 2017.00000

1611.00000 2028.00000

1636.00000 2036.00000

1661.00000 2043.00000

1687.00000 2048.00000

1713.00000 2052.00000

1739.00000 2057.00000

1764.00000 2064.00000

1790.00000 2069.00000

1816.00000 2073.00000

1842.00000 2076.00000

1868.00000 2078.00000

1894.00000 2080.00000

1920.00000 2076.00000

1946.00000 2072.00000

1971.00000 2078.00000

1997.00000 2083.00000

2023.00000 2084.00000

2049.00000 2081.00000

2075.00000 2077.00000

2101.00000 2074.00000

2127.00000 2073.00000

2153.00000 2069.00000

2179.00000 2065.00000

2203.00000 2057.00000

2227.00000 2046.00000

2251.00000 2036.00000

2276.00000 2028.00000

2301.00000 2019.00000

2325.00000 2008.00000

2346.00000 1993.00000

2368.00000 1978.00000

2392.00000 1968.00000

2417.00000 1962.00000

2442.00000 1954.00000

2465.00000 1941.00000

2486.00000 1925.00000

2500.00000 1903.00000

2507.00000 1878.00000

2522.00000 1857.00000

2545.00000 1845.00000

2570.00000 1839.00000

2548.00000 1835.00000

2522.00000 1837.00000

2497.00000 1831.00000

2478.00000 1812.00000

2463.00000 1791.00000

2453.00000 1767.00000

2443.00000 1743.00000

2426.00000 1723.00000

2405.00000 1707.00000

2381.00000 1696.00000

2357.00000 1686.00000

2332.00000 1678.00000

2307.00000 1672.00000

2281.00000 1667.00000

2255.00000 1664.00000

2229.00000 1664.00000

2203.00000 1661.00000

2178.00000 1655.00000

2152.00000 1651.00000

2126.00000 1653.00000

2099.00000 1651.00000

2073.00000 1652.00000

2047.00000 1651.00000

2021.00000 1652.00000

1995.00000 1654.00000

1969.00000 1653.00000

1943.00000 1650.00000

1917.00000 1649.00000

1891.00000 1652.00000

1864.00000 1654.00000

1838.00000 1653.00000

1812.00000 1650.00000

1786.00000 1647.00000

1760.00000 1645.00000

1734.00000 1643.00000

1708.00000 1647.00000

1683.00000 1653.00000

1656.00000 1655.00000

1630.00000 1656.00000

1605.00000 1663.00000

1580.00000 1671.00000

1556.00000 1682.00000

1533.00000 1694.00000

1511.00000 1708.00000

1490.00000 1724.00000

1471.00000 1742.00000

1453.00000 1761.00000

IMAGE=CRP04.jpg

ID=104

SCALE=0.019006

LM=0

CURVES=1

POINTS=100

1380.00000 2234.00000

1408.00000 2220.00000

1438.00000 2214.00000

1469.00000 2209.00000

1500.00000 2211.00000

1530.00000 2218.00000

1560.00000 2223.00000

1591.00000 2229.00000

1620.00000 2239.00000

1649.00000 2249.00000

1676.00000 2265.00000

1704.00000 2279.00000

1731.00000 2294.00000

1758.00000 2309.00000

1785.00000 2324.00000

1811.00000 2342.00000

1835.00000 2361.00000

1861.00000 2378.00000

1887.00000 2394.00000

1915.00000 2408.00000

1941.00000 2425.00000

1966.00000 2443.00000

1989.00000 2464.00000

2013.00000 2484.00000

2037.00000 2503.00000

2064.00000 2518.00000

2090.00000 2535.00000

2115.00000 2554.00000

2140.00000 2572.00000

2164.00000 2591.00000

2187.00000 2612.00000

2211.00000 2631.00000

2234.00000 2652.00000

2256.00000 2675.00000

2276.00000 2698.00000

2296.00000 2722.00000

2311.00000 2749.00000

2327.00000 2775.00000

2344.00000 2801.00000

2361.00000 2827.00000

2371.00000 2856.00000

2378.00000 2886.00000

2385.00000 2917.00000

2388.00000 2948.00000

2387.00000 2979.00000

2382.00000 3009.00000

2379.00000 3040.00000

2384.00000 3070.00000

2397.00000 3098.00000

2415.00000 3124.00000

2420.00000 3140.00000

2396.00000 3120.00000

2370.00000 3104.00000

2341.00000 3092.00000

2311.00000 3089.00000

2280.00000 3093.00000

2250.00000 3098.00000

2219.00000 3099.00000

2188.00000 3093.00000

2158.00000 3086.00000

2128.00000 3083.00000

2098.00000 3074.00000

2069.00000 3063.00000

2041.00000 3050.00000

2014.00000 3035.00000

1989.00000 3017.00000

1963.00000 3000.00000

1935.00000 2985.00000

1911.00000 2967.00000

1886.00000 2948.00000

1861.00000 2930.00000

1836.00000 2912.00000

1811.00000 2893.00000

1786.00000 2875.00000

1761.00000 2857.00000

1735.00000 2839.00000

1710.00000 2821.00000

1687.00000 2800.00000

1664.00000 2780.00000

1639.00000 2761.00000

1615.00000 2741.00000

1592.00000 2721.00000

1568.00000 2700.00000

1546.00000 2679.00000

1524.00000 2657.00000

1502.00000 2636.00000

1479.00000 2614.00000

1460.00000 2590.00000

1441.00000 2566.00000

1421.00000 2542.00000

1402.00000 2517.00000

1387.00000 2490.00000

1374.00000 2462.00000

1364.00000 2433.00000

1354.00000 2403.00000

1350.00000 2372.00000

1349.00000 2342.00000

1353.00000 2311.00000

1357.00000 2280.00000

1357.00000 2249.00000

IMAGE=CRP05.jpg

ID=105

SCALE=0.017401

LM=0

CURVES=1

POINTS=100

1539.00000 3479.00000

1539.00000 3478.00000

1539.00000 3476.00000

1539.00000 3475.00000

1540.00000 3473.00000

1541.00000 3473.00000

1542.00000 3472.00000

1544.00000 3472.00000

1545.00000 3472.00000

1547.00000 3472.00000

1548.00000 3472.00000

1549.00000 3472.00000

1551.00000 3472.00000

1552.00000 3472.00000

1554.00000 3472.00000

1555.00000 3472.00000

1557.00000 3472.00000

1558.00000 3472.00000

1560.00000 3472.00000

1561.00000 3472.00000

1562.00000 3472.00000

1564.00000 3472.00000

1565.00000 3472.00000

1567.00000 3472.00000

1568.00000 3472.00000

1570.00000 3472.00000

1571.00000 3472.00000

1572.00000 3472.00000

1574.00000 3472.00000

1575.00000 3472.00000

1577.00000 3472.00000

1579.00000 3473.00000

1580.00000 3473.00000

1581.00000 3474.00000

1582.00000 3474.00000

1584.00000 3475.00000

1585.00000 3475.00000

1586.00000 3476.00000

1588.00000 3476.00000

1589.00000 3477.00000

1590.00000 3477.00000

1592.00000 3478.00000

1593.00000 3479.00000

1594.00000 3479.00000

1595.00000 3480.00000

1597.00000 3481.00000

1598.00000 3482.00000

1599.00000 3482.00000

1600.00000 3483.00000

1601.00000 3484.00000

1602.00000 3485.00000

1603.00000 3486.00000

1604.00000 3487.00000

1605.00000 3488.00000

1604.00000 3490.00000

1603.00000 3490.00000

1602.00000 3490.00000

1600.00000 3490.00000

1599.00000 3490.00000

1597.00000 3489.00000

1596.00000 3489.00000

1595.00000 3489.00000

1593.00000 3489.00000

1592.00000 3489.00000

1590.00000 3488.00000

1589.00000 3488.00000

1587.00000 3488.00000

1586.00000 3488.00000

1585.00000 3488.00000

1583.00000 3487.00000

1582.00000 3487.00000

1580.00000 3487.00000

1579.00000 3487.00000

1577.00000 3487.00000

1576.00000 3487.00000

1575.00000 3487.00000

1573.00000 3486.00000

1572.00000 3486.00000

1570.00000 3486.00000

1569.00000 3486.00000

1567.00000 3486.00000

1566.00000 3486.00000

1565.00000 3486.00000

1563.00000 3486.00000

1562.00000 3486.00000

1560.00000 3486.00000

1559.00000 3486.00000

1557.00000 3486.00000

1556.00000 3486.00000

1555.00000 3486.00000

1553.00000 3486.00000

1552.00000 3486.00000

1550.00000 3486.00000

1549.00000 3486.00000

1547.00000 3485.00000

1546.00000 3485.00000

1545.00000 3484.00000

1543.00000 3484.00000

1542.00000 3483.00000

1541.00000 3482.00000

IMAGE=NEW01.jpg

ID=106

SCALE=0.007487
